# A systematic review and meta-analysis on the prevalence of non-malignant, organic gastrointestinal disorders misdiagnosed as irritable bowel syndrome

**DOI:** 10.1038/s41598-022-05933-1

**Published:** 2022-02-04

**Authors:** Dennis Poon, Graham R. Law, Giles Major, H. Jervoise N. Andreyev

**Affiliations:** 1 Department of Gastroenterology, Lincoln County Hospital, Greetwell Road, Lincoln, LN2 5QY UK; 2 Community and Health Research Unit, School of Health and Social Care, University of Lincoln, Lincoln, LN6 7TS UK; 3 NIHR Nottingham Biomedical Research Centre, Nottingham University Hospitals NHS Trust and the University of Nottingham, Nottingham, UK; 4 Queens Medical Centre Campus, Derby Road, Nottingham, NG7 2UH UK

**Keywords:** Irritable bowel syndrome, Diarrhoea, Irritable bowel syndrome

## Abstract

Treatable gastrointestinal disorders in patients with symptoms typical for irritable bowel syndrome (IBS) may be overlooked. The prevalence of five gastrointestinal conditions—bile acid diarrhoea (BAD), carbohydrate malabsorption (CM), microscopic colitis (MC), pancreatic exocrine insufficiency (PEI) and small intestinal bacterial overgrowth (SIBO) was systematically assessed from studies including consecutive patients meeting diagnostic criteria for IBS. 4 databases were searched from 1978 to 2020. Studies were included if they evaluated the prevalence of these conditions in secondary healthcare setting. Estimated pooled rates were calculated and statistical heterogeneity between studies was evaluated using Q and I^2^ statistics. Seven studies (n = 597) estimated the pooled prevalence for BAD as 41% (95% CI 29–54). 17 studies (n = 5068) estimated that of MC as 3% (95% CI 2–4%). Two studies (n = 478) suggested a rate of 4.6% (range: 1.8–6.1%) for PEI. Using breath testing, 26 studies (n = 6700) and 13 studies (n = 3415) estimated the prevalence of lactose and fructose malabsorption as 54% (95% CI 44–64%) and 43% (95% CI 23–62%); 36 studies (n = 4630) and 22 studies (n = 2149) estimated that of SIBO as 49% (95% CI 40–57%) with lactulose and 19% (95% CI 13–27%) with glucose. Rates of all conditions were significantly higher than in healthy controls. A significant proportion of patients presenting to secondary care with IBS have an organic condition which may account for their symptoms. Failure to exclude such conditions will deny patients effective treatment.

## Introduction

Irritable bowel syndrome (IBS) is defined by a symptom cluster. Cluster content has changed over time but includes recurrent, defecation-associated abdominal pain and change in bowel habit. There is no biological marker for IBS^1^. Patients with IBS-like symptoms are the single largest group of patients presenting with gastrointestinal (GI) complaints to both primary and secondary healthcare^2,3^. IBS has been estimated to affect at least 7–21% of the global adult population^4^.

Terms such as ‘irritable colon syndrome’, ‘spastic colon’, ‘painless diarrhoea’ and ‘mucus colitis’ were used until the mid-twentieth century, with classification defined subjectively by clinical experience^5–8^. The Manning and Kruis criteria were developed, in 1978 and 1984 respectively, to rationalise the use of investigations and minimise surgery prior to diagnosis of IBS^7,8^. Neither of these criteria was adequate for clinical practice and they were superseded by the consensus-based Rome criteria^9^. One aim of such criteria was to promote a positive diagnosis of functional GI disorder where no biological or structural cause could be identified^10^. This standardised definition has facilitated research into the underlying pathophysiology of IBS but successful translation into therapy has thus far been limited. The Rome criteria have been developed using community populations rather than patients selected for secondary care referral and assessment.

The current guidelines recommend that patients can be diagnosed with IBS, in the absence of ‘red flags’, when: their symptoms fulfil the Rome IV criteria for IBS; their full blood count, coeliac serology, C reactive protein and erythrocyte sedimentation rate are normal; and they have a faecal calprotectin level of < 50 µg/g^11,12^. However, many physicians find these diagnostic criteria too restrictive and unclear^13^. Additionally, this diagnostic approach has only modest ability in differentiating IBS from other diseases^14,15^. This may explain the low proportion of patients responding to treatments for IBS^16^.

Gastroenterologists are aware of coeliac disease, inflammatory bowel disease and colorectal cancer as possible confounding diagnoses in up to 8.6% of patients who meet clinically defined criteria of IBS^17–22^. These conditions are often associated with alarming symptoms which prompt further investigations, allowing them to be diagnosed. In this systematic review we aimed to clarify the prevalence of five other, often overlooked, conditions—bile acid diarrhoea (BAD), carbohydrate malabsorption (CM), microscopic colitis (MC), pancreatic exocrine insufficiency (PEI) and small intestinal bacterial overgrowth (SIBO) in adults presenting to secondary care with IBS-like symptoms.

## Methods

### Search strategy

This systematic review and meta-analysis was performed in accordance with PRISMA recommendations. The PRISMA checklist for this study can be found in the “Supplementary Material S1”. The literature search was conducted using CINAHL, Cochrane, Embase and Pubmed from 1978, the year of publication of the Manning criteria, to November 2020. Literature searches were conducted individually for 5 conditions. The terms ‘*irritable bowel syndrome’* OR *‘irritable colon’* OR *‘IBS’* OR *‘functional bowel’* OR *‘diarrhoea’* OR *‘diarrhea’* were used as medical subject heading (MeSH) and free text terms for each condition.

These were combined using the set operator AND with following terms:

- For BAD
  *‘bile acid malabsorption’* OR *‘bile salt malabsorption’* OR *‘BAM’* OR ‘*BAD*’ OR *‘bile acid’* OR *‘bile salt’* OR *‘idiopathic bile acid’* OR *‘SeHCAT’* OR *‘*^*75*^*Selenium homotaurocholic acid test’* OR ‘faecal bile acid test’ OR *‘fecal bile acid test’* OR *‘7 α-hydroxy-4-cholesten-3-one*’ OR ‘*C4’* OR *‘fibroblast growth factor 19’* OR *‘FGF19’*.
- For CM
  ‘*carbohydrate’* OR ‘*monosaccharide’* OR *‘disaccharide’* OR *‘oligosaccharide’* OR *‘polyol’* OR *’lactose’* OR *‘fructose’ OR ‘sucrose’* OR *‘sorbitol’* OR *‘mannitol’* AND *‘malabsorption’* OR *‘intolerance’.*
- For MC
  *‘microscopic colitis’* OR *‘lymphocytic colitis’* OR *‘collagenous colitis’.*
- For PEI
  *‘pancreatic exocrine insufficiency’* OR *‘pancreatic insufficiency’* OR *‘pancreatic function’* OR *‘faecal elastase’* OR *‘fecal elastase’* OR ‘*breath test*’ OR *‘secretin’* OR *‘cholecystokinin’* OR *‘pancreatic enzyme replacement therapy’.*
- For SIBO
  ‘*small intestinal bacterial overgrowth’* OR *‘small bowel bacterial overgrowth’ OR ‘SIBO’* OR *‘SBBO’* OR *‘bacteria overgrowth’* OR *‘breath test’* OR *‘lactulose hydrogen’* OR *‘glucose hydrogen’* OR *‘sucrose hydrogen’* OR *‘xylose hydrogen’* OR *‘jejunal aspirate’.*

The searches were restricted to patients over the age of 18 years and published in English. The bibliographies of eligible studies were searched for additional studies.

### Study selection

Studies were included if they assessed prevalence of any of these conditions in consecutive patients meeting the Manning, Kruis or Rome I–IV criteria system in a secondary healthcare setting. We have included Manning and Kruis criteria as part of our search criteria because although they are rarely acknowledged formally nowadays, what many clinicians do in practice closely resembles the approach taken by Manning and Kruis. Studies whose primary aim was not evaluating prevalence of the conditions, but presented these data, were also included. Studies with zero cases were also included in the analysis.

Important reasons to exclude studies were: primary care studies; lack of prevalence data; no clear definitions of IBS; inclusion of individuals with any other known organic GI diseases (coeliac disease, inflammatory disease, etc.) or previous abdominal surgery; only reported as conference abstracts with no full text available.

The titles of all papers identified from the literature searches were scanned and abstracts of potentially relevant papers were reviewed. The full text of any papers meeting the inclusion criteria were obtained. Where data were missing within the eligible studies, the authors were contacted. Studies were assessed using the pre-defined eligibility criteria independently by two reviewers (DP and HJNA). Any discrepancies were resolved by consensus.

The protocol used for this systematic review was registered with PROSPERO, number CRD 42019145486.

### Quality assessment of the individual studies

Quality and risk of bias of the included studies were analysed independently by two reviewers (DP and HJNA) using Critical Appraisal Skills Programme (CASP) checklist for cohort studies^23^.

We were unable to identify a single appraisal tool which was applicable to all studies. Some items of this checklist were not applicable to the non-cohort studies that presented prevalence data. We modified the checklist by removing items 6, 7 and 8 regarding length of follow-up and precision of study results. This led to a maximum score of ten points, one for each item on the checklist. A score ≤ 7 points indicated high risk of bias. Appraisal for the included studies can be found in Supplementary Tables 1–5.

Any discrepancies were resolved by consensus, a third reviewer was consulted if consensus was not reached.

Analyses were repeated after excluding studies at high risk of bias and can be found in the “Supplementary Material S1”.

### Data extraction

All relevant data were extracted onto a Microsoft Excel spreadsheet (Office 365 for Mac, Microsoft Corp, Redmond, WA, USA). The principal study outcome was prevalence of each of the five conditions (BAD, CM, MC, PEI, SIBO) in patients fulfilling IBS criteria. The five conditions were defined as follows.

- BAD
  - 75-selenium homocholic acid taurine (SeHCAT) scan with 7-day retention < 15% (severe BAD is defined as SeHCAT < 5%; moderate as SeHCAT < 10%; mild as SeHCAT < 15%)^24^, or
  - total 48-h faecal bile acids > 2,337 μmol^25^, or
  - an elevated fasting 7α-hydroxy-4-cholesten-3-one (7α-C4) level
- CM
  - a positive lactose, fructose, sucrose, sorbitol or mannitol hydrogen breath test, or
  - genotyping for adult-type hypolactasia leading to lactose intolerance
- MC
  - abnormal histological findings on colonic biopsies meeting criteria for lymphocytic or collagenous colitis
- PEI
  - faecal elastase-1 level < 200 μg/g^26^
- SIBO
  - a positive lactulose or glucose hydrogen breath test, or
  - a positive bacterial culture or a bacterial count of > 10^3^ colony-forming units (cfu) per ml in the small intestinal aspirate^27^

The total number of patients with IBS were extracted from each study (denominator), followed by the number of those patients diagnosed with an organic condition using one of the recognised diagnostic tests above (numerator). The prevalence was expressed as the proportion of patients meeting IBS criteria, who underwent one of the above tests, had an organic condition.

Additionally, subgroup analyses for each condition were performed incorporating: only studies with a sample size ≥ 100; studies utilising Rome as the diagnostic criteria for IBS; and prospective studies. In addition, we included the study location and specific aspects of the tests or criteria used for diagnosing the organic condition, e.g. dose of test substrate used in breath testing for CM and SIBO; number of lymphocytes per 100 epithelial cells to define lymphocytic colitis; or thickness of sub-epithelial collagen to define collagenous colitis on histological examination.

All subgroup analyses can be found in the “Supplementary Material S1”.

### Data synthesis and statistical analysis

We performed statistical analysis on the extracted data using Stata SE 16 (StataCorp LLC, USA). Crude pooled prevalence for each condition was obtained by combining, from all studies, the prevalence proportions as described above. Each condition is diagnosed using different tests so prevalence was presented individually, based on the diagnostic modality used. Estimated pooled rates with 95% confidence intervals calculated using random effects with DerSimonian–Laird method^28^ were presented in forest plots. Statistical heterogeneity between studies was evaluated using Q and I^2^ statistics^29^, with I^2^ > 50% indicating significant heterogeneity^30^. Subgroup analysis by study characteristics was performed to explore any heterogeneity observed. When there were ten or more studies in the meta-analysis, we undertook a general inspection of funnel plots to assess for small-study effects and publication bias^31,32^.

## Results

### Search results

### Bile acid diarrhoea (BAD)

3173 citations were identified. Of these, 94 were potentially relevant and the full text of these citations were retrieved for full evaluation. 15 papers were included, 79 papers did not satisfy the eligibility criteria (Supplementary Fig. 1).

Nine studies reported the prevalence of BAD based upon an abnormal SeHCAT scan. Two studies used elevated faecal bile acids over 48 h and one study used an elevated serum 7α-C4 level. The remaining three studies used two diagnostic tests (SeHCAT scan and 7α-C4 level, total 48-h faecal bile acids and 7α-C4 level).

### Prevalence of a positive SeHCAT scan in patients fulfilling IBS criteria

The ten studies that reported the prevalence of BAD based upon an abnormal SeHCAT study are shown in Supplementary Table 1. Across the ten studies, seven different 7-day SeHCAT retention cut-off values (i.e. < 5%, < 10%, < 11%, < 11.7%, < 15%,) were used. The two studies which had reported values within 2% of the < 10% cut-off (i.e. < 8 to < 12%) were included in the prevalence calculation of the 7-day SeHCAT < 10% subgroup. Six papers^33–38^ demonstrated the change in their rates of positive scans by using different cut-off values but only one^36^ assessed treatment response to cholestyramine by comparing with placebo. Nevertheless, a previous systematic review showed that the response rate to bile acid sequestrants was higher in patients with severe BAD than those with mild or moderate disease^24^. One study^38^ evaluated and compared the prevalence of BAD with different SeHCAT retention values as a result of using the Rome III and Rome IV criteria, respectively, to define their patients.

### 7-day SeHCAT retention < 5% (severe BAD)

**Figure 1:**
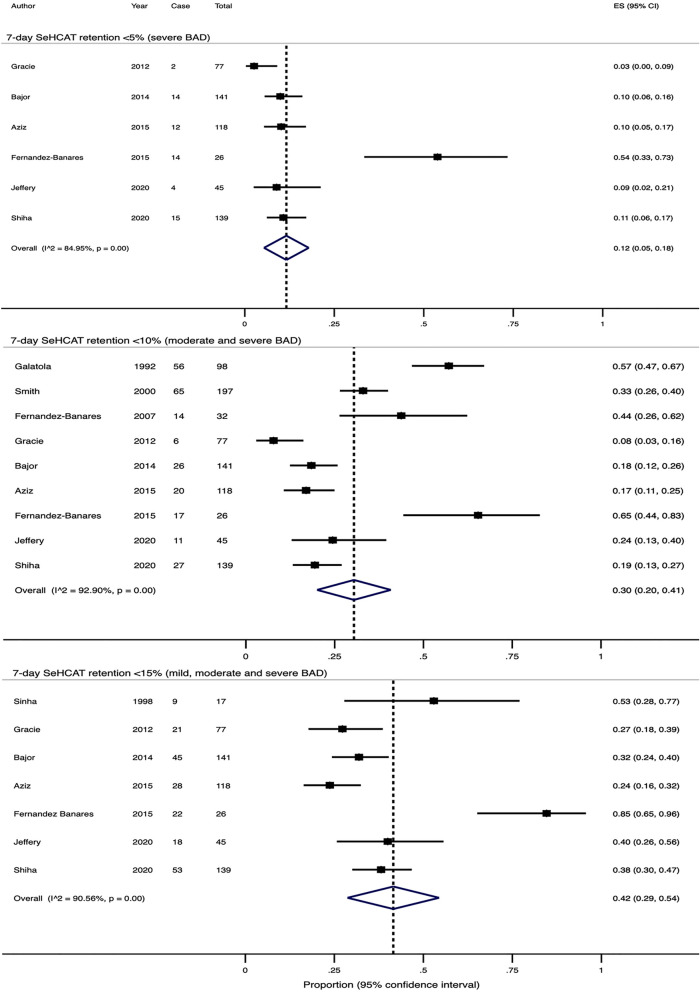
Forest plots showing the estimated pooled prevalence of BAD diagnosed with SeHCAT in patients fulfilling IBS criteria, if the Rome IV criteria were used by Shiha et al.

Six studies reported prevalence using a 7-day cut-off value of < 5%. If the Rome III criteria were used in the paper by Shiha et al., the crude pooled rate would be 10.7% (range 2.6–53.8%) out of 580 patients and the estimated pooled rate would be 11% (95% CI 5–17%, Supplementary Fig. 2) by the random effects model. If the Rome IV criteria were used instead, the crude pooled rate would be 11.1% (range 2.6–53.8%) out of 546 patients and the estimated pooled rate would be 12% (95% CI 5–18%, Fig. 1). There was significant heterogeneity in both effect sizes (Q-test X^2^ = 32.4, P < 0.0001; I^2^ = 84.5% and Q-test X^2^ = 33.2, P < 0.0001; I^2^ = 85.0%).

### 7-day SeHCAT retention < 10% (moderate and severe BAD)

Seven studies reported prevalence using a 7-day cut-off value of < 10%. Additionally, two studies reported the prevalence using < 11% and < 11.7% as the cut-off. If the Rome III criteria were used in the paper by Shiha et al., the crude pooled rate would be 27.2% (range 7.8–65.3%) out of 907 patients and the estimated pooled rate would be 30% (95% CI 20–40%, Supplementary Fig. 2) by the random effects model. If the Rome IV criteria were used instead, the crude pooled rate would be 27.7% (range 2.6–53.8%) out of 873 patients and the estimated pooled rate would be 30% (95% CI 20–41%, Fig. 1). There was significant heterogeneity in both effect sizes (Q-test X^2^ = 114.0, P < 0.0001; I^2^ = 93.0% and Q-test X^2^ = 112.7, P < 0.0001; I^2^ = 92.9%).

### 7-day SeHCAT retention < 15% (mild, moderate and severe BAD)

Seven studies reported prevalence using a 7-day cut-off value of < 15%. If the Rome III criteria were used in the paper by Shiha et al., the crude pooled rate would be 34.5% (range 23.7–84.6%) out of 597 patients and the estimated pooled rate would be 41% (95% CI 29–54%, Supplementary Fig. 2) by the random effects model. If the Rome IV criteria were used instead, the crude pooled rate would be 34.8% (range 2.6–53.8%) out of 563 patients and the estimated pooled rate would be 42% (95% CI 29–54%, Fig. 1). There was significant heterogeneity in both effect sizes (Q-test X^2^ = 63.2, P < 0.0001; I^2^ = 90.5% and Q-test X^2^ = 63.5, P < 0.0001; I^2^ = 90.6%).

### Prevalence of elevated total 48-h faecal bile acid level in patients fulfilling IBS criteria

Four studies including 1077 patients used an elevated level of total faecal bile acids over 48 h to make a diagnosis of BAD (Supplementary Table 7). In three studies, an elevated faecal bile acid concentration > 2337 μmol per 48 h was considered diagnostic. The remaining study used 2,619 μmol per 48 h. The crude pooled rate was 10.6% (range 7.4–35.2%). The estimated pooled rate was 25% (95% CI 8–43%, Supplementary Fig. 5) by the random effects model. There was significant heterogeneity in effect sizes (Q-test X^2^ = 38.2, P < 0.0001; I^2^ = 92.2%).

### Prevalence of elevated serum 7α-C4 in patients fulfilling IBS criteria

All four studies including 232 patients used different cut-off values of 7α-C4 to make a diagnosis (Supplementary Table 8). The crude pooled rate was 22.4% (range 13.3–33.3%). The estimated pooled rate was 22% (95% CI 16–27%, Supplementary Fig. 6) by the random effects model, with no heterogeneity in effect sizes (Q-test X^2^ = 3.0, P = 0.4; I^2^ = 0.0%). However, Dior et al. mentioned that none of their IBS patients would have had BAD if 47.1 ng/ml was chosen as the cut-off value, the level used by Camilleri et al. in their study. This would have resulted a crude pooled rate of 20.3% (range 0–24.1%) and an estimated pooled rate of 16% (95% CI 7–26%, Q-test X^2^ = 9.0, P = 0.03; I^2^ = 66.5%).

### Carbohydrate malabsorption (CM)

875 citations were identified. Of these, 97 appeared to be potentially relevant and the full texts of these citations were retrieved for full evaluation. 39 were included and 58 papers did not satisfy the eligibility criteria (Supplementary Fig. 7).

The carbohydrates studied included lactose, fructose, sorbitol and mannitol. Using hydrogen breath testing, 28 studies evaluated lactose malabsorption and/or intolerance; 12 examined fructose malabsorption and/or intolerance and six examined alterative or mixed forms of CM. Five groups utilised genotyping studies to identify lactase deficiency. Several of these studies reported prevalence of more than one form of CM.

### Prevalence of lactose malabsorption in patients fulfilling IBS criteria

**Figure 2:**
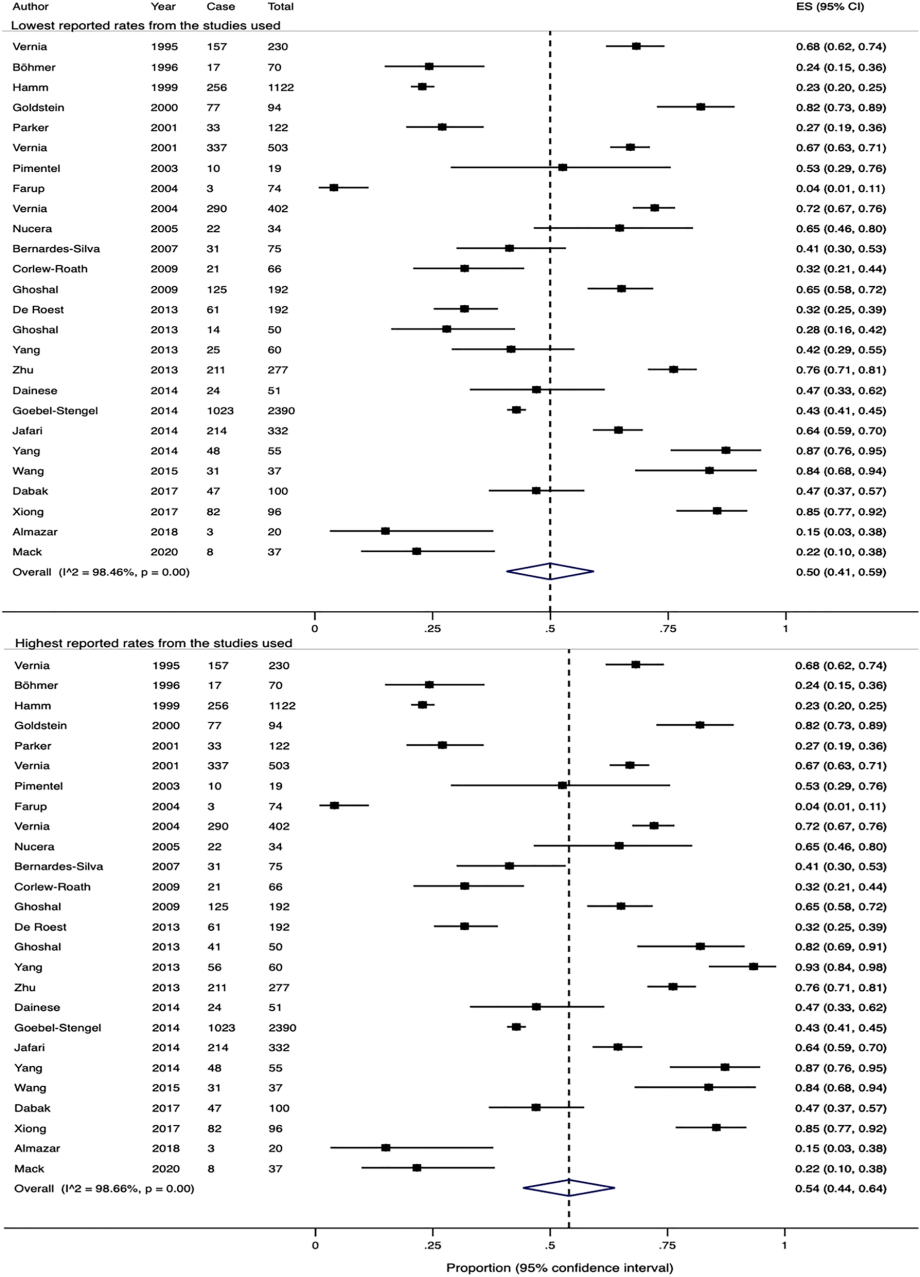
Forest plots showing the estimated pooled prevalence of lactose malabsorption using breath testing.

The 26 studies, including 6700 patients, reported the prevalence (Supplementary Table 9). Doses of lactose used in two studies^39,40^ were different, which resulted in a high variability in the rates of lactose malabsorption. The lowest and highest reported rates from these two studies gave respective crude pooled rates of 47.3% (range 4.1–87.3%) and 48.2% (range 4.1–93.3%) over the 26 studies. Similarly, the estimate pooled rates were 50% (95% CI 41–59%) and 54% (95% CI 44–64%), respectively (Fig. 2). There was significant heterogeneity in both effect sizes (Q-test X^2^ = 1627.3, P < 0.0001; I^2^ = 98.5% and Q-test X^2^ = 1871.6, P < 0.0001; I^2^ = 98.7%).

In addition to lactose malabsorption, nine of these 26 studies also evaluated the prevalence of lactose intolerance, which is generally defined as a positive breath test along with increased abdominal symptoms after ingestion of lactose. However, two other studies^41,42^ interpreted a positive breath test as lactose intolerance, even in the absence of abdominal symptoms. One of these 11 studies used different dosages of lactose for breath testing^40^. 3303 patients with IBS were assessed in total. The lowest and highest crude pooled rates from these 11 studies were 38% (range 18.3–71.7%) and 39.2% (range 21.0–85.0%), respectively. The estimated pooled rates were 40% (95% CI 31–49%) and 46% (95% CI 35–57%), with significant heterogeneity in both effect sizes (Q-test X^2^ = 146.3, P < 0.0001; I^2^ = 93.2% and Q-test X^2^ = 231.1, P < 0.0001; I^2^ = 95.7%, Supplementary Fig. 11).

Five studies including 970 patients applied genotyping studies to identify the homozygous state of a genetic variant, C/C-13910, for adult-type hypolactasia, also known as lactose intolerance (Supplementary Table 10). Two of these studies also evaluated another genetic variant, G/G-22018, which has also been shown to be associated with lactase deficiency, leading to lactose intolerance. The crude pooled rate for lactose intolerance as a result of the C/C-13910 gene was 37.9% (range 15.1–100.0%). The estimated pooled rate was 62% (95% CI 27–91%, Supplementary Fig. 11) by the random effects model, with significant heterogeneity in the effect sizes (Q-test X^2^ = 412.2, P < 0.0001; I^2^ = 99.0%).

The crude rate of lactose intolerance with the G/G-22018 gene in 225 patients from the two studies was 60.4% (range 47.2–68.0%).

### Prevalence of fructose malabsorption in patients fulfilling IBS criteria

**Figure 3:**
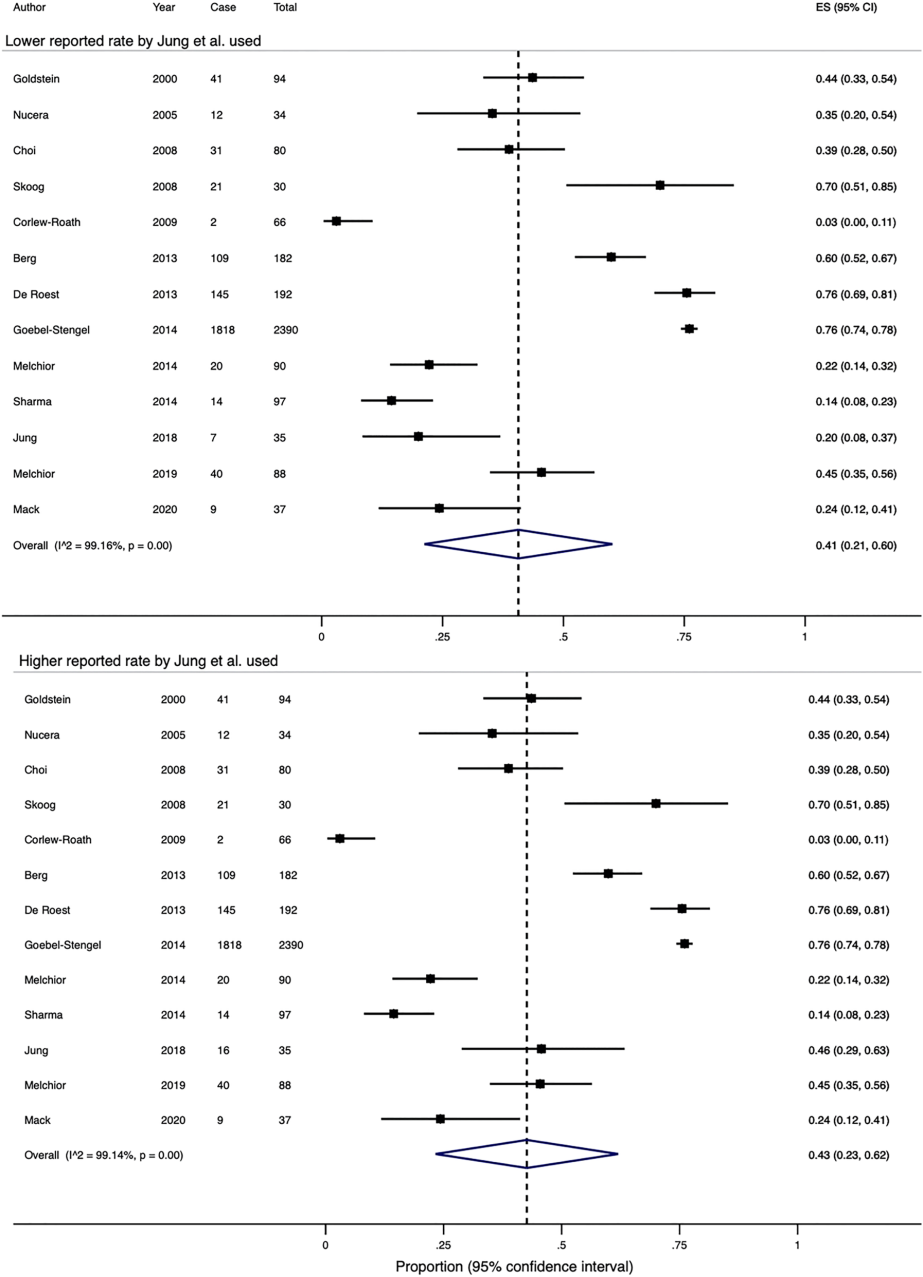
Forest plots showing the estimated pooled prevalence of fructose malabsorption using breath testing.

13 studies evaluated the prevalence of fructose malabsorption, including 3415 patients, using breath testing (Supplementary Table 11). One of the studies^43^ used two different dosages of fructose for breath testing, resulting in a difference in the reported rates. The lower and higher crude pooled rates from all 13 studies were 66.4% and 66.7%, respectively (range 3–76.1%). Using random effects models, the estimated pooled rates were 41% (95% CI 21–60%) and 43% (95% CI 23–62%), respectively (Fig. 3). Significant heterogeneity was noted in both effect sizes (Q-test X^2^ = 1434.2, P < 0.0001; I^2^ = 99.2% and Q-test X^2^ = 1401.4, P < 0.0001; I^2^ = 99.1%).

Four of these 12 studies, including 2590 patients, also examined the prevalence of fructose intolerance, in addition to fructose malabsorption. The crude pooled rate was 60.9% (range 7.8–64.1%). Using the random effect model, the estimated pooled rate was 37% (95% CI 2–71%), with a significant heterogeneity in effect sizes (Q-test X^2^ = 376.3, P < 0.0001; I^2^ = 99.2%, Supplementary Fig. 16).

### Prevalence of alternative or mixed forms of CM in patients fulfilling IBS criteria

Six studies shown (Supplementary Table 12) reported the rates of alternative or mixed forms of CM.

Three studies investigated sorbitol malabsorption in a total of 91 patients and gave a crude rate of 47.3% (range 35.3–60.0%). The estimated prevalence was 48% (95% CI 34–62%), with homogeneity demonstrated between studies (Q-test X^2^ = 3.7, P = 0.15; I^2^ = 46.4%, Supplementary Fig. 17).

Two groups reported that 31.4% and 70.2% of their patients fulfilling IBS criteria had a positive breath test for combined fructose-sorbitol malabsorption. Another group concluded that 9.4% of patients had either lactose or combined fructose-sorbitol malabsorption. One of the groups also reported that 20% of patients also had mannitol malabsorption.

### Microscopic colitis (MC)

The search identified 1484 citations. Of these, 91 were potentially relevant and full texts of these citations were retrieved. 17 papers were included, 74 did not satisfy our eligibility criteria (Supplementary Fig. 18 and Table 13). Four of these 17 studies reported the prevalence of MC but did not specify the subtype, one study only reported patients with lymphocytic colitis. The remaining 12 studies reported the prevalence of both lymphocytic and collagenous colitis, defined by a confirmed histological diagnosis (> 15 or > 20 lymphocytes in 100 epithelial cells for lymphocytic colitis and > 10 μm or > 15 μm thickened sub-epithelial collagen band for collagenous colitis).

### Prevalence of MC in patients fulfilling IBS criteria

**Figure 4:**
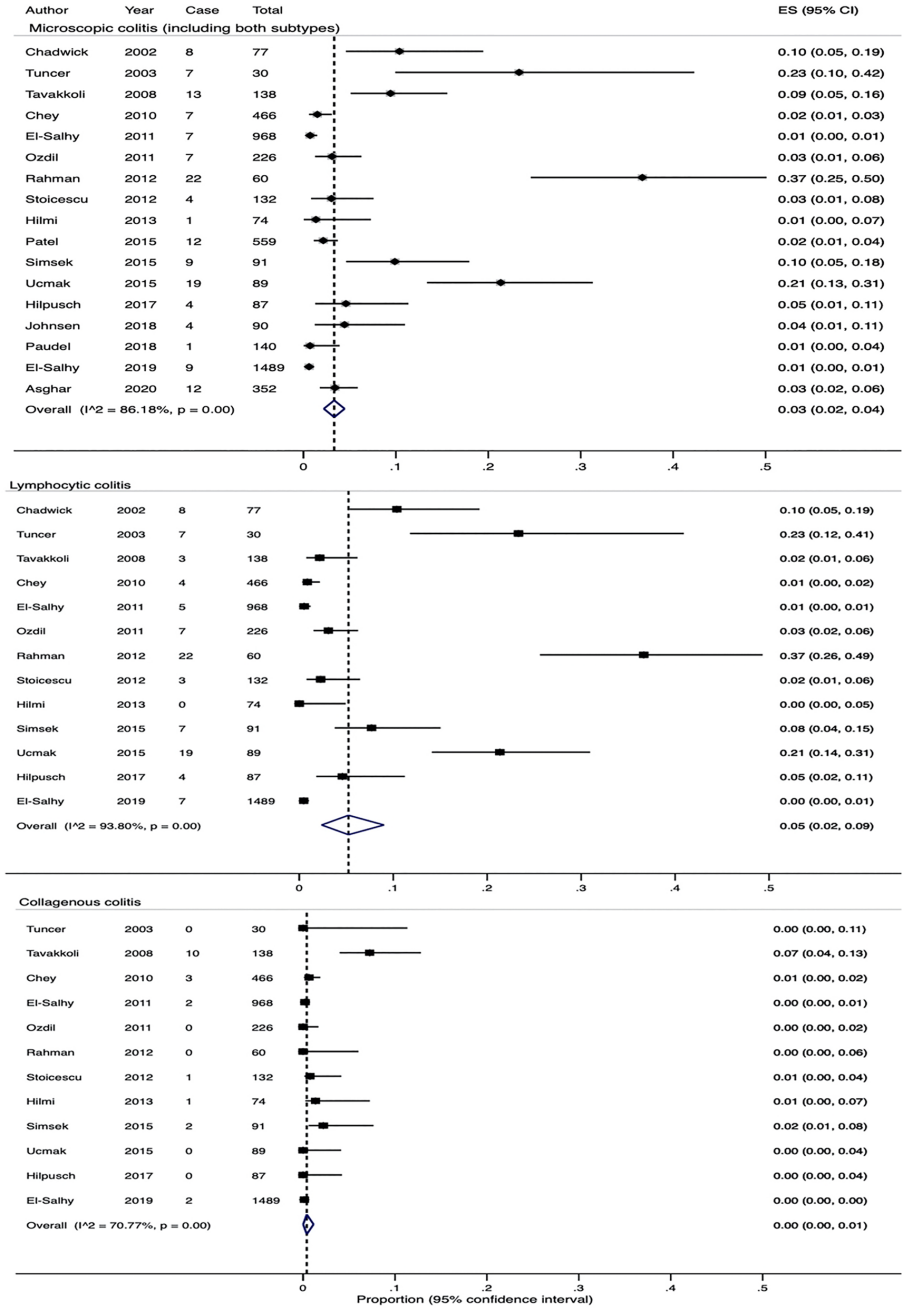
Forest plots showing the estimated pooled prevalence of MC and both subtypes—lymphocytic colitis and collagenous colitis.

5,068 patients were included. The overall crude pooled rate for MC, including both subtypes, lymphocytic and collagenous colitis, was 2.9% (range 0.6–36.7%). The overall estimated rate was 3% (95% CI 2–4%, Fig. 4). There was significant heterogeneity in effect sizes (Q-test X^2^ = 115.8, P < 0.0001; I^2^ = 86.2%). Three studies^44–46^ were deemed to be of high risk of bias and after excluding them a repeat analysis estimated the prevalence of MC to be 4% (95% CI 2–5%), and the level of heterogeneity between studies remained unchanged (Q-test X^2^ = 107.0, P < 0.0001; I^2^ = 87.9%, Supplementary Fig. 19).

Crude pooled rates from the studies were 2.4% out of 3,927 patients (range 0–36.7%) for lymphocytic colitis and 0.5% out of 3,850 patients (range 0–7.2%) for collagenous colitis; estimated rates were 5% (95% CI 2–9%) and 0% (95% CI 0–1%), respectively (Fig. 4). Significant heterogeneity between studies was noted in both groups (Q-test X^2^ = 193.4, P < 0.0001; I^2^ = 93.8% and Q-test X^2^ = 37.6, P = 0.02; I^2^ = 70.8%).

### Pancreatic exocrine insufficiency (PEI)

The search strategy identified 314 citations. Of these, 11 appeared to be potentially relevant and the full text of these citations were retrieved. Two papers were included, nine did not satisfy our eligibility criteria (Supplementary Fig. 24). Both papers utilised faecal elastase-1 to diagnose PEI.

### Prevalence of PEI in patients fulfilling IBS criteria

A meta-analysis was not performed due to the number of papers identified. The two papers gave a crude rate of 4.6% (22/478), ranging between 1.8 and 6.1% (Supplementary Table 14).

### Small intestinal bacteria overgrowth (SIBO)

The search strategy identified 1674 citations. Of these, 114 appeared to be potentially relevant and the full text of these citations were retrieved. 59 papers were included, 55 did not satisfy our eligibility criteria (Supplementary Fig. 25).

55 studies evaluated the prevalence of SIBO within their IBS patient cohorts utilising breath testing with glucose and lactulose. Three of these studies, alongside an additional four studies, examined small bowel fluid from their patients to identify those with SIBO (Supplementary Table 15).

### Prevalence of SIBO in patients fulfilling IBS criteria

**Figure 5:**
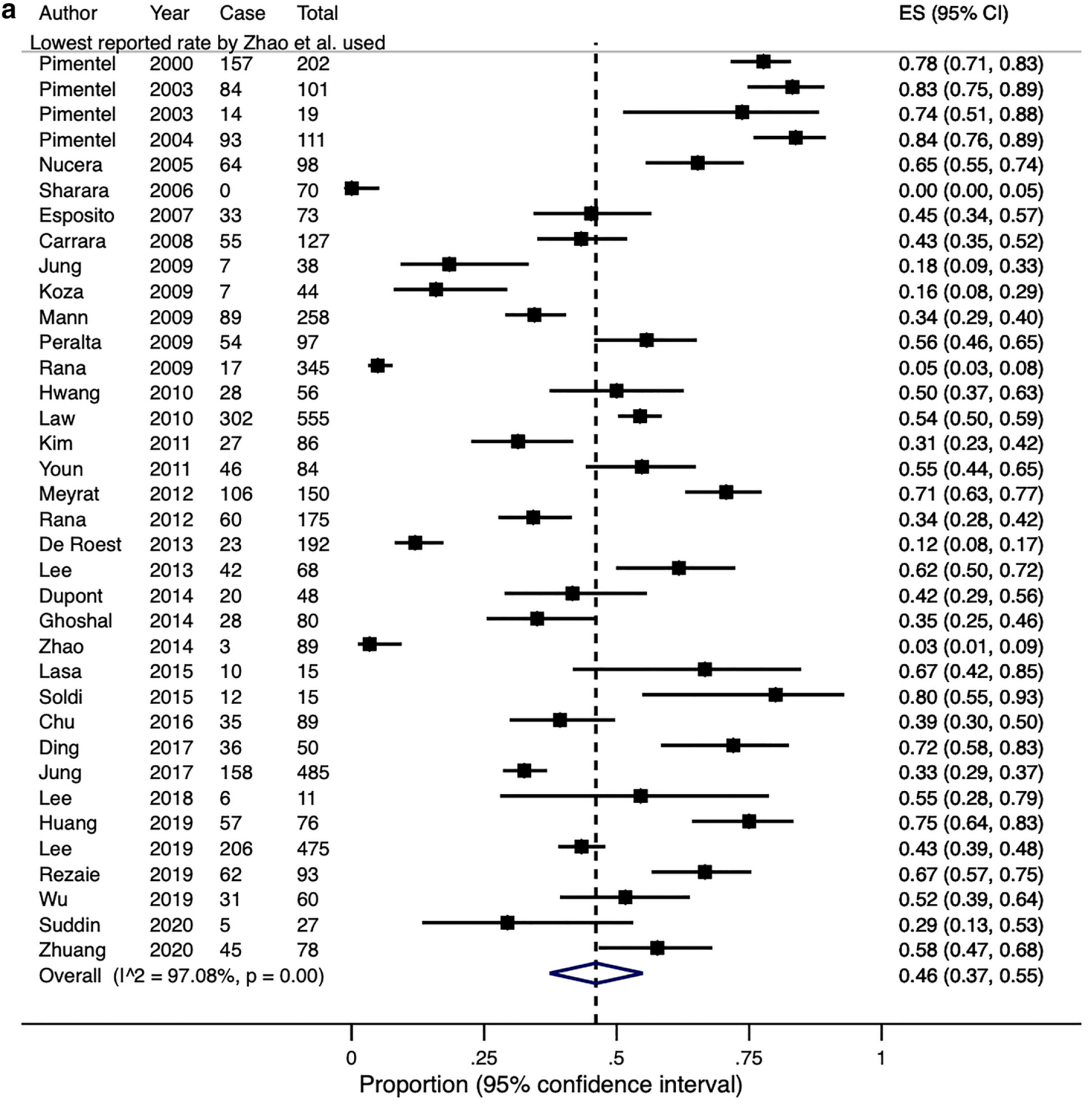

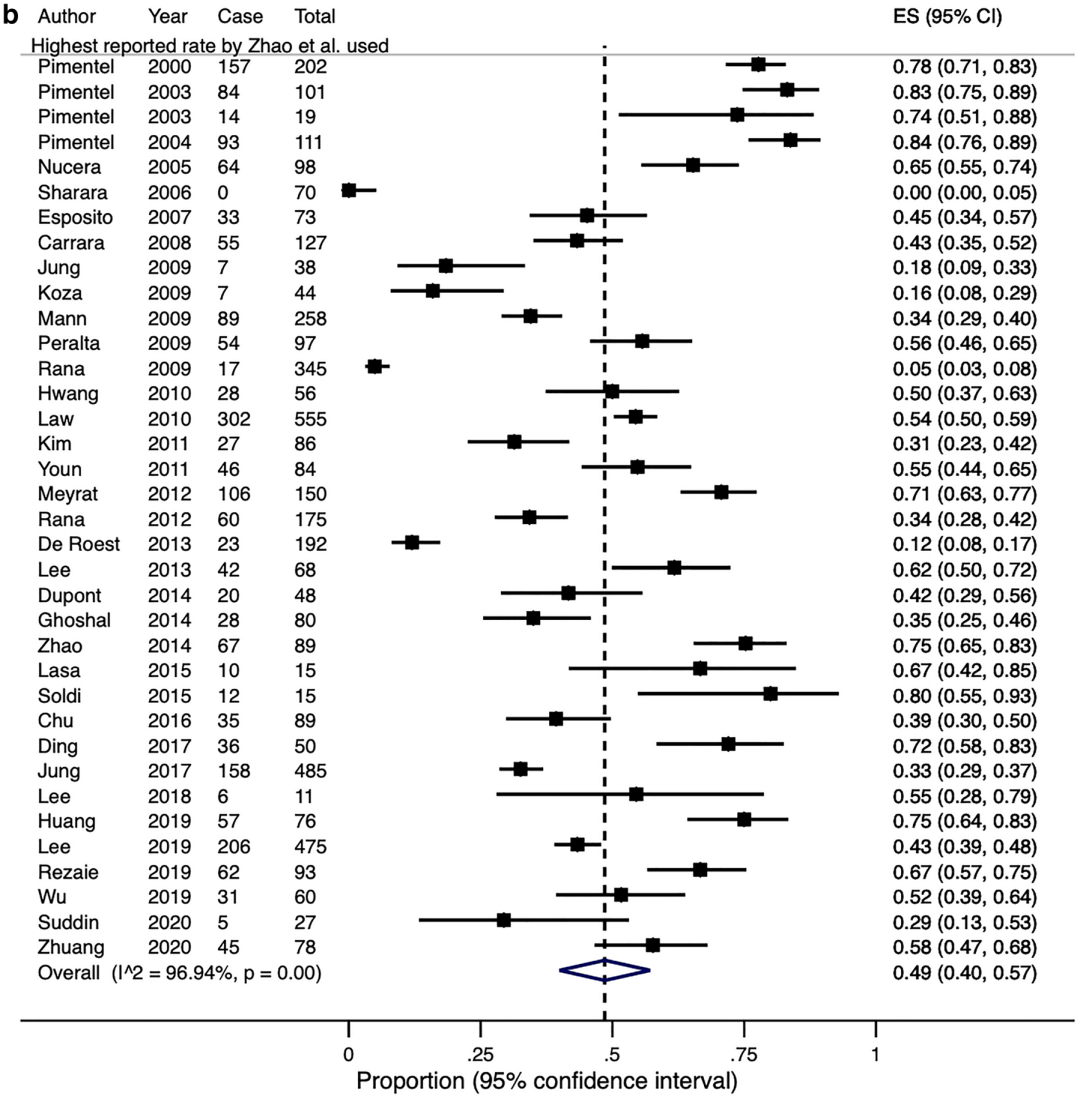
(**a**) A Forest plot showing the estimated pooled prevalence of SIBO, diagnosed with a positive lactulose breath test, using the lowest Zhao et al. paper. (**b**) A Forest plot showing the estimated pooled prevalence of SIBO, diagnosed with a positive lactulose breath test using the highest reported rates from Zhao et al. paper.

Lactulose was used in 36 studies including 4630 patients as the substrate for breath testing. One group compared and demonstrated a wide variation in the prevalence of SIBO by using six different diagnostic criteria to define their breath tests as positive^47^. If the lowest and highest reported rates from this study were adopted, the overall crude pooled rates for SIBO diagnosed by lactulose breath testing would be 43.7% and 45.1% (range 0–83.8%), respectively. Similarly, the estimated pooled rates would be 46% (95% CI 37–55%) and 49% (95% CI 40–57%), with significant heterogeneity in both effect sizes (Q-test X^2^ = 1199.3, P < 0.0001; I^2^ = 97.1% and Q-test X^2^ = 1142.5, P < 0.0001; I^2^ = 96.9%, Fig. 5).

**Figure 6:**
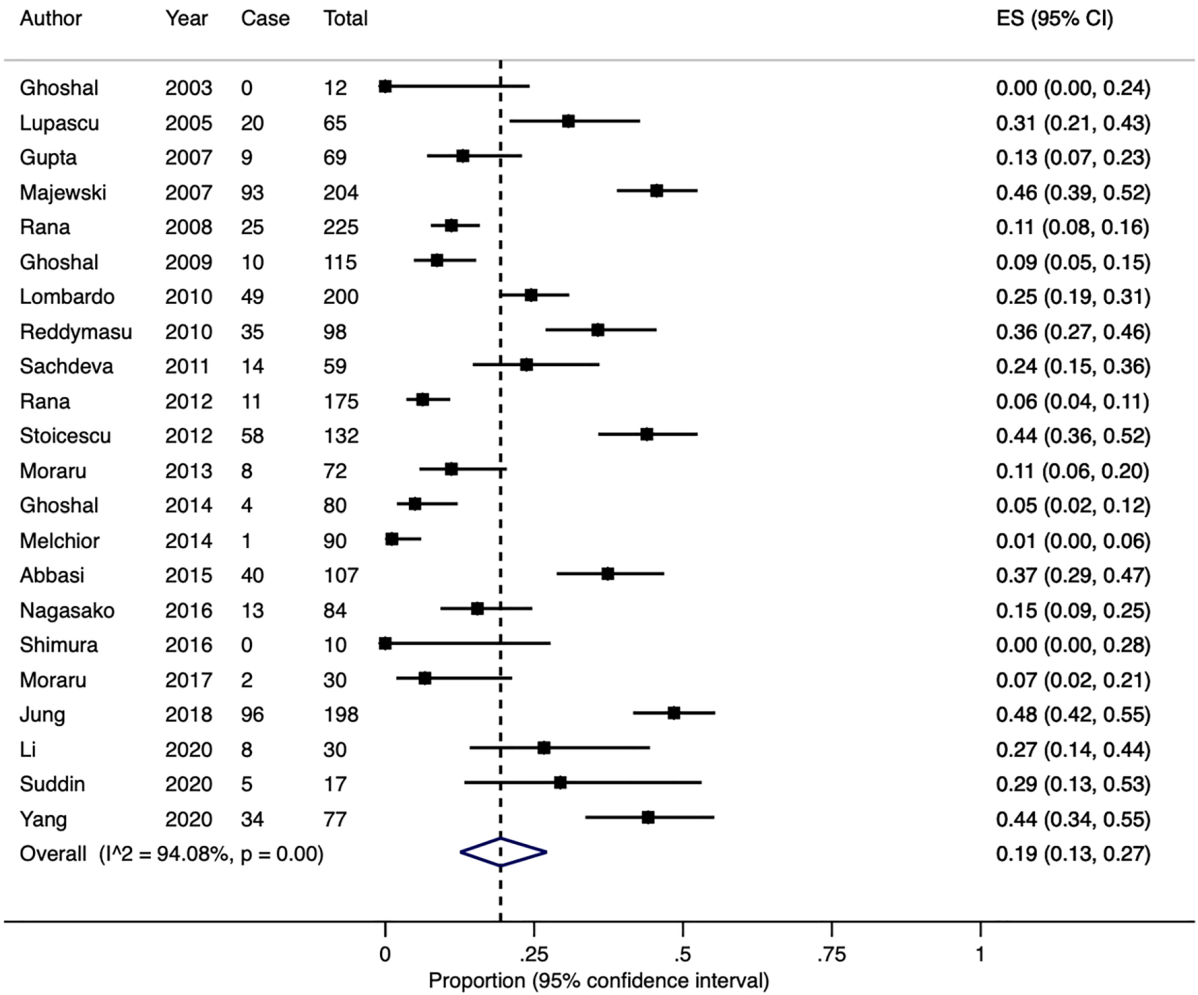
A Forest plot of the 22 studies showing the estimated pooled prevalence of SIBO, diagnosed with a positive glucose breath test.

Glucose was utilised as the substrate for breath testing in 22 studies including 2149 patients. The crude pooled rate was 24.9% (range 0–48.5%) and the estimated pooled rate was 19% (95% CI 13–27%, Fig. 6). There was significant heterogeneity in the effect sizes (Q-test X^2^ = 354.9, P < 0.0001; I^2^ = 94.1%).

Seven studies examined 608 patients’ small bowel fluid to diagnose SIBO. The overall crude pooled rate was 14.5% (range: 4.2–37.5%) and the estimated pooled rate was 13% (95% CI 4–25%, Supplementary Fig. 32). There was significant heterogeneity in the effect sizes (Q-test X^2^ = 77.3, P < 0.0001; I^2^ = 92.2%).

## Discussion

This systematic review brings together data on multiple conditions other than coeliac disease, inflammatory bowel disease or colorectal cancer that may be present in adults fulfilling clinical criteria for IBS. These data show a high frequency of treatable gastrointestinal diseases in this patient group. Approximately 40% had BAD; CM was often detected—54% with lactose malabsorption and 44% with fructose malabsorption; 5% had PEI; 3% had MC; and 13–49% had SIBO. Importantly, pooled data from the studies which used healthy controls, the rates of BAD, lactose and fructose malabsorption, MC and SIBO were also significantly higher in patients fulfilling IBS criteria (Supplementary Tables 16–24). We also found similar diagnostic yields for BAD in studies using total faecal bile acids and fasting serum 7*α*-C4.

Some may argue that abnormal test results do not correlate perfectly with treatment responses, so the clinical relevance of such results is not defined. Although this study did not assess treatment responses to these possible diagnoses, our findings are consistent with the substantial published data which report at least good short term symptom response rates in large numbers of patients when appropriate treatment is given once these diagnoses are made^34,48–63^. However, more studies are required to assess whether prediction of long-term treatment response can be improved.

Although our data show that both lactose and fructose malabsorption were significantly more prevalent in IBS patients than in healthy individuals, it is worth noting that CM may be physiological (Supplementary Tables 17 and 24). It is often the sensory response following ingestion of carbohydrates—intolerance, that is pathological. For lactose intolerance, similar observation to lactose malabsorption can be made among the IBS patients and healthy controls (Supplementary Tables 17 and 24). Reduction of exposure to such carbohydrates may be therapeutic and should be considered in managing CM^64–66^.

Our estimated rate for MC is slightly lower than 9.8% reported previously in patients with diarrhoea-predominant IBS and functional diarrhoea^67^. This could be explained by the fact that we have included studies that selected patients with symptoms fulfilling diagnostic criteria for IBS and excluded studies recruiting subjects with functional diarrhoea. Also, patients with constipation predominant IBS were included in some of these studies. Two of the studies we included only examined biopsies from the left side of colon^45,68^ which may also underestimate prevalence rates. Our calculated crude pooled rate may also underestimate PEI as Leeds et al*.* used a faecal elastase-1 cut-off of < 100 μg/g, reflecting only the presence of severe PEI^54^. However, stool samples provided by individuals suffering from longstanding diarrhoea are often soft or liquid and can result in a falsely low faecal elastase-1 level, leading to PEI being over-diagnosed in this cohort and therefore, our calculated rate of PEI should be interpreted with caution.

Glucose hydrogen breath testing and small bowel aspirate for SIBO give relatively comparable yields. However, the prevalence rate of SIBO using small bowel aspirate is likely to be over-estimated as one study^69^ used a low bacterial count > 10^3^ cfu/mL to diagnose SIBO, which has been shown to result in a twofold increase in diagnosis^70^. Interestingly, the two studies, both by Ghoshal et al.^71,72^ comparing the two diagnostic tests reported very different rates for SIBO. The small sample size used in the earlier study was likely the cause of such results. It is not as clear why in some studies the rates of SIBO using glucose breath testing were much lower than most of the other studies, but one of the possible explanations was that a more stringent criteria was used to define SIBO.

It is worth noting that despite hydrogen breath test being a well-established diagnostic test for SIBO and performed worldwide, standardisation regarding test methodology and interpretation of results is lacking and opinions among healthcare professionals are widely divided. Glucose and lactulose are the two recommended substrates when performing a hydrogen breath test to look for SIBO. Although 75 g of glucose and 10 g of lactulose are used in most studies, various doses (glucose: 50 g, 75 g, 100 g; lactulose: 6.7 g, 10 g) have been used (Supplementary Table 15). The length of breath test also greatly varies in the literature, ranging from 2 to 5 h^73^. Opinions on how HBT should be interpreted are also widely divided. Most centres use a rise of ≥ 20 parts per million (ppm) in H_2_ above baseline by 90 min to define the HBT as positive^73^, but some consider a rise within 60 min as positive^74^. As lactulose can shorten the intestinal transit time and reach the proximal colon within 90 min, the former approach could potentially lead to an increase in false positive result. In contrast, the latter approach is more conservative but could lead to false negative results.

Most systematic reviews restricted their study inclusion to one type of study design (i.e. case–control studies) and those with a large sample size (i.e., > 100 patients). We have included studies of all types and sample sizes recruiting consecutive patients to minimise potential selection bias^75^. We included only studies that investigated patients with IBS defined by strict diagnostic criteria, and using recognised diagnostic tests for an alternative disorder.

There are several limitations to this study. First, the inclusion of smaller studies may have biased our estimated rates, particularly in conditions where only a small number of studies were included in the overall analyses. Modestly different rates can be seen after excluding studies with a sample size < 100. Such disparity is not as evident in conditions with a greater number of large studies. Also, the lack of an appraisal tool that accurately assesses all the types of study included limits confident identification and reporting of bias. The exclusion of non-English publications and those in only abstract form might have introduced bias; however, in comparison to the number of literatures included in this review, the number of these excluded publications was very small so they are unlikely to affect our estimates.

Secondly, substantial statistical heterogeneity was present in all of the pooled estimates and usually remained following subgroup analysis. Significant clinical heterogeneity among studies including difference in sample size, diagnostic criteria, diagnostic tests used and interpretation of their results could all have contributed. It is important to point out that whilst some studies examined only patients with diarrhoea predominant IBS, others included those with constipation predominant and mixed type of IBS and this can impact on the reported rates. It is also clear that the accuracy of some tests used to diagnose these conditions is suboptimal (Supplementary Table 25), and a better diagnostic test is urgently needed to inform us a more accurate diagnosis of CM and SIBO. Further work is needed to characterise test performance and how it relates to treatment response.

Thirdly, while pooled rates for the five conditions were calculated, we did not compare them with those in controls to calculate odds ratios. However, these conditions have already been shown to be more prevalent in patients diagnosed with IBS^34,54,65,76–78^ which is consistent with our findings. We also looked at the prevalence of other rare forms of carbohydrate malabsorption including sorbitol, mannitol and fructose-sorbitol malabsorption but fewer than three papers were identified for each of these conditions so we did not discuss about them in much detail. We did not search systematically for congenital sucrase-isomaltase deficiency, a very rare condition which can cause IBS-type symptoms, as we believe the search would unlikely yield enough studies for any meaningful analysis. Lastly, all of the included studies were conducted in a secondary or tertiary healthcare settings so our data here may not be relevant to primary care.

Current management strategies for patients with IBS-like symptoms work poorly with up to 50% of patients remaining significantly symptomatic 6 years after diagnosis^79^. This systematic review strongly suggests that one of the reasons for this is may be because organic disorders, which do not respond to conventional treatments for IBS, will be missed if patients are not investigated adequately. However, our findings are limited by the significant clinical and statistical heterogeneity between selected studies and the shortcomings of current diagnostic tests, in particular breath testing for CM and SIBO, and hence they should be interpretated with a degree of caution. Appropriate studies measuring long term outcomes and the benefits for patients, society and healthcare from accurate diagnosis and optimal treatment are urgently needed to examine the true significance of these organic conditions in patients with IBS-like symptoms.

## Conclusion

Our study has shown that treatable GI conditions can be found in a proportion of patients with IBS-like symptoms referred to secondary care. Specialist clinicians should be aware of such conditions and further tests should be considered in patients who do not respond to conventional treatments for IBS. Further research is needed to evaluate the clinical and cost-effectiveness of such an approach compared to symptom-based management alone.

## Supplementary Information

Supplementary Information.

## Abbreviations

BAD: Bile acid diarrhoea
CASP: Critical Appraisal Skills Programme
cfu: Colony-forming units
CI: Confidence interval
CM: Carbohydrate malabsorption
GI: Gastrointestinal
IBS: Irritable bowel syndrome
MC: Microscopic colitis
MeSH: Medical subject heading
PEI: Pancreatic exocrine insufficiency
SeHCAT: ^75^Selenium homocholic acid taurine
SIBO: Small intestinal bacterial overgrowth
7α-C4: 7α-Hydroxy-4-cholesten-3-one
