# Supplementary material for: A systematic review and meta-analysis on the prevalence of non-malignant, organic gastrointestinal disorders misdiagnosed as irritable bowel syndrome
Source: Sci Rep. 2022 Feb 4;12:1949. doi: 10.1038/s41598-022-05933-1 (PMC8817019; doi:10.1038/s41598-022-05933-1)
Supplement: Supplementary file 1 — Supplementary Information. [file 41598_2022_5933_MOESM1_ESM.docx]

Supplementary Information

*Scientific Reports*

A systematic review and meta-analysis on the prevalence of non-malignant, organic gastrointestinal disorders misdiagnosed as irritable bowel syndrome

Dennis Poon^1^, Graham R. Law^2^, Giles Major^3^, H. Jervoise N. Andreyev^3^

1. Department of Gastroenterology, Lincoln County Hospital, United Kingdom

2. Community and Health Research Unit, School of Health and Social Care, University of Lincoln, United Kingdom

3. NIHR Nottingham Biomedical Research Centre, Nottingham University Hospitals NHS Trust and the University of Nottingham, United Kingdom

Correspondence to

Professor Jervoise Andreyev, Department of Gastroenterology, Lincoln County Hospital, Greetwell Road, Lincoln, LN2 5QY, United Kingdom

Email: [jervoiseandreyev@gmail.com](mailto:jervoiseandreyev@gmail.com)

| **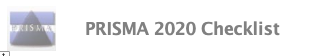Section and Topic** | **Item #** | **Checklist item** | **Location where item is reported** |
| --- | --- | --- | --- |
| **TITLE** | | |  |
| Title | 1 | Identify the report as a systematic review. | Page 1 |
| **ABSTRACT** | | |  |
| Abstract | 2 | See the PRISMA 2020 for Abstracts checklist. | Page 3 |
| **INTRODUCTION** | | |  |
| Rationale | 3 | Describe the rationale for the review in the context of existing knowledge. | Page 7-8 |
| Objectives | 4 | Provide an explicit statement of the objective(s) or question(s) the review addresses. | Page 7-8 |
| **METHODS** | | |  |
| Eligibility criteria | 5 | Specify the inclusion and exclusion criteria for the review and how studies were grouped for the syntheses. | Page 9-10 |
| Information sources | 6 | Specify all databases, registers, websites, organisations, reference lists and other sources searched or consulted to identify studies. Specify the date when each source was last searched or consulted. | Page 9-10 |
| Search strategy | 7 | Present the full search strategies for all databases, registers and websites, including any filters and limits used. | Page 9-10 |
| Selection process | 8 | Specify the methods used to decide whether a study met the inclusion criteria of the review, including how many reviewers screened each record and each report retrieved, whether they worked independently, and if applicable, details of automation tools used in the process. | Page 9-10 |
| Data collection process | 9 | Specify the methods used to collect data from reports, including how many reviewers collected data from each report, whether they worked independently, any processes for obtaining or confirming data from study investigators, and if applicable, details of automation tools used in the process. | Page 9-13 |
| Data items | 10a | List and define all outcomes for which data were sought. Specify whether all results that were compatible with each outcome domain in each study were sought (e.g. for all measures, time points, analyses), and if not, the methods used to decide which results to collect. | Page 9-13 |
|  | 10b | List and define all other variables for which data were sought (e.g. participant and intervention characteristics, funding sources). Describe any assumptions made about any missing or unclear information. | Page 9-13 |
| Study risk of bias assessment | 11 | Specify the methods used to assess risk of bias in the included studies, including details of the tool(s) used, how many reviewers assessed each study and whether they worked independently, and if applicable, details of automation tools used in the process. | Page 11 |
| Effect measures | 12 | Specify for each outcome the effect measure(s) (e.g. risk ratio, mean difference) used in the synthesis or presentation of results. | Page 12-13 |
| Synthesis methods | 13a | Describe the processes used to decide which studies were eligible for each synthesis (e.g. tabulating the study intervention characteristics and comparing against the planned groups for each synthesis (item #5)). | Page 9-13 |
|  | 13b | Describe any methods required to prepare the data for presentation or synthesis, such as handling of missing summary statistics, or data conversions. | Page 9-13 |
|  | 13c | Describe any methods used to tabulate or visually display results of individual studies and syntheses. | Page 9-13 |
|  | 13d | Describe any methods used to synthesize results and provide a rationale for the choice(s). If meta-analysis was performed, describe the model(s), method(s) to identify the presence and extent of statistical heterogeneity, and software package(s) used. | Page 13 |
|  | 13e | Describe any methods used to explore possible causes of heterogeneity among study results (e.g. subgroup analysis, meta-regression). | Page 12-13 |
|  | 13f | Describe any sensitivity analyses conducted to assess robustness of the synthesized results. | Page 9-13 |
| Reporting bias assessment | 14 | Describe any methods used to assess risk of bias due to missing results in a synthesis (arising from reporting biases). | Page 11 |
| Certainty assessment | 15 | Describe any methods used to assess certainty (or confidence) in the body of evidence for an outcome. | Page 13 |
| **RESULTS** | | |  |
| Study selection | 16a | Describe the results of the search and selection process, from the number of records identified in the search to the number of studies included in the review, ideally using a flow diagram. | Page 10 |
|  | 16b | Cite studies that might appear to meet the inclusion criteria, but which were excluded, and explain why they were excluded. | Page 10 |
| Study characteristics | 17 | Cite each included study and present its characteristics. | Supplementary material |
| Risk of bias in studies | 18 | Present assessments of risk of bias for each included study. | Main manuscript + Supplementary material |
| Results of individual studies | 19 | For all outcomes, present, for each study: (a) summary statistics for each group (where appropriate) and (b) an effect estimate and its precision (e.g. confidence/credible interval), ideally using structured tables or plots. | Main manuscript + Supplementary material |
| Results of syntheses | 20a | For each synthesis, briefly summarise the characteristics and risk of bias among contributing studies. | Page 14-23 |
|  | 20b | Present results of all statistical syntheses conducted. If meta-analysis was done, present for each the summary estimate and its precision (e.g. confidence/credible interval) and measures of statistical heterogeneity. If comparing groups, describe the direction of the effect. | Main manuscript + Supplementary material |
|  | 20c | Present results of all investigations of possible causes of heterogeneity among study results. | Page 14-23 |
|  | 20d | Present results of all sensitivity analyses conducted to assess the robustness of the synthesized results. | Not reported |
| Reporting biases | 21 | Present assessments of risk of bias due to missing results (arising from reporting biases) for each synthesis assessed. | Supplementary material |
| Certainty of evidence | 22 | Present assessments of certainty (or confidence) in the body of evidence for each outcome assessed. | Main manuscript + Supplementary material |
| **DISCUSSION** | | |  |
| Discussion | 23a | Provide a general interpretation of the results in the context of other evidence. | Page 24-27 |
|  | 23b | Discuss any limitations of the evidence included in the review. | Page 24-27 |
|  | 23c | Discuss any limitations of the review processes used. | Page 24-27 |
|  | 23d | Discuss implications of the results for practice, policy, and future research. | Page 24-27 |
| **OTHER INFORMATION** | | |  |
| Registration and protocol | 24a | Provide registration information for the review, including register name and registration number, or state that the review was not registered. | Page 9 |
|  | 24b | Indicate where the review protocol can be accessed, or state that a protocol was not prepared. | Page 9 |
|  | 24c | Describe and explain any amendments to information provided at registration or in the protocol. | Page 9 |
| Support | 25 | Describe sources of financial or non-financial support for the review, and the role of the funders or sponsors in the review. | Page 2 |
| Competing interests | 26 | Declare any competing interests of review authors. | Page 2 |
| Availability of data, code and other materials | 27 | Report which of the following are publicly available and where they can be found: template data collection forms; data extracted from included studies; data used for all analyses; analytic code; any other materials used in the review. | Not reported |

**
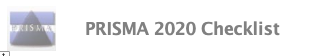
**

*From:*  Page MJ, McKenzie JE, Bossuyt PM, Boutron I, Hoffmann TC, Mulrow CD, et al. The PRISMA 2020 statement: an updated guideline for reporting systematic reviews. BMJ 2021;372:n71. doi: 10.1136/bmj.n71

For more information, visit: <http://www.prisma-statement.org/>

**Subgroup analysis and repeat analysis after exclusion of papers of high risk of bias**

**Bile acid diarrhoea (BAD)**

#### **7-day SeHCAT retention <5% (severe BAD)**

Subgroup analysis performed on large studies revealed homogeneity but significant heterogeneity remained in analyses with both study design and diagnostic criteria used for IBS (Figure 3).

One study [1] was deemed to be of high risk of bias and a repeat analysis was performed after excluding this study which led to homogeneity between studies (Q-test X^2^ =5.5, P =0·24; I^2^ =27·1%) with an estimated prevalence of 8% (95% CI 6-11%, Figure 4).

#### **7-day SeHCAT retention <10% (moderate and severe BAD)**

Subgroup analyses demonstrated that the differences in samples sizes of the studies, study design and diagnostic criteria for IBS used were all contributing to this heterogeneity (Figure 3).

One study [1] was deemed to be of high risk of bias and a repeat analysis was performed after excluding this study. Significant heterogeneity remained (Q-test X^2^ =83·4, P <0·0001; I^2^ =91·6%) with an estimated prevalence of 26% (95% CI 16-37%, Figure 4).

#### **7-day SeHCAT retention <15% (mild, moderate and severe BAD)**

Subgroup analyses demonstrated that the differences in samples sizes of the studies, study design and diagnostic criteria for IBS used were all contributing to this heterogeneity (Figure 3).

One study [1] was deemed to be of high risk of bias and a repeat analysis was performed after excluding this study. This resulted in a significant reduction in the heterogeneity between studies (Q-test X^2^ =10·6, P =0·06; I^2^ =52·8%) with an estimated prevalence of 32% (95% CI 27-39%, Figure 4).

**Carbohydrate malabsorption (CM)**

**Lactose malabsorption**

Doses of lactose used in two studies were different [2, 3]. Using the lowest reported rates from these two studies, subgroup analyses were performed using the methodology described above and additionally allowing for the location of study conducted (Asia, Europe and North America) and the dose of lactose for breath testing (25g and 50g). Significant heterogeneity was noted across all subgroup analyses (Figure 8). We did not repeat the same analyses using the highest prevalence reported from the 2 studies as we believed it would generate similar results. Inspection of funnel plot indicated substantial small-study effects or publication bias (Figure 9).

Four studies [4-7] that examined lactose malabsorption were deemed to be at high risk of bias. After excluding the studies and using the lowest reported rates from the aforementioned two studies, a repeat analysis showed an estimated pooled rate to be 53% (95% CI 43-63%, Figure 10) with the same level of heterogeneity between studies (Q-test X^2^ =1,542·3, P <0·0001; I^2^ =98·6%).

One the five studies [6] of which applied genotyping studies to identify lactose intolerance was deemed to be of high risk of bias and a repeat analysis following the exclusion of this study estimated the prevalence to be 74% (95% CI 48-93%, Figure 12). The heterogeneity between studies was reduced but remained significant (Q-test X^2^ =86·1, P <0·0001; I^2^ =96·5%).

**Fructose malabsorption**

Subgroup analyses were performed and included the dosage of fructose for breath testing (25g), and study location (Europe and North America). Significant heterogeneity was noted across all subgroup analyses (Figure 13). These analyses were conducted using the lower prevalence reported by Jung and colleagues [8]. Repeat analyses using the higher prevalence were not performed as the results would likely be very similar. Inspection of funnel plots indicated substantial small-study effects or publication bias (Figure 14).

Four studies [4, 7, 9, 10] that examined fructose malabsorption were deemed to be at high risk of bias. A repeat analysis after their exclusion, using the lowest reported rate from the Jung et al., a repeat analysis showed an estimated pooled rate to be 48% (95% CI 32-64%) with the same level of heterogeneity between studies (Q-test X^2^ =322·5, P <0·0001; I^2^ =97.5%, Figure 15).

**Microscopic colitis (MC)**

For both subtypes of MC, we performed subgroup analyses separately and included study location (Asia and Europe) and the number of lymphocytes in 100 epithelial cells for lymphocytic colitis or thickness of sub-epithelial collagen band for collagenous colitis.

**Lymphocytic colitis**

Significant heterogeneity remained in all subgroup analyses (Figure 20). Some small-study effect or publication bias was present by inspecting the funnel plot (Figure 22).

**Collagenous colitis**

Homogeneity was observed in subgroup analyses of studies in which Rome III was utilised as the diagnostic criteria for IBS and those conducted in Europe (Figure 21). Similar level of heterogeneity remained in subgroup analyses of prospective studies, studies conducted in Asia and those identified the condition in the presence of sub-epithelial collagen band of >10 μm thickness, when compared to overall analysis of all studies. Funnel plot indicated no evidence of small-study effect or publication bias (Figure 23).

**Small intestinal bacterial overgrowth (SIBO)**

**Diagnosis made using lactulose hydrogen breath test**

One group compared and demonstrated a wide variation in the prevalence of SIBO by using six different diagnostic criteria to define their breath tests as positive [11].

Subgroup analyses were performed and included additionally the dose of lactulose for breath testing (10g), and the study location (Asia, Europe and North America). This demonstrated a similar level of heterogeneity between studies to that of the overall analysis (Figure 26). The lowest prevalence rate from the aforementioned study was used in such analyses and repeat analyses were not performed as we believed the results would not be much different. Inspection of funnel plot indicated substantial small-study effect or publication bias (Figure 27).

12 studies [12-23] were deemed to be at high risk of bias. After excluding these studies, a repeat analysis using the lowest reported rate from the aforementioned study estimated the prevalence of SIBO diagnosed with lactulose breath testing to be 42% (95% CI 31-53%), with same level of heterogeneity between studies (Q-test X^2^ =707·9, P <0·0001; I^2^ =97·3%, Figure 28).

**Diagnosis made using glucose hydrogen breath test**

Subgroup analyses were performed and included additionally the dose of glucose for breath testing (50g and 100g), and the study location (Asia and Europe). Similar level of heterogeneity remained in all subgroup analyses (Figure 29). Inspection of funnel plot indicated some small-study effect or publication bias (Figure 30).

Eight studies [24-31] were deemed to be of high risk of bias. After excluding these studies, a repeat analysis was performed and estimated the prevalence of SIBO diagnosed with glucose breath testing to be 19% (95% CI 11-29%), with same level of heterogeneity between studies (Q-test X^2^ =287·1, P <0·0001; I^2^ =95·5%, Figure 31).

**Diagnosis made using small bowel aspirate**

Homogeneity was observed in subgroup analysis of studies which were conducted in Asia, whilst all other subgroup analyses resulted in a similar level of heterogeneity (Figure 33).

Two studies [24, 30] was deemed to be at high risk of bias, and a repeat analysis was performed after excluding both studies and estimated the prevalence to be 16% (95% CI 6-25%), with the same level of heterogeneity between studies (Q-test X^2^ =63·9, P <0·0001; I^2^ =93·7%, Figure 34).

**Supplementary figure 1. Flow diagram showing results of literature search for BAD.**


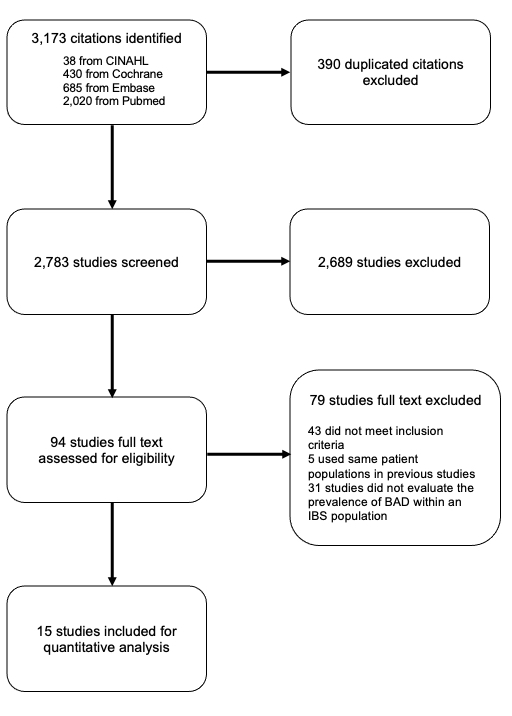


**Supplementary figure 2. Forest plots showing the estimated pooled prevalence of BAD in patients diagnosed with IBS, if the Rome III criteria were used by Shiha et al.**

**
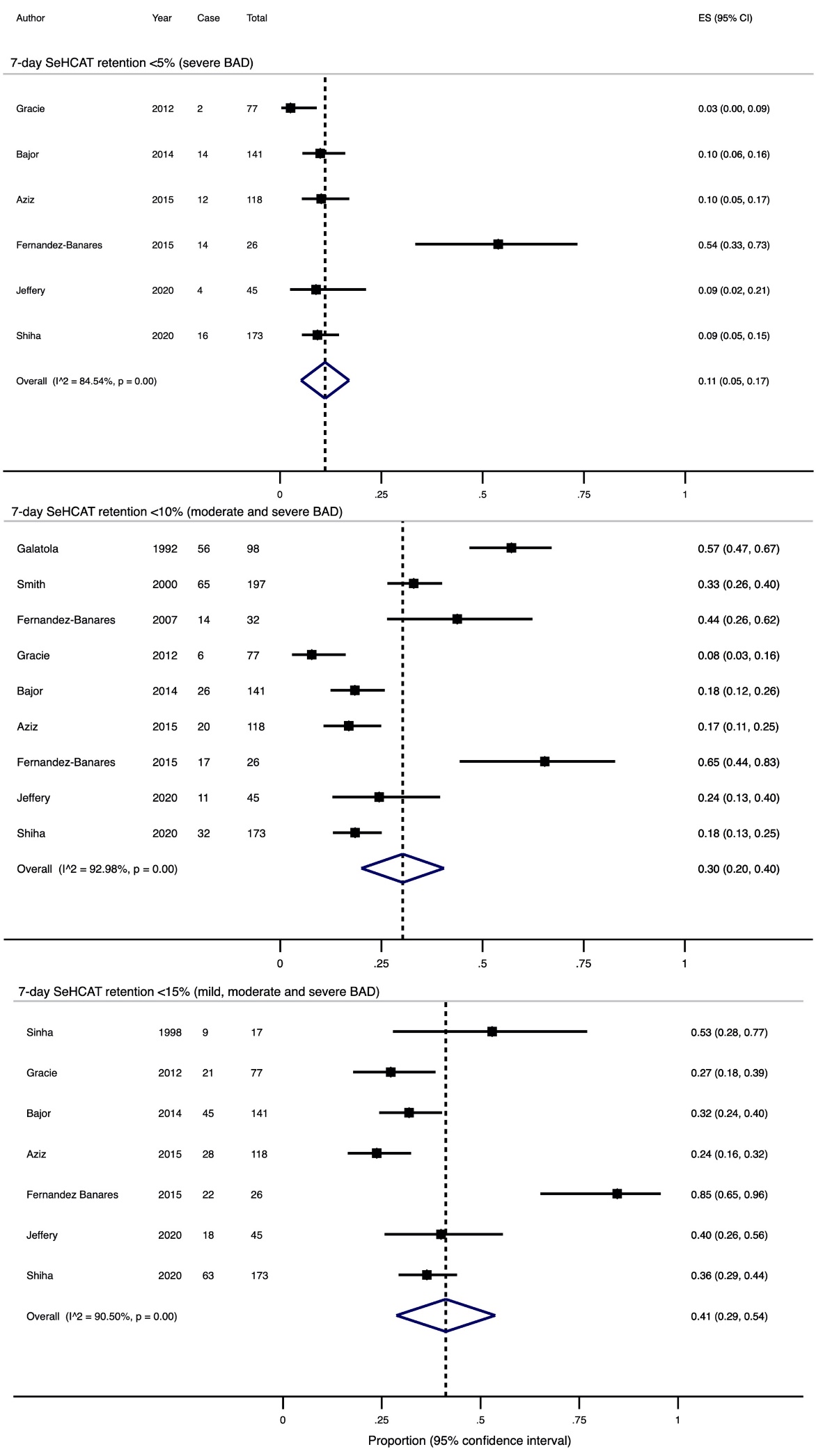
**

**Supplementary figure 3. Subgroup analyses of prevalence of BAD diagnosed with SeHCAT, if the Rome III criteria were used by Shiha et al.**

1. **7-day retention <5% (severe BAD)**

**
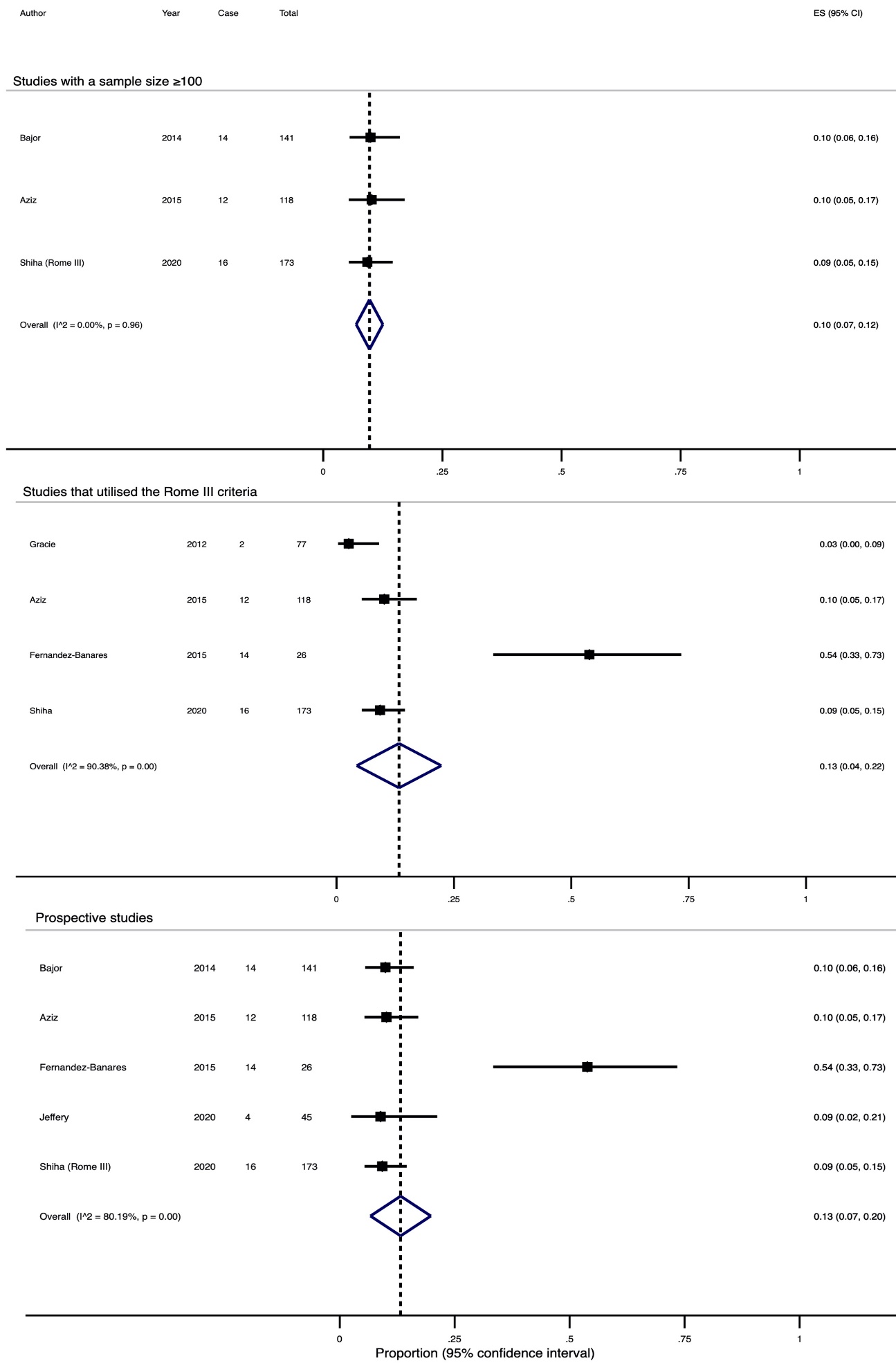
**

**Supplementary figure 3. Subgroup analyses of prevalence of BAD diagnosed with SeHCAT, if the Rome III criteria were used by Shiha et al.**

1. **7-day retention <10% (moderate and severe BAD)**

**
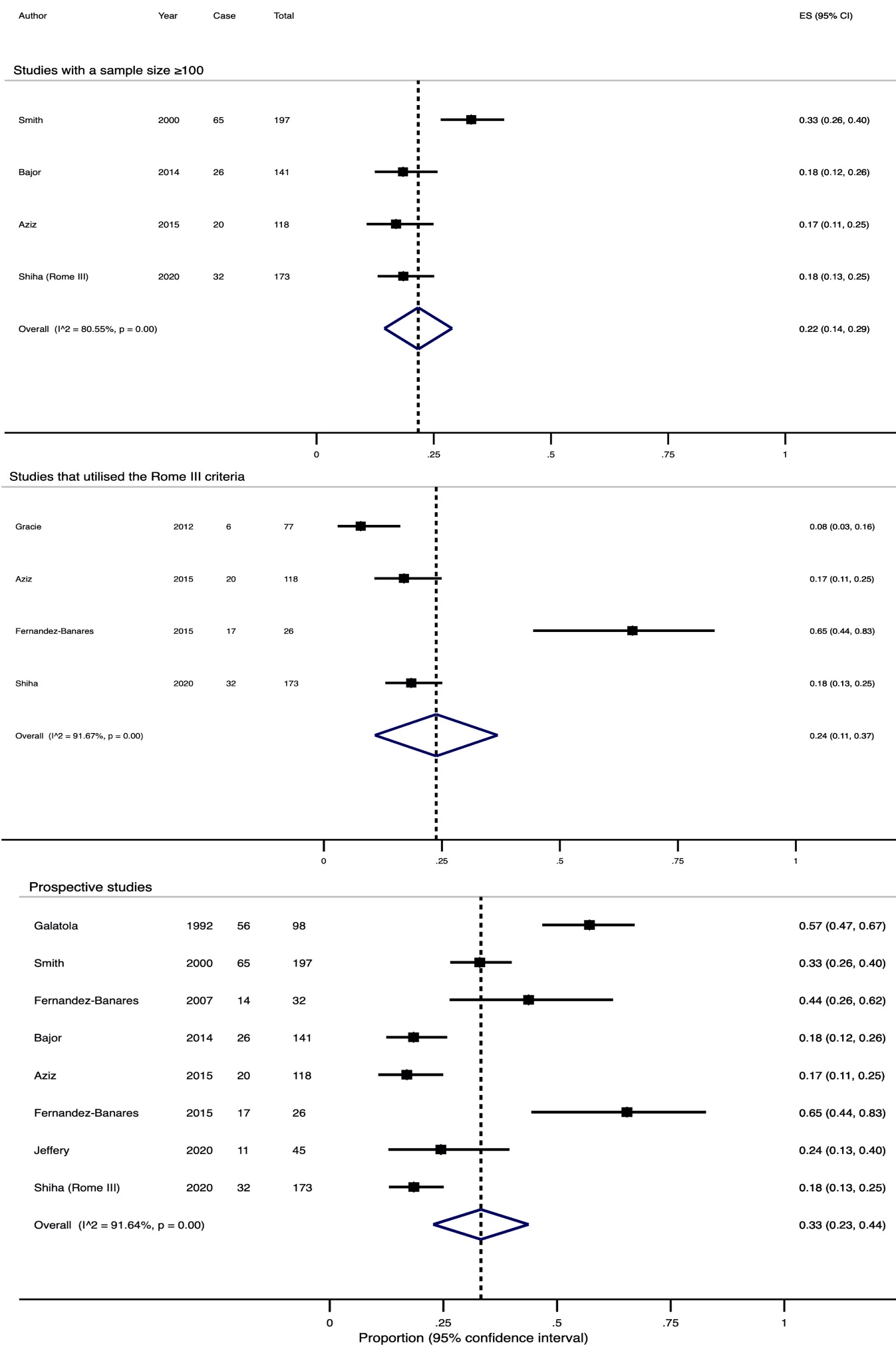
**

**Supplementary figure 3. Subgroup analyses of prevalence of BAD diagnosed with SeHCAT, if the Rome III criteria were used by Shiha et al.**

1. **7-day retention <15% (mild, moderate and severe BAD)**

**
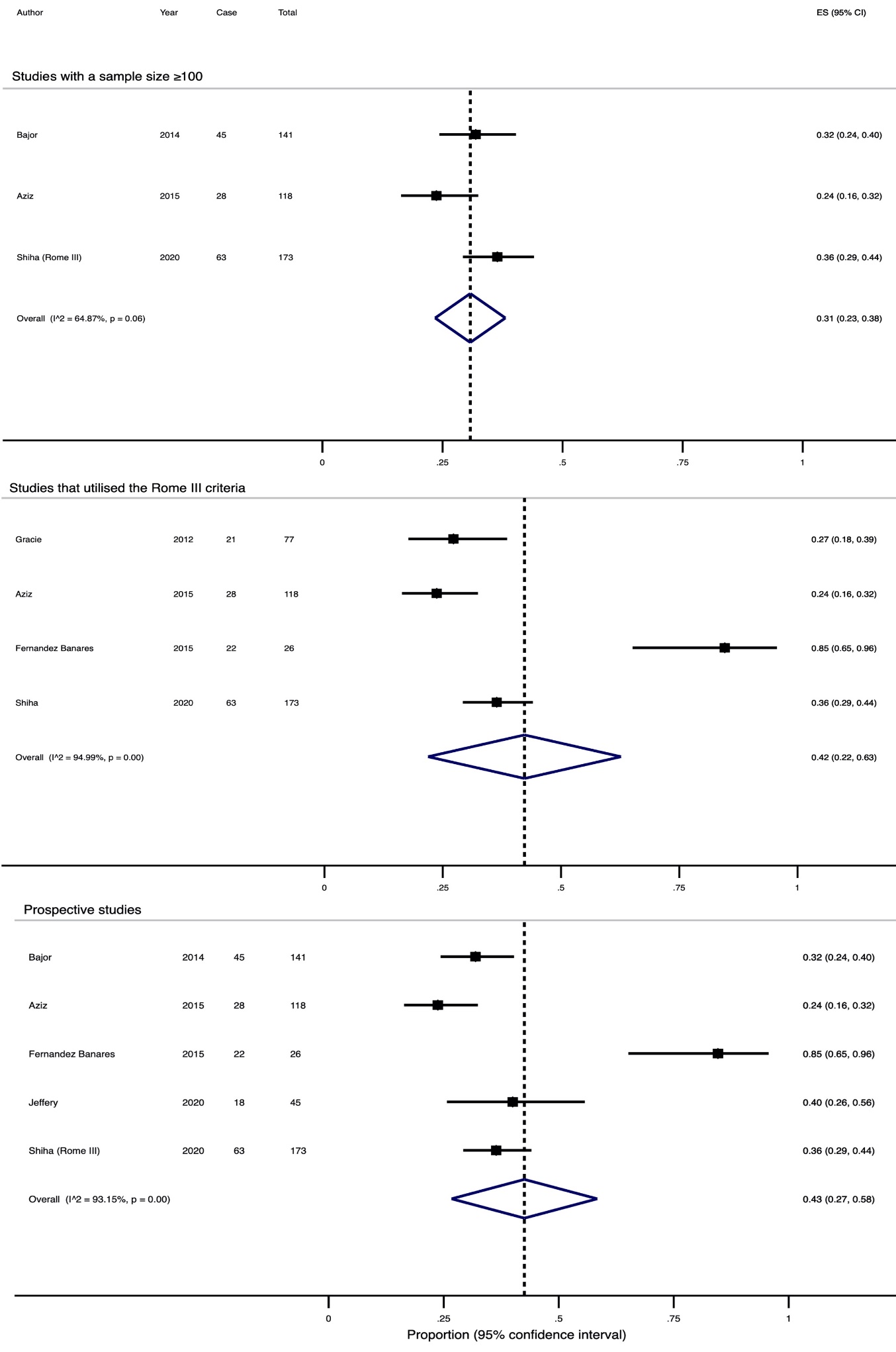
**

**Supplementary figure 4. Forest plots, after excluding one study with high risk of bias, showing the estimated pooled prevalence of BAD, if the Rome III criteria were used by Shiha et al.**

**
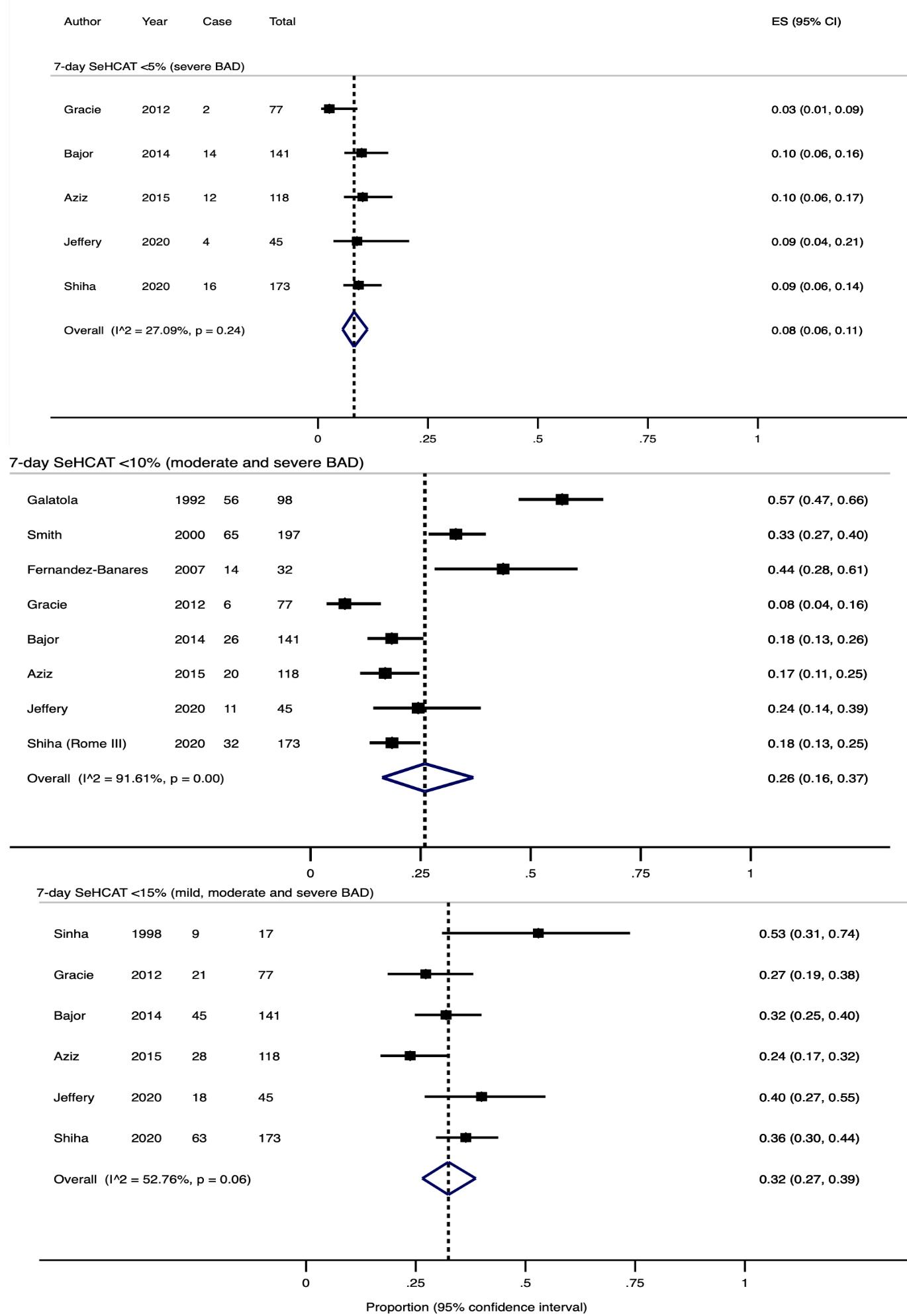
**

**Supplementary figure 5. A Forest plot showing the estimated pooled prevalence of BAD based upon an elevated level of 48-hour faecal bile acids.**

**
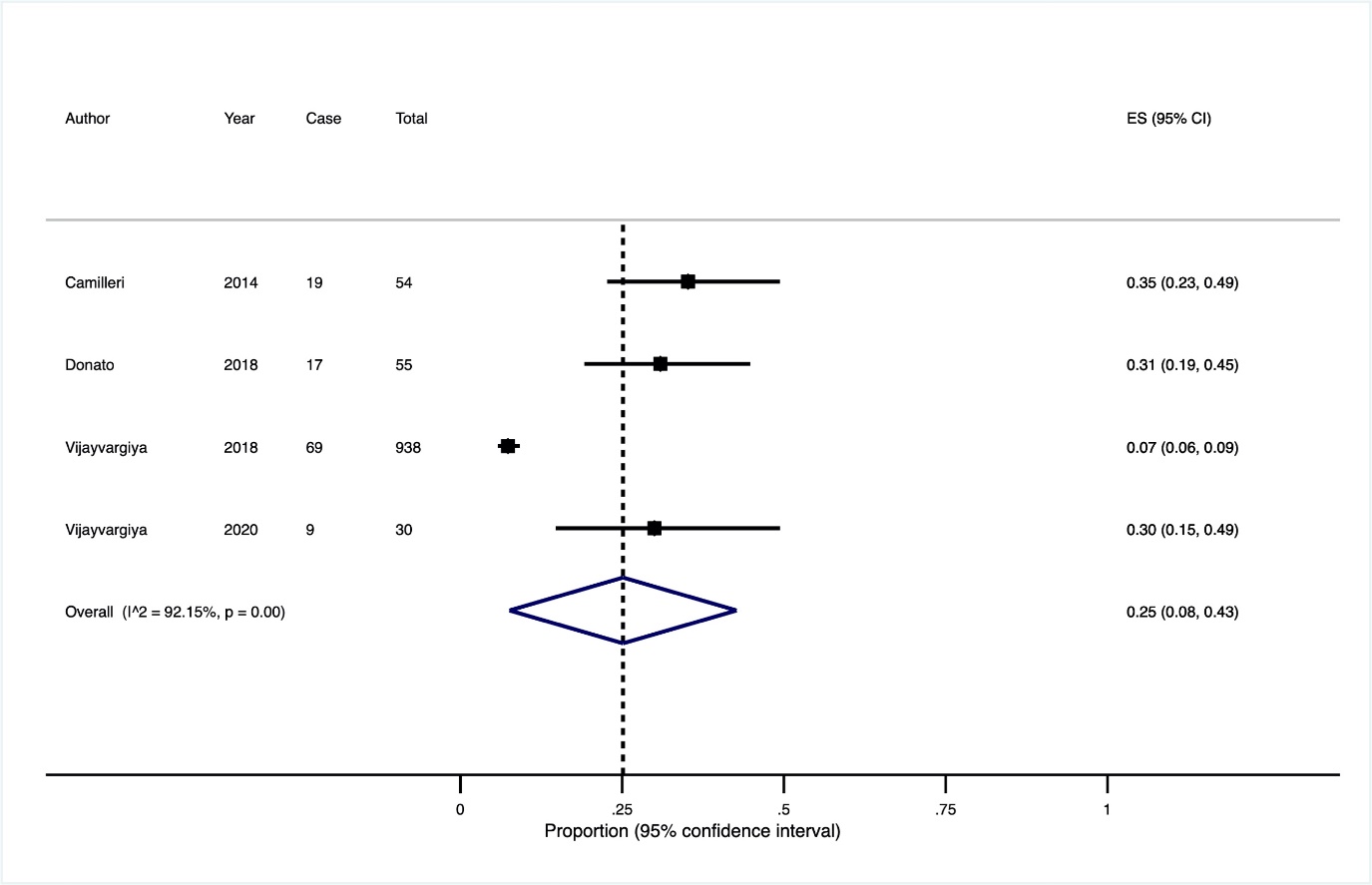
**

**Supplementary figure 6. A Forest plot showing the estimated pooled prevalence of BAD based upon an elevated level of 7α-C4.**

**
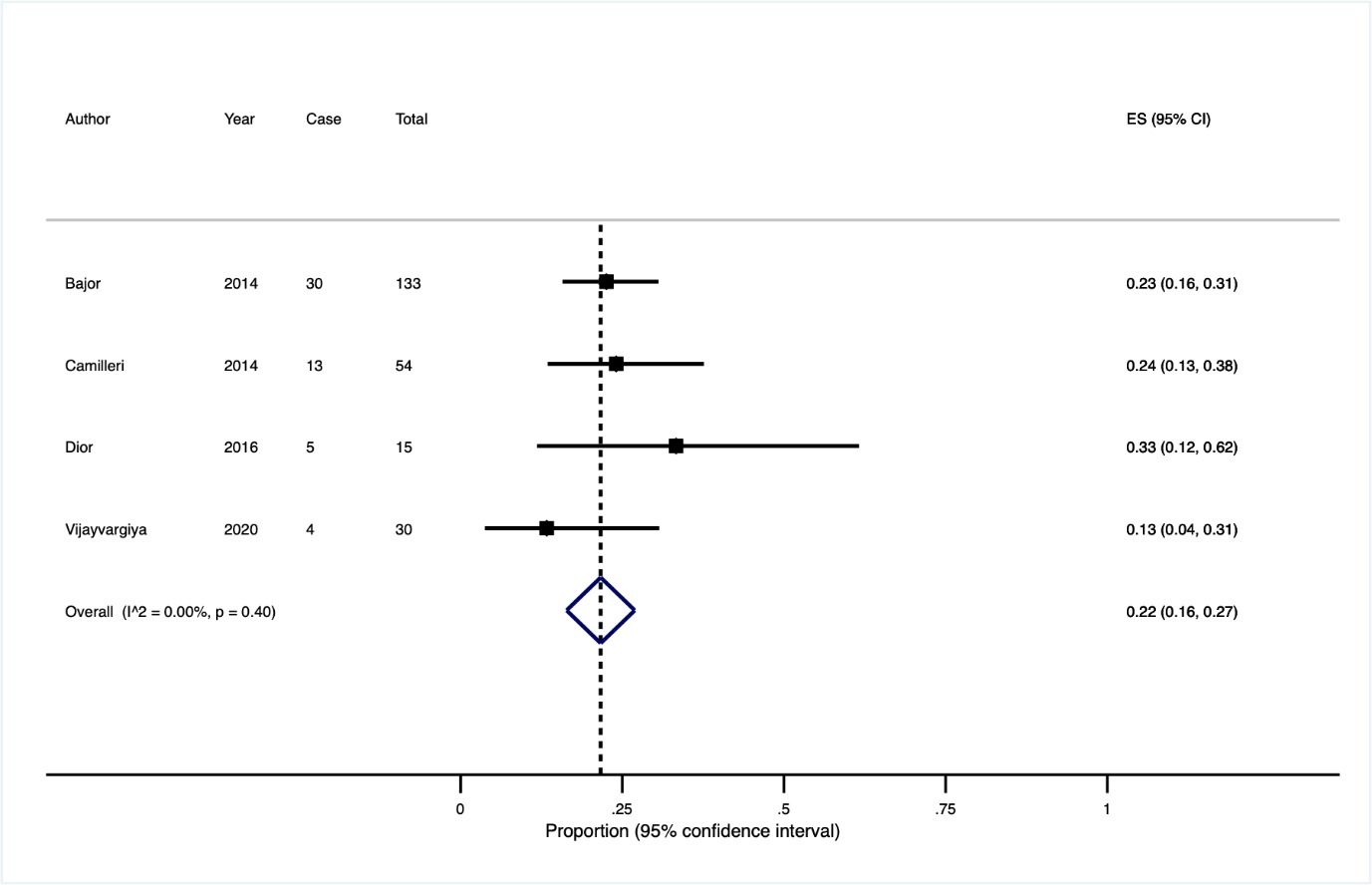
**

**Supplementary figure 7. Flow diagram showing results of literature search for CM.**


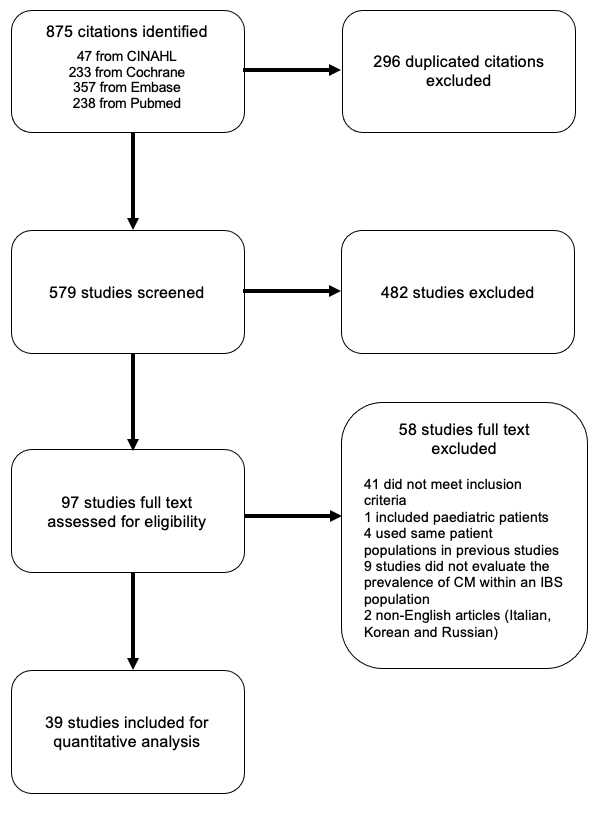


**Supplementary figure 8. Subgroup analyses of prevalence of lactose malabsorption diagnosed with breath testing using**

1. **studies with a sample size ≥100 and prospective studies**


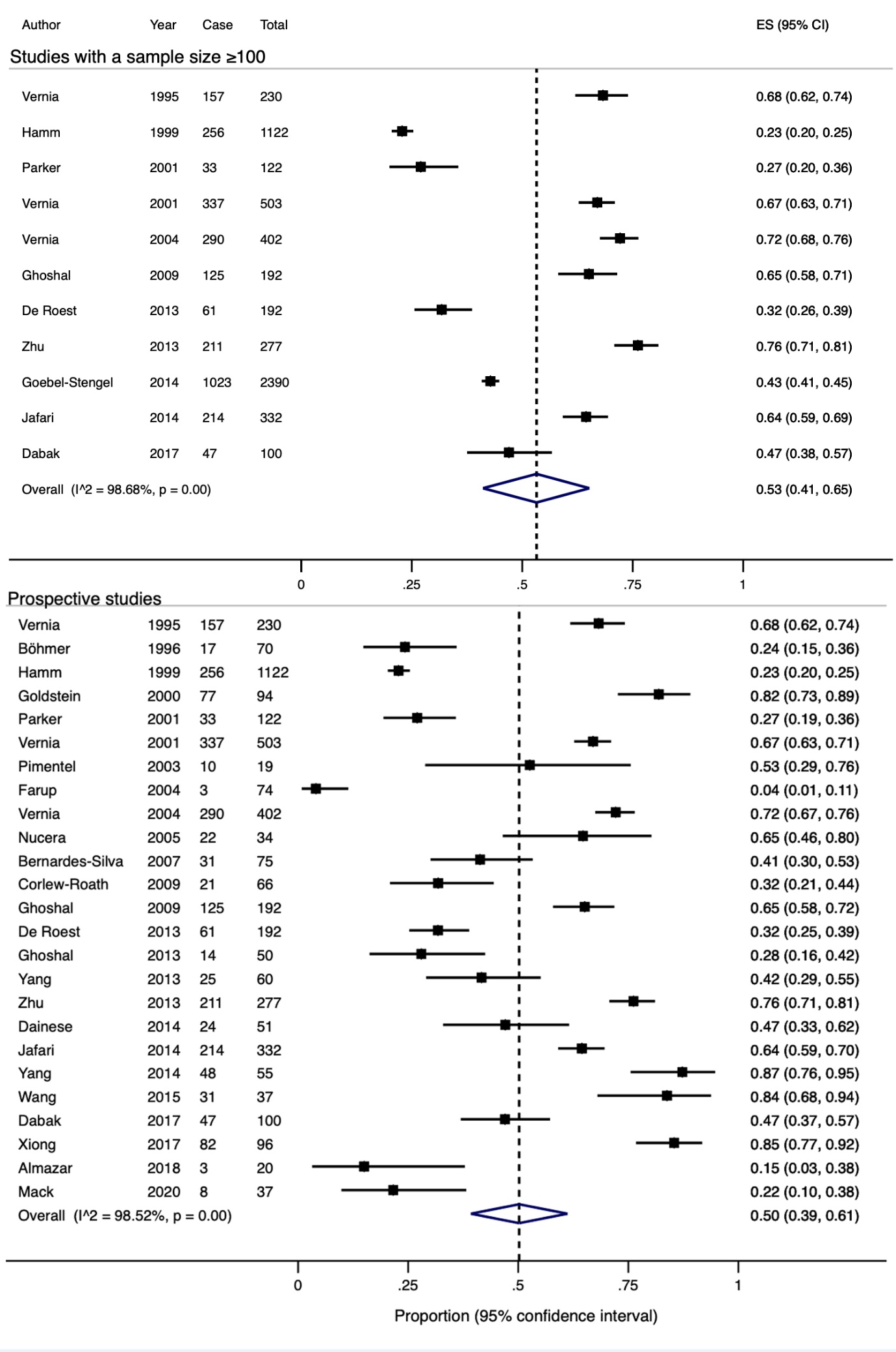


**Supplementary figure 8. Subgroup analyses of prevalence of lactose malabsorption diagnosed with breath testing using**

1. **the Rome criteria**


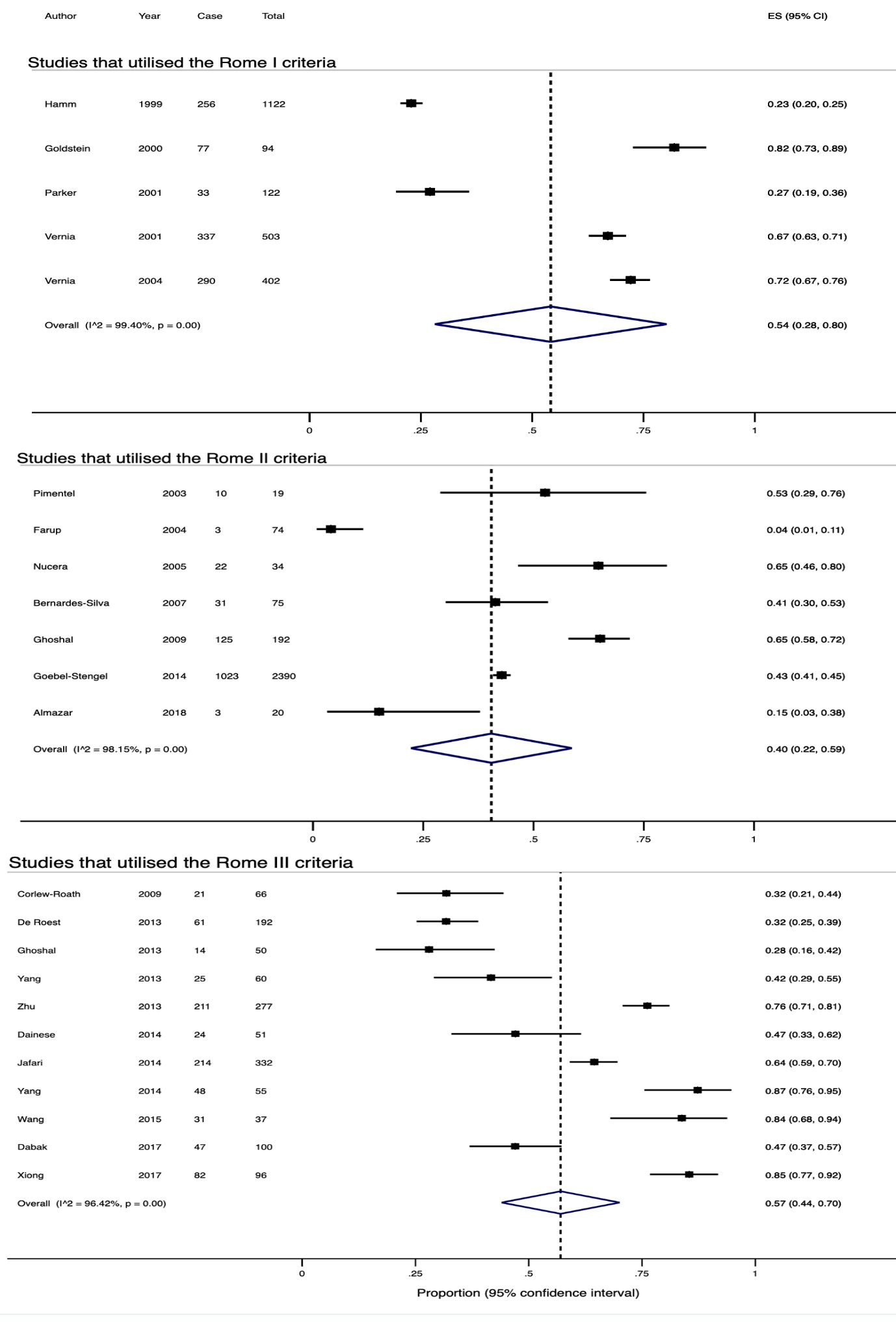


**Supplementary figure 8. Subgroup analyses of prevalence of lactose malabsorption diagnosed with breath testing using**

1.
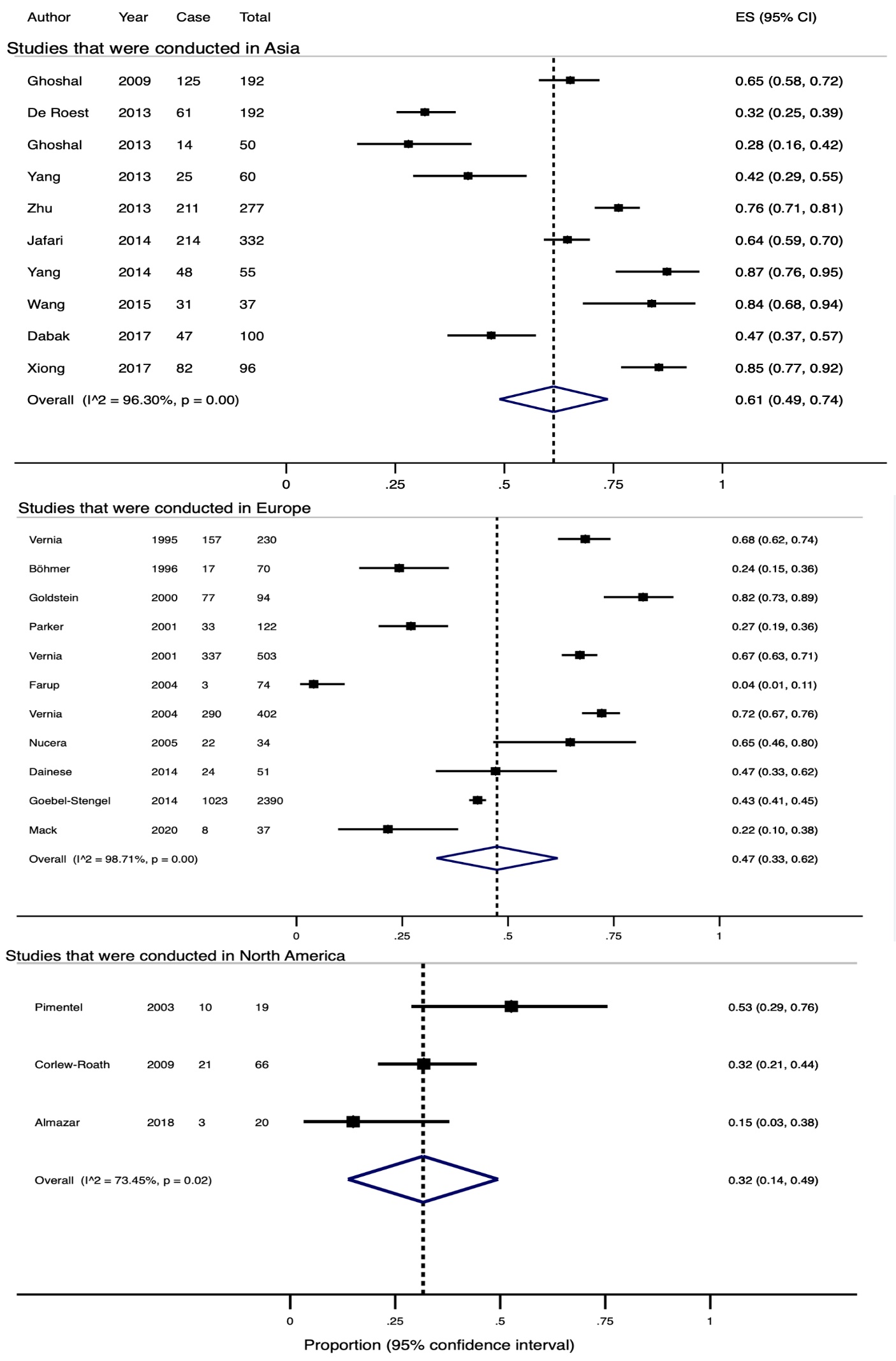
**study location**

**Supplementary figure 8. Subgroup analyses of prevalence of lactose malabsorption diagnosed with breath testing using**

1. **studies that used 25g and 50g lactose as the test substrate**

**
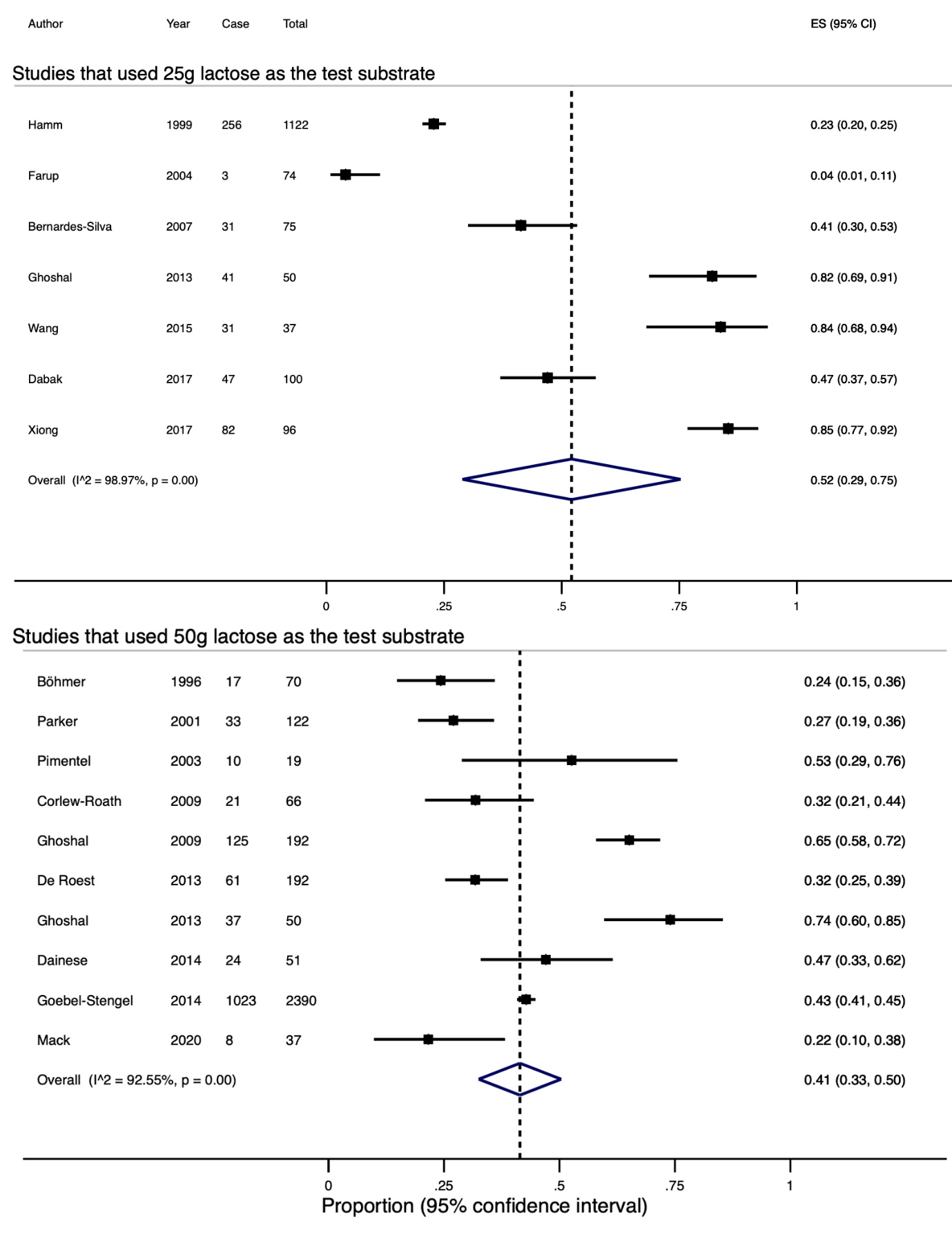
**

**Supplementary figure 9. A funnel plot indicated substantial small-study effects or publication bias among included studies of lactose malabsorption.**


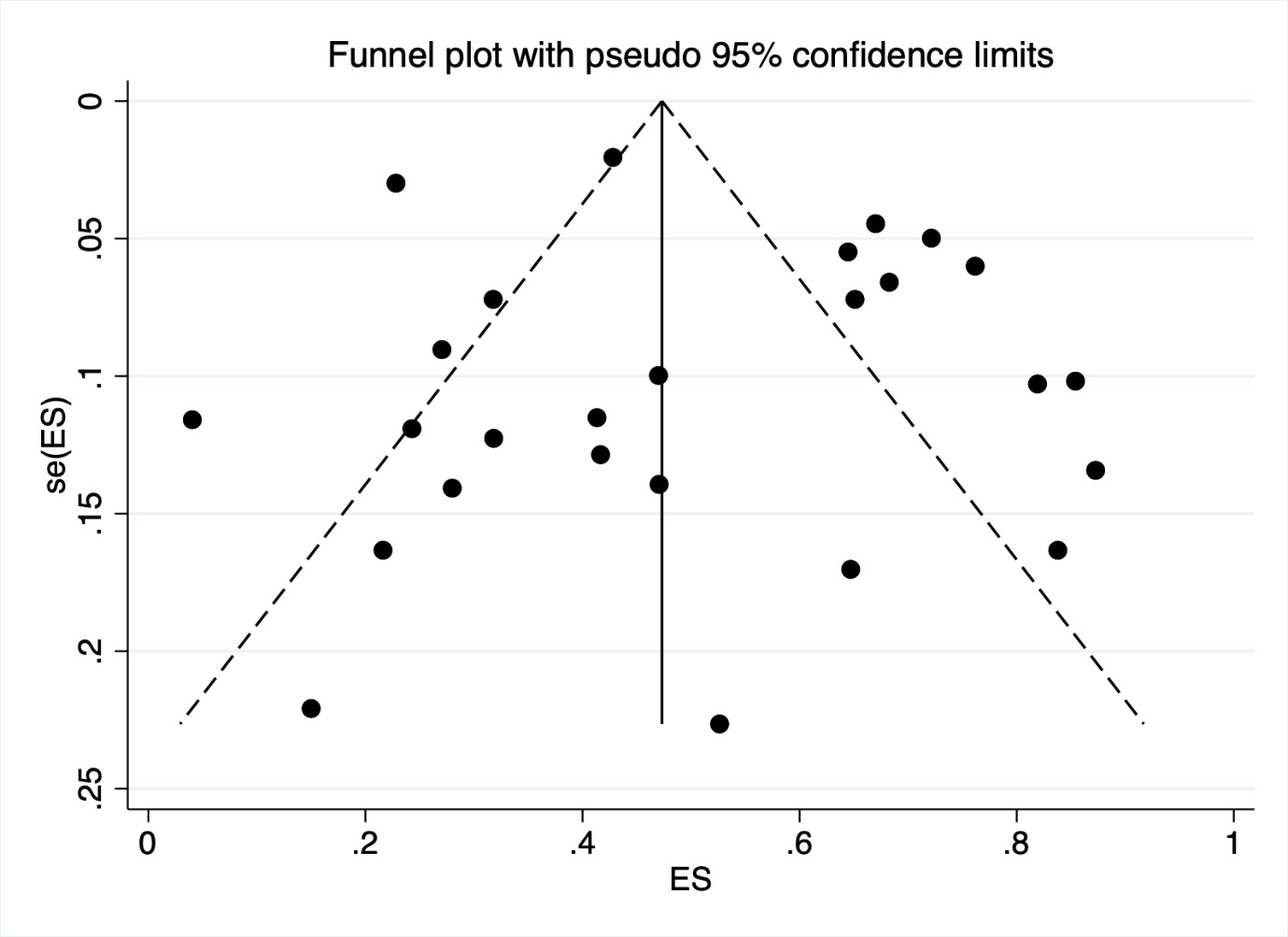


**Supplementary figure 10. A Forest plot of the 22 studies, after excluding four studies with high risks of bias, showing the estimated pooled prevalence of lactose malabsorption using breath testing.**

**
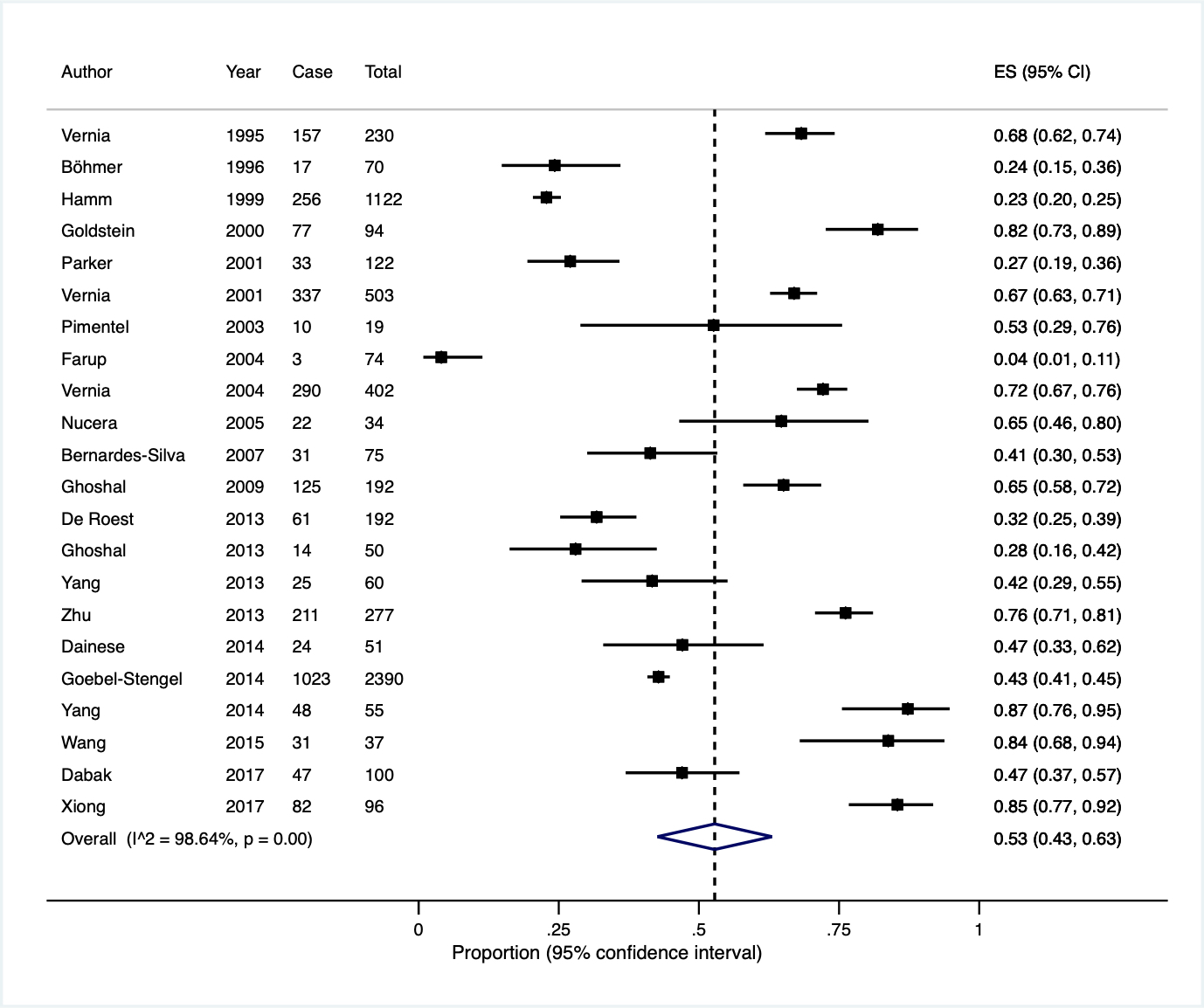
**

**Supplementary figure11. Forest plots showing the estimated pooled prevalence of lactose intolerance using hydrogen breath testing (HBT) and genotyping.**

**
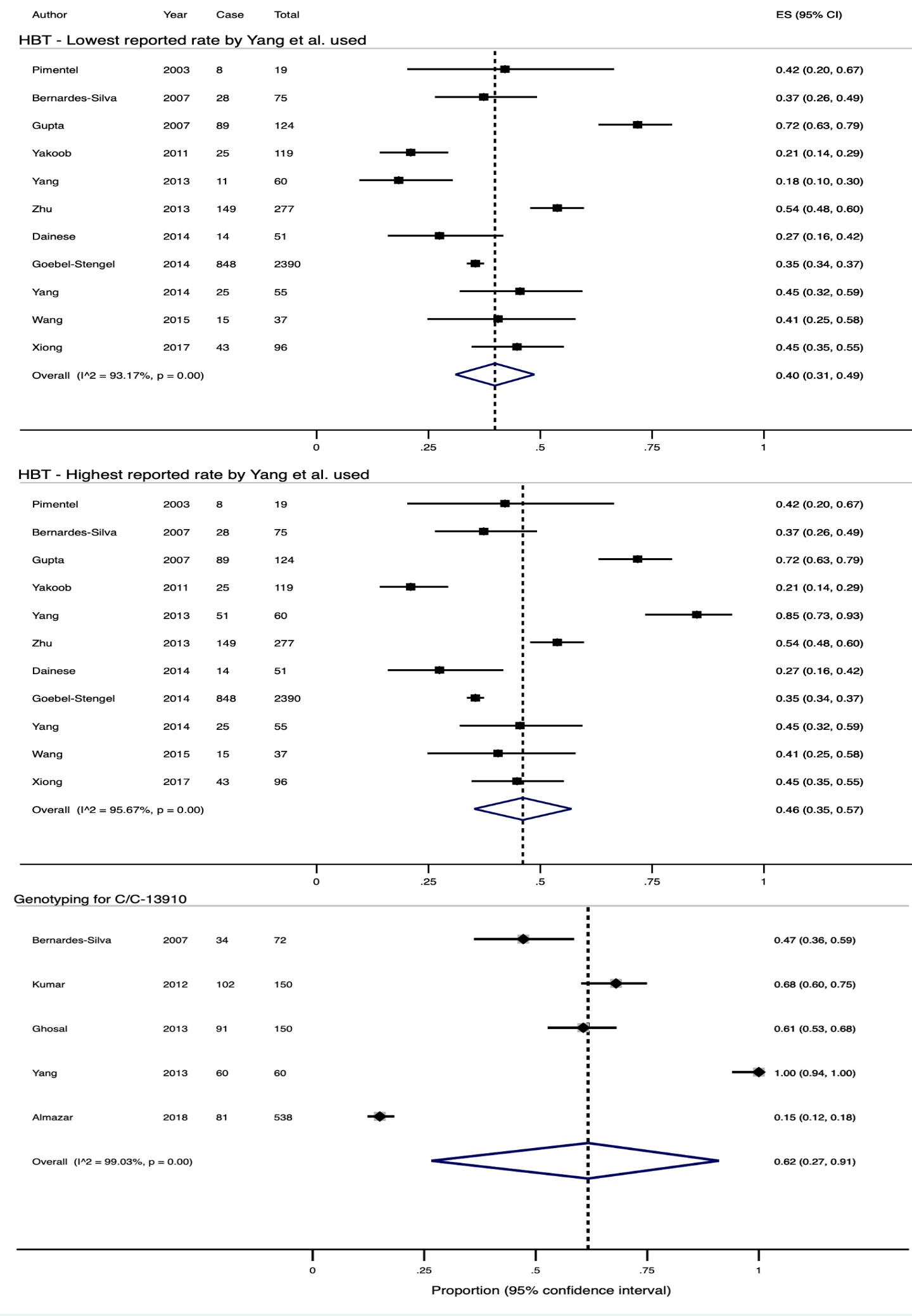
**

**Supplementary figure 12. A Forest plot of the four studies, after excluding one study with high risk of bias, showing the estimated pooled prevalence of lactose malabsorption using genotyping (C/C-13910 gene).**

**
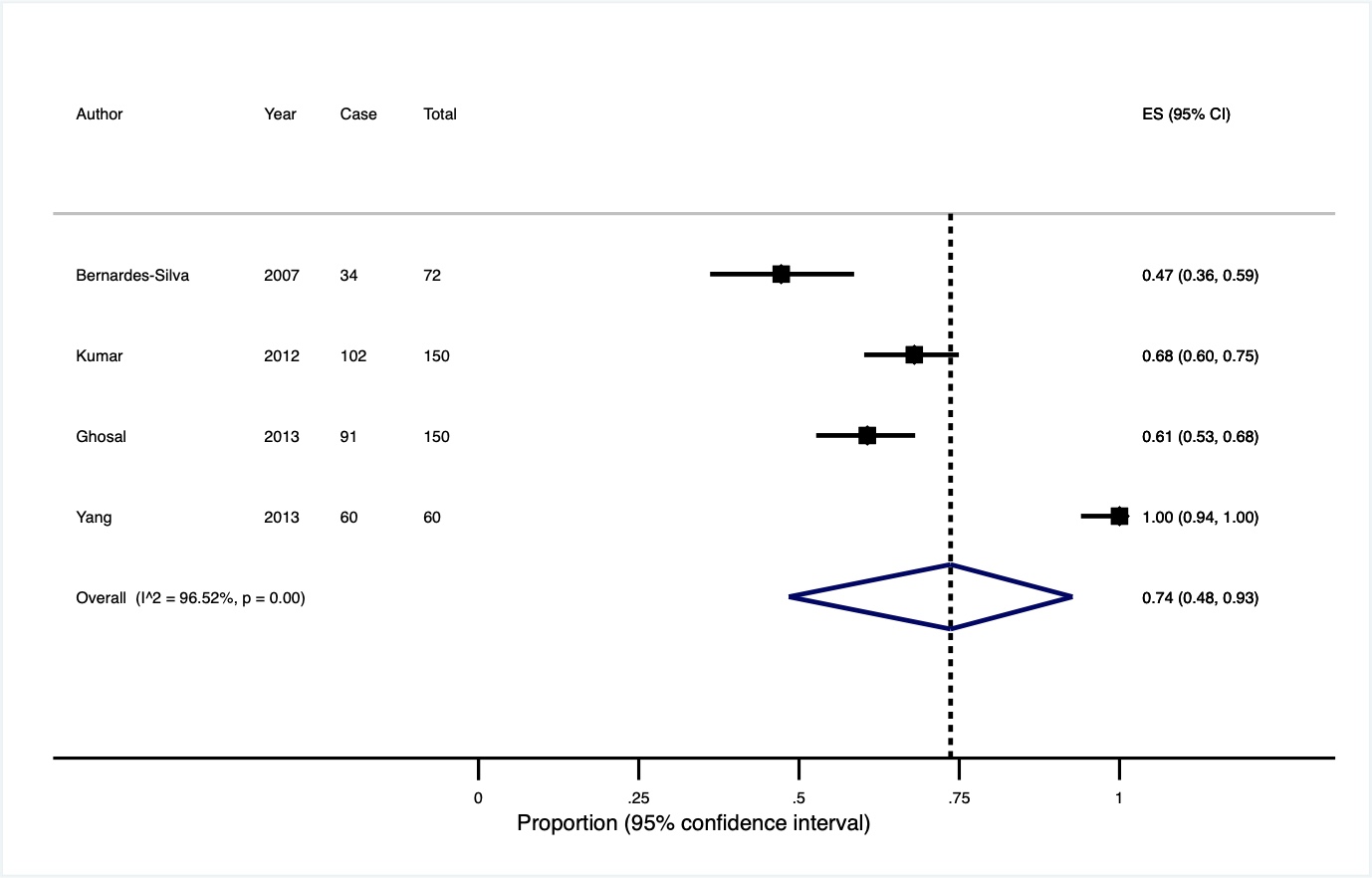
**

**Supplementary figure 13. Subgroup analyses of prevalence of fructose malabsorption diagnosed with breath testing using**

1. **studies with a sample size ≥100, prospective studies and studies that used 25g of fructose as the test substrate**

**
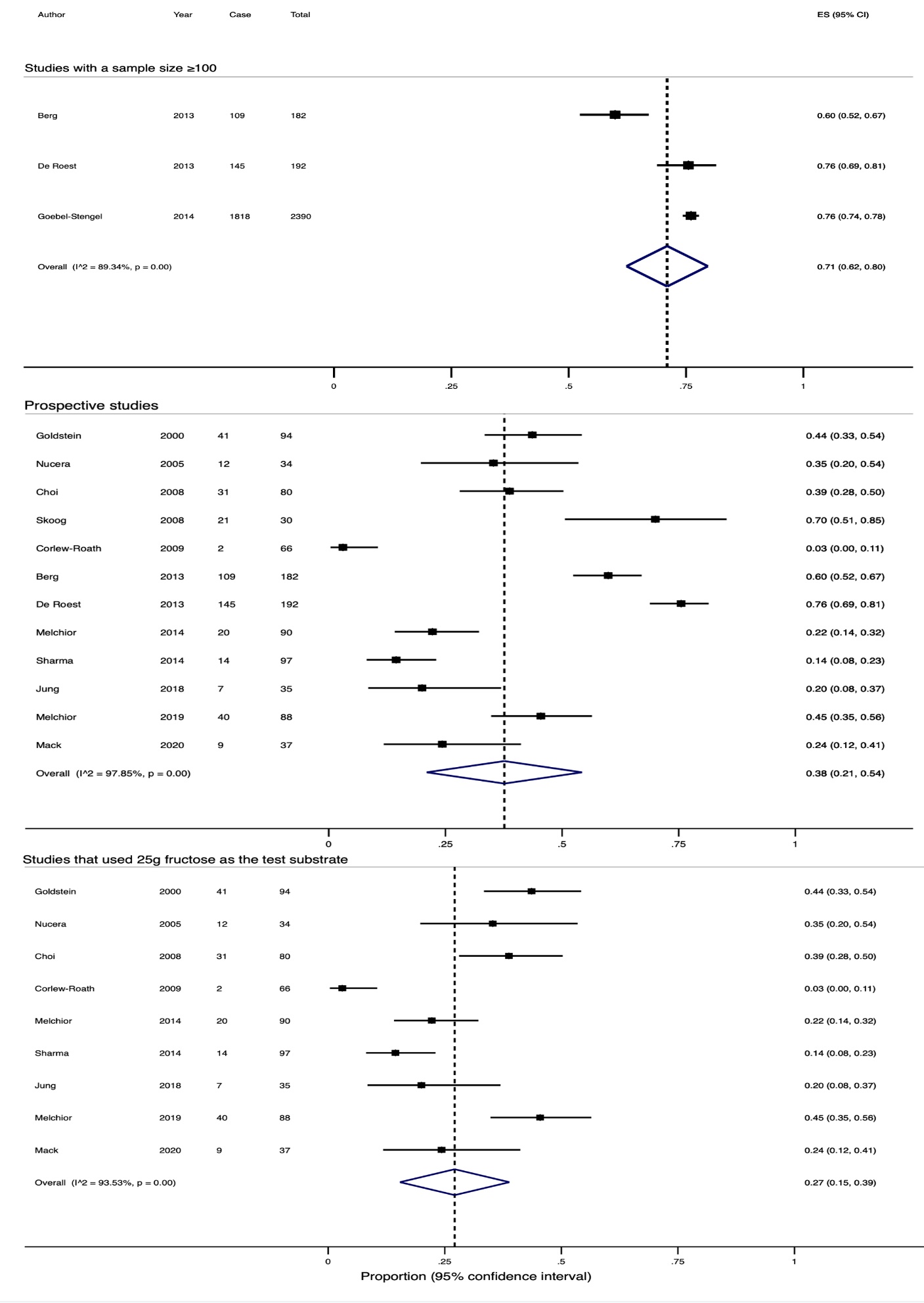
**

**Supplementary figure 13. Subgroup analyses of prevalence of fructose malabsorption diagnosed with breath testing using**

1. **the Rome criteria**

**
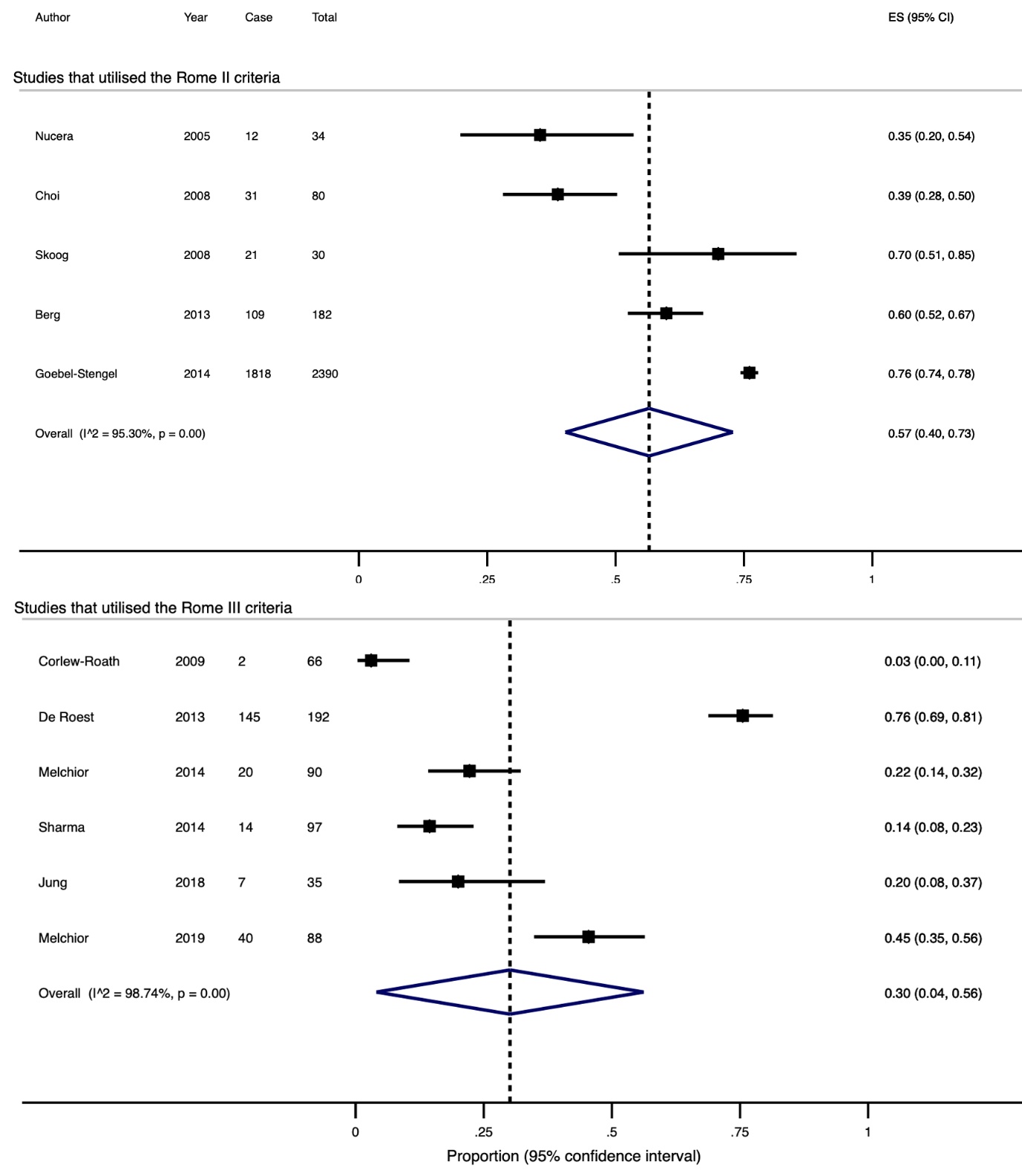
**

**Supplementary figure 13. Subgroup analyses of prevalence of fructose malabsorption diagnosed with breath testing using**

1. **study location**

**
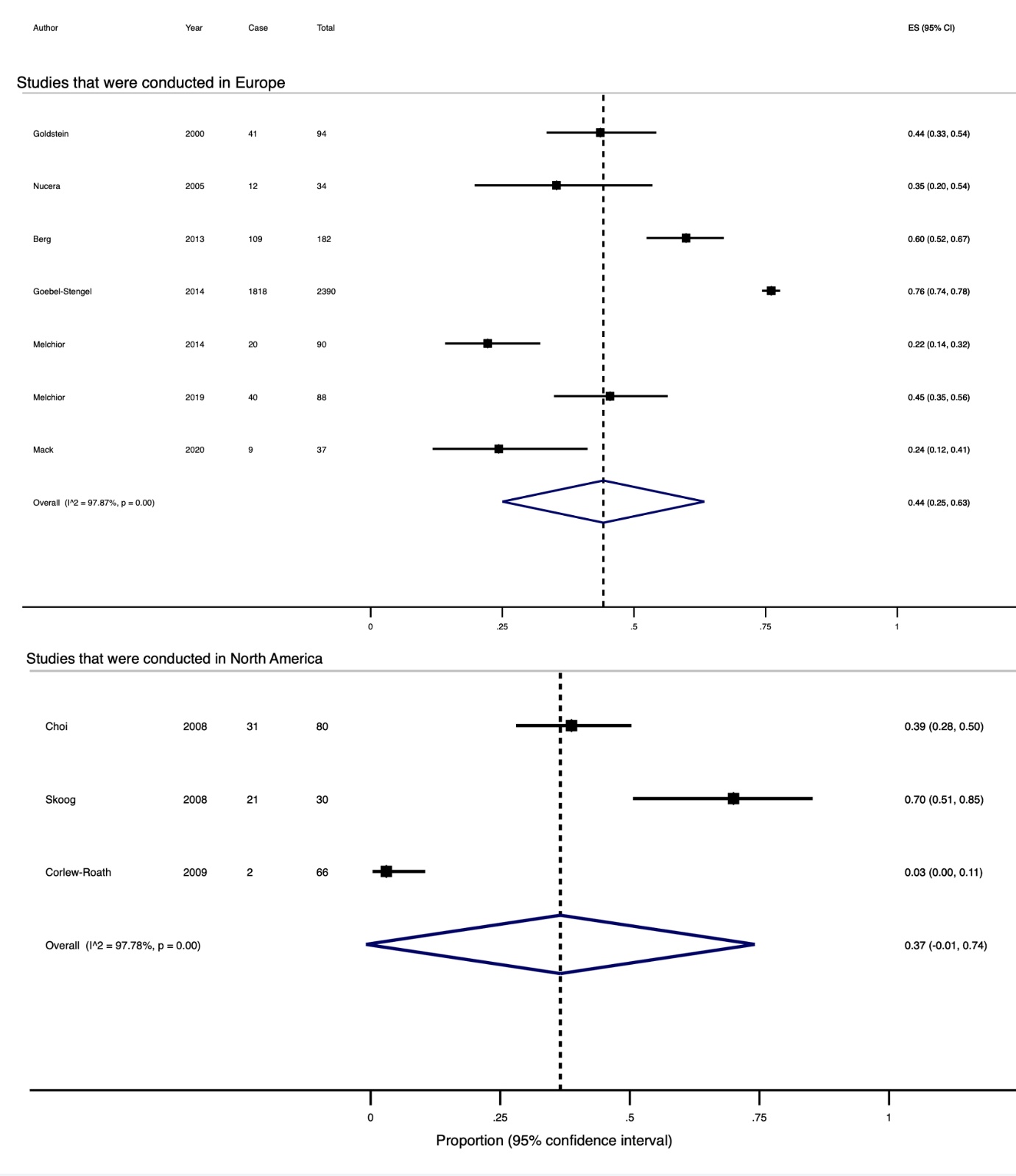
**

**Supplementary figure 14. A funnel plot indicated substantial small-study effects or publication bias among included studies of fructose malabsorption.**

**
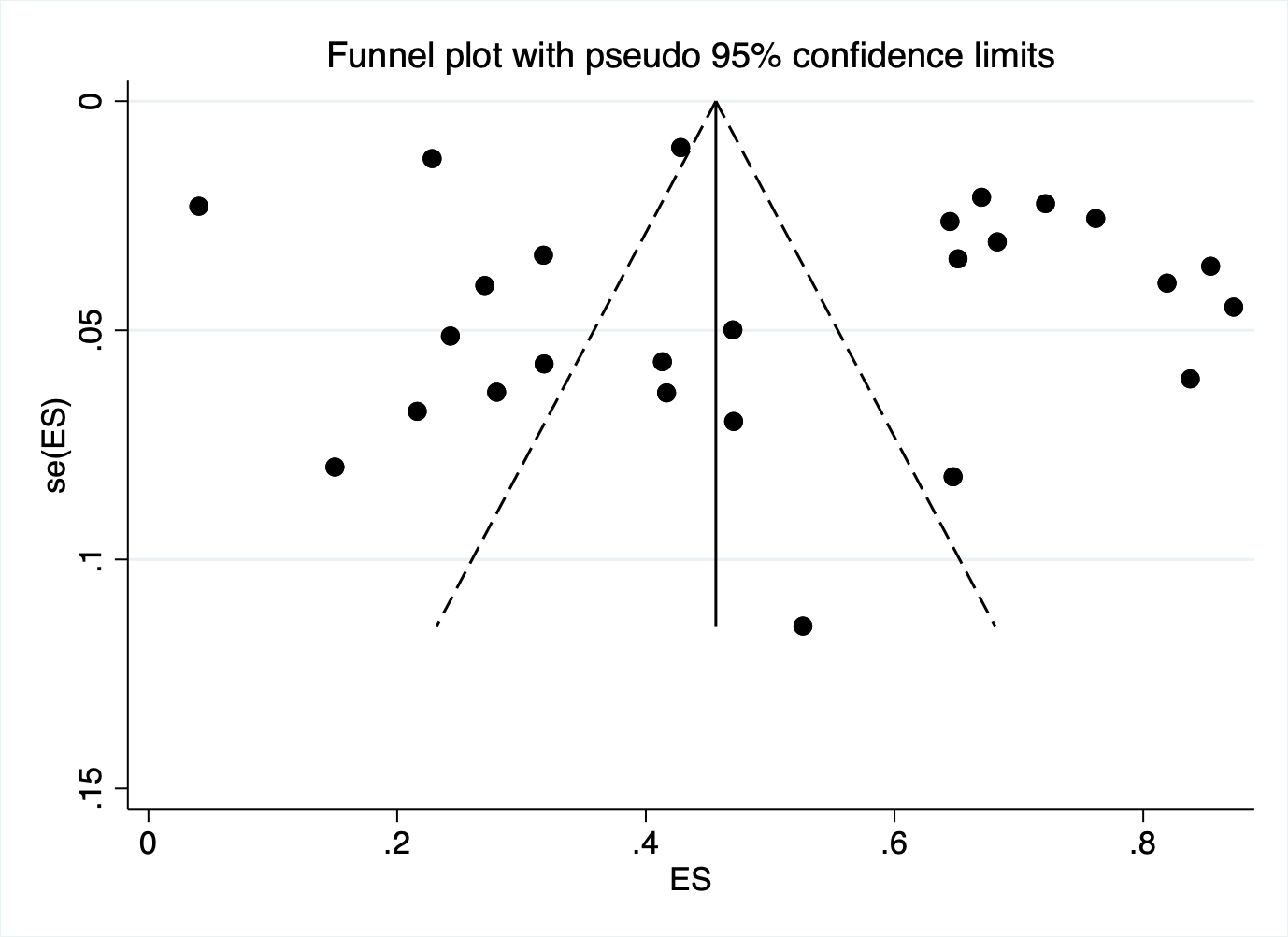
**

**Supplementary figure 15.** **A Forest plot of the nine studies, after excluding four studies with high risks of bias, showing the estimated pooled prevalence of fructose malabsorption using breath testing.**

**
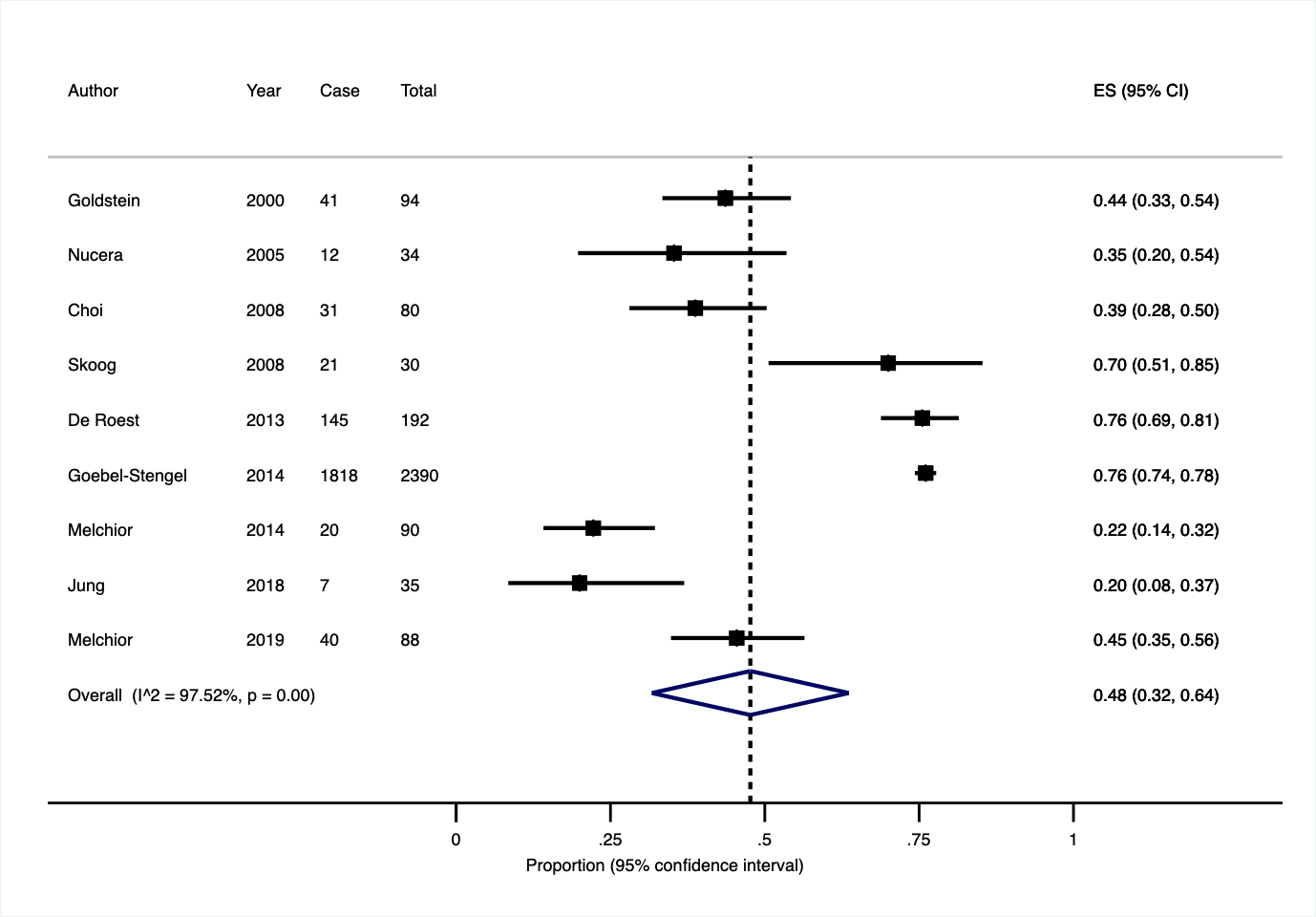
**

**Supplementary figure 16. A Forest plot showing the estimated pooled prevalence of fructose intolerance.**

**
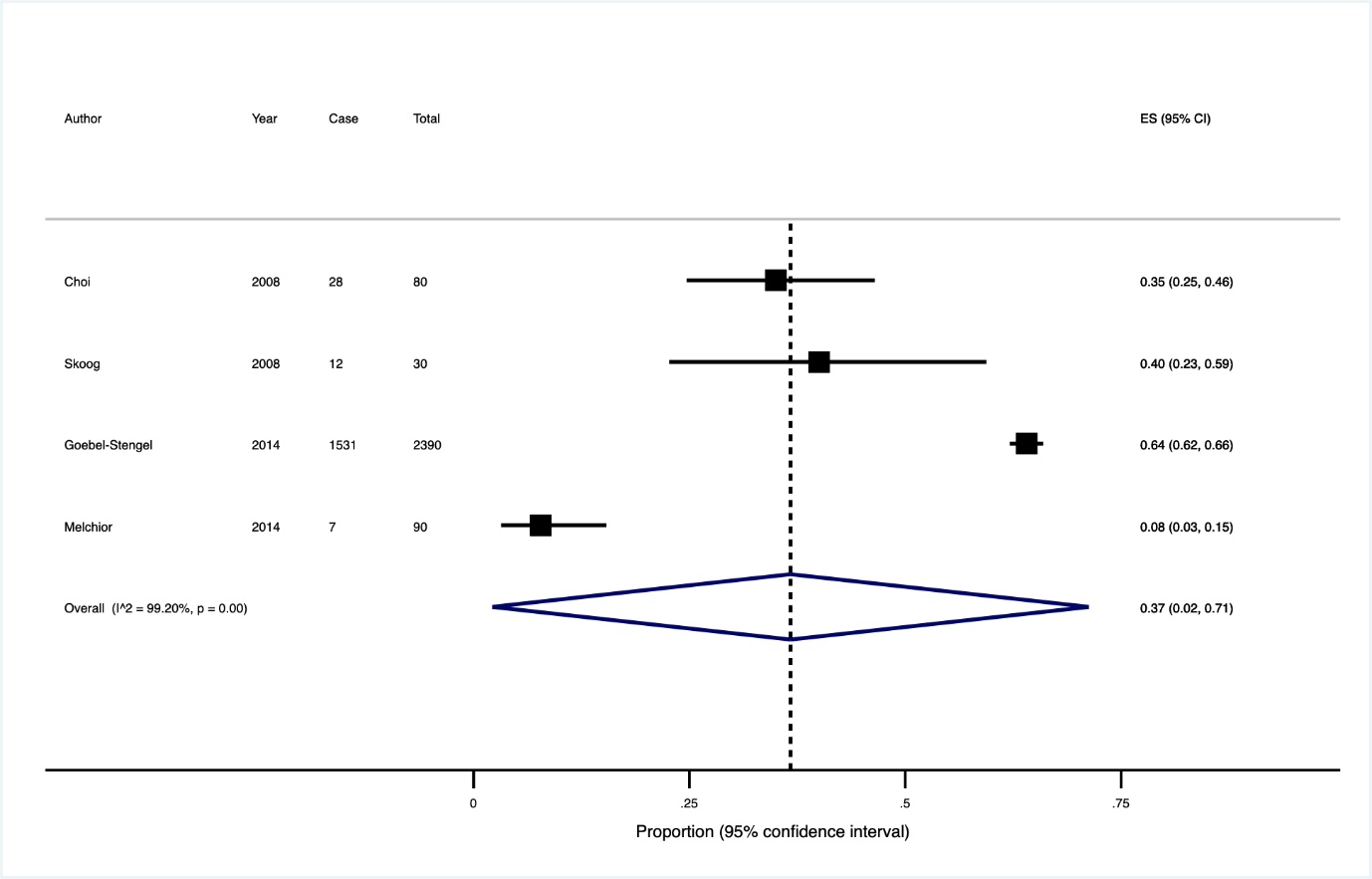
**

**Supplementary figure 17. A Forest plot showing the estimated pooled prevalence of sorbitol malabsorption using breath testing.**

**
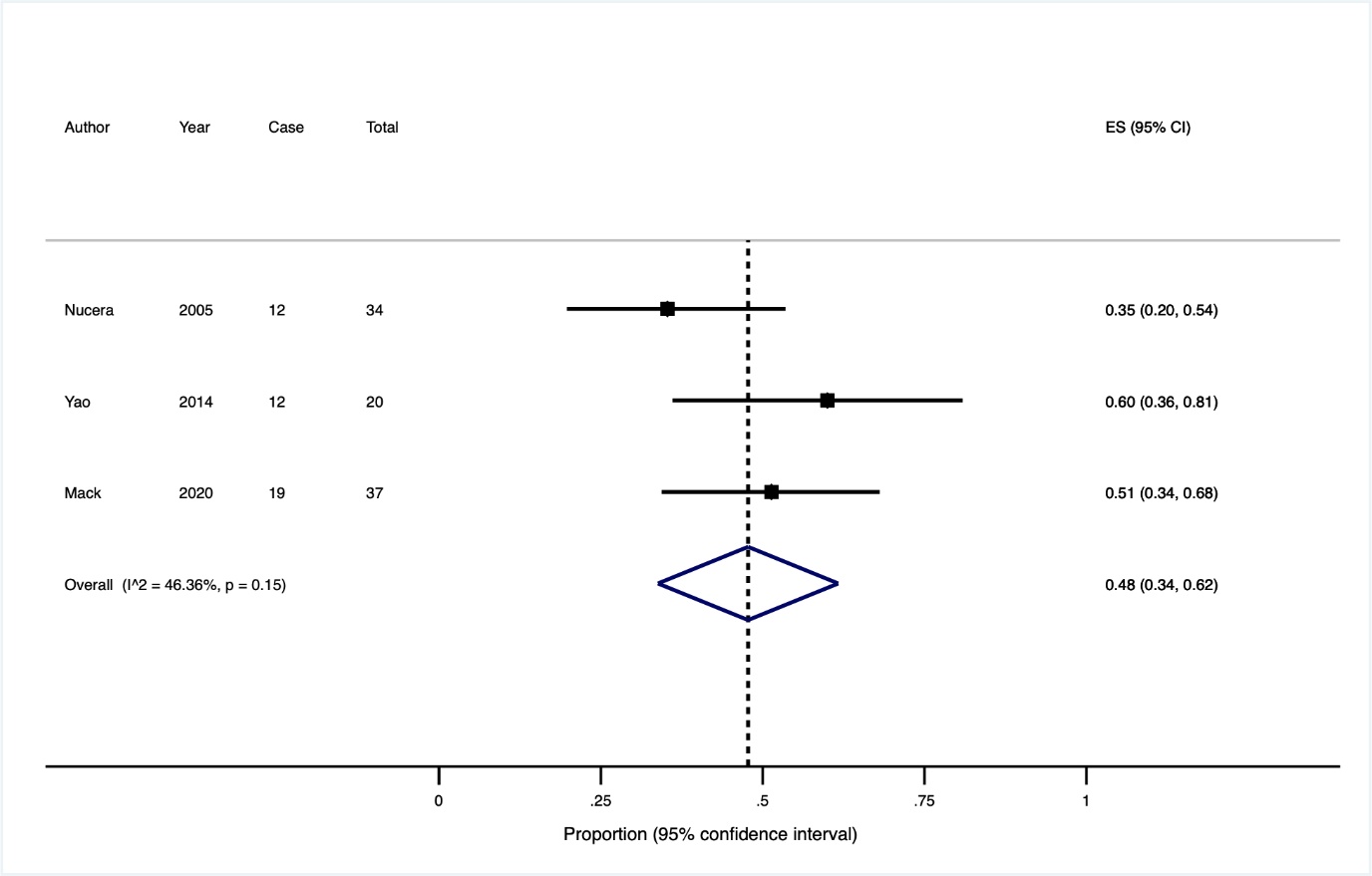
**

**Supplementary figure 18. Flow diagram showing results of literature search for MC.**


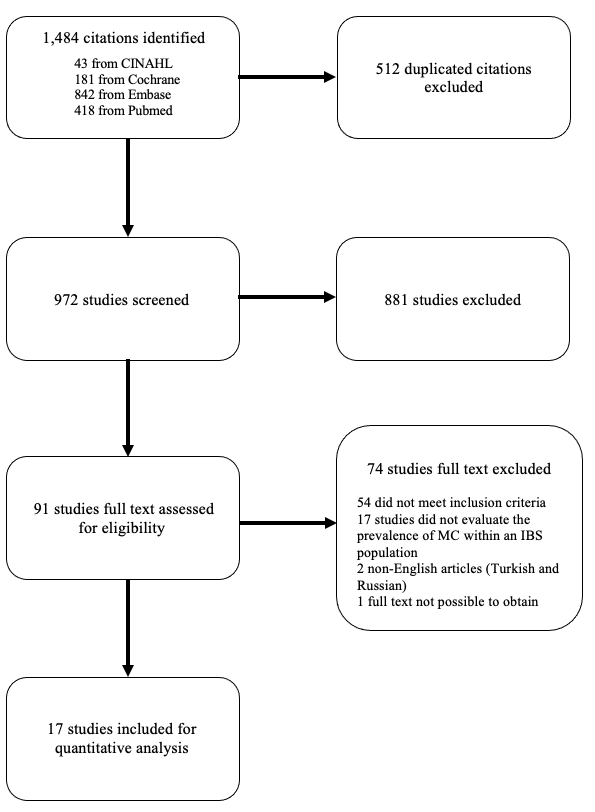


**Supplementary figure 19. A Forest plot of the 14 studies, after excluding three studies with high risks of bias, showing the estimated pooled prevalence of microscopic colitis.**

**
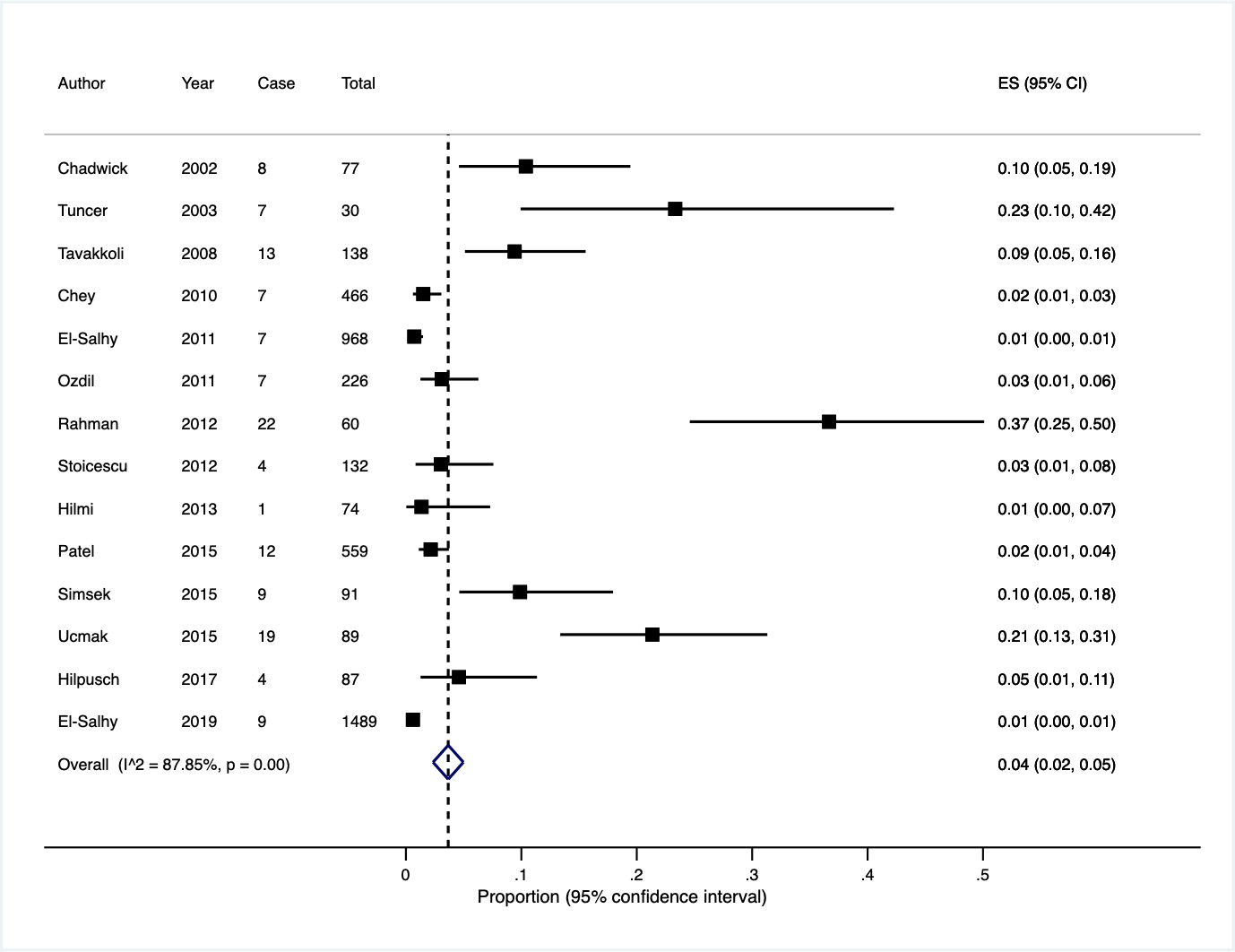
**

**Supplementary figure 20. Subgroup analyses of prevalence of lymphocytic colitis using**

1. **studies with a sample size ≥100 and prospective studies**

**
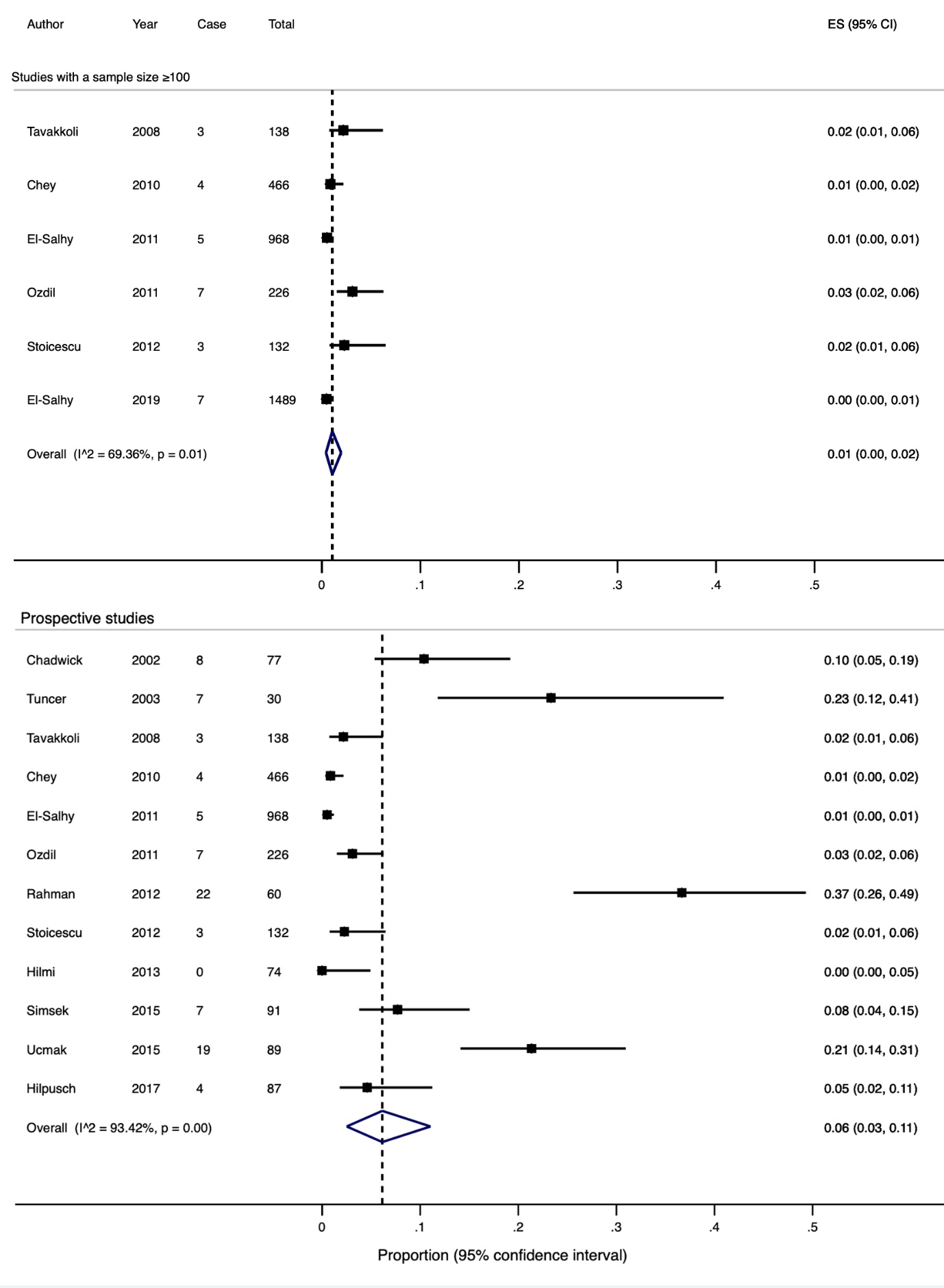
Supplementary figure 20. Subgroup analyses of prevalence of lymphocytic colitis using**

1. **the Rome criteria**

**
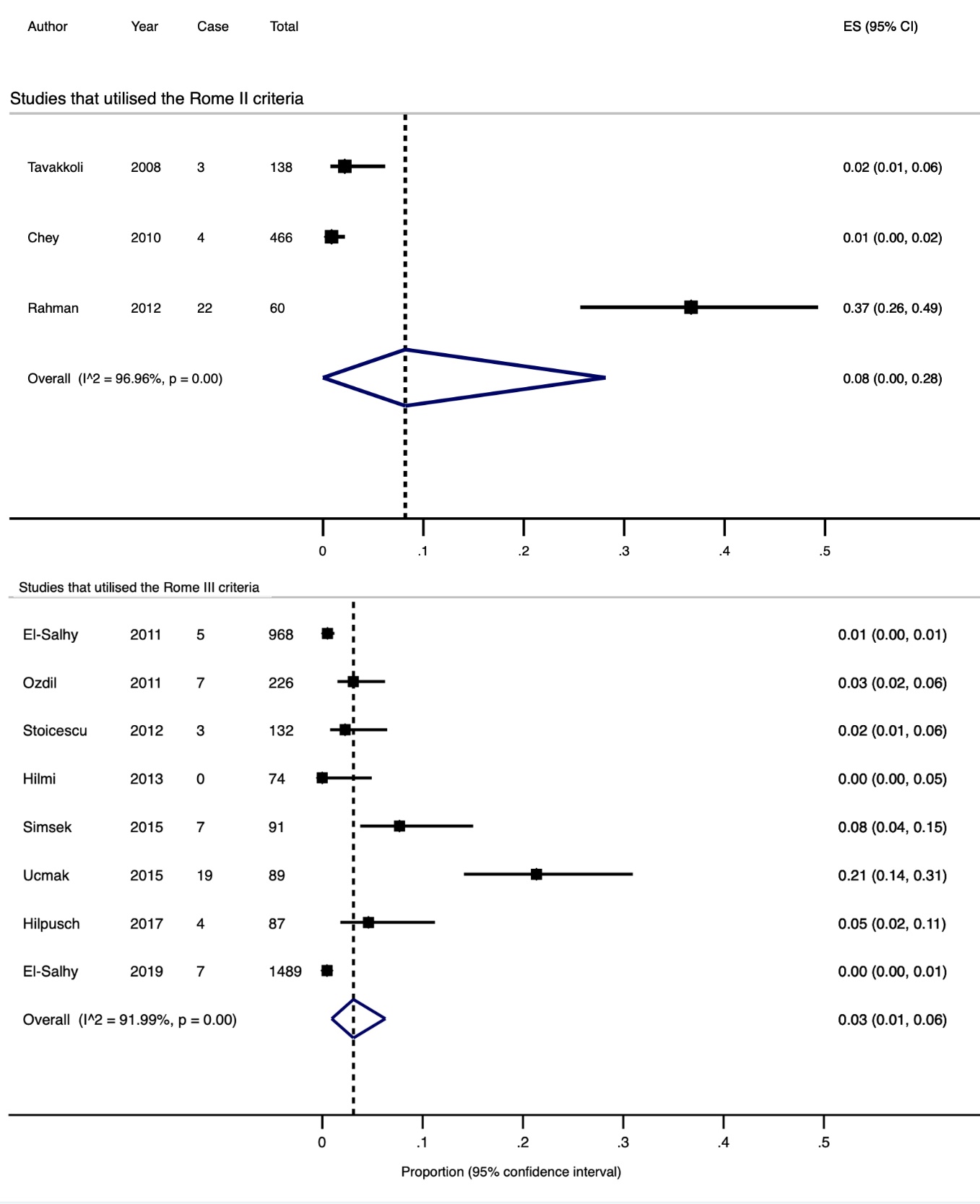
**

**Supplementary figure 20. Subgroup analyses of prevalence of lymphocytic colitis using**

1. **study location**

**
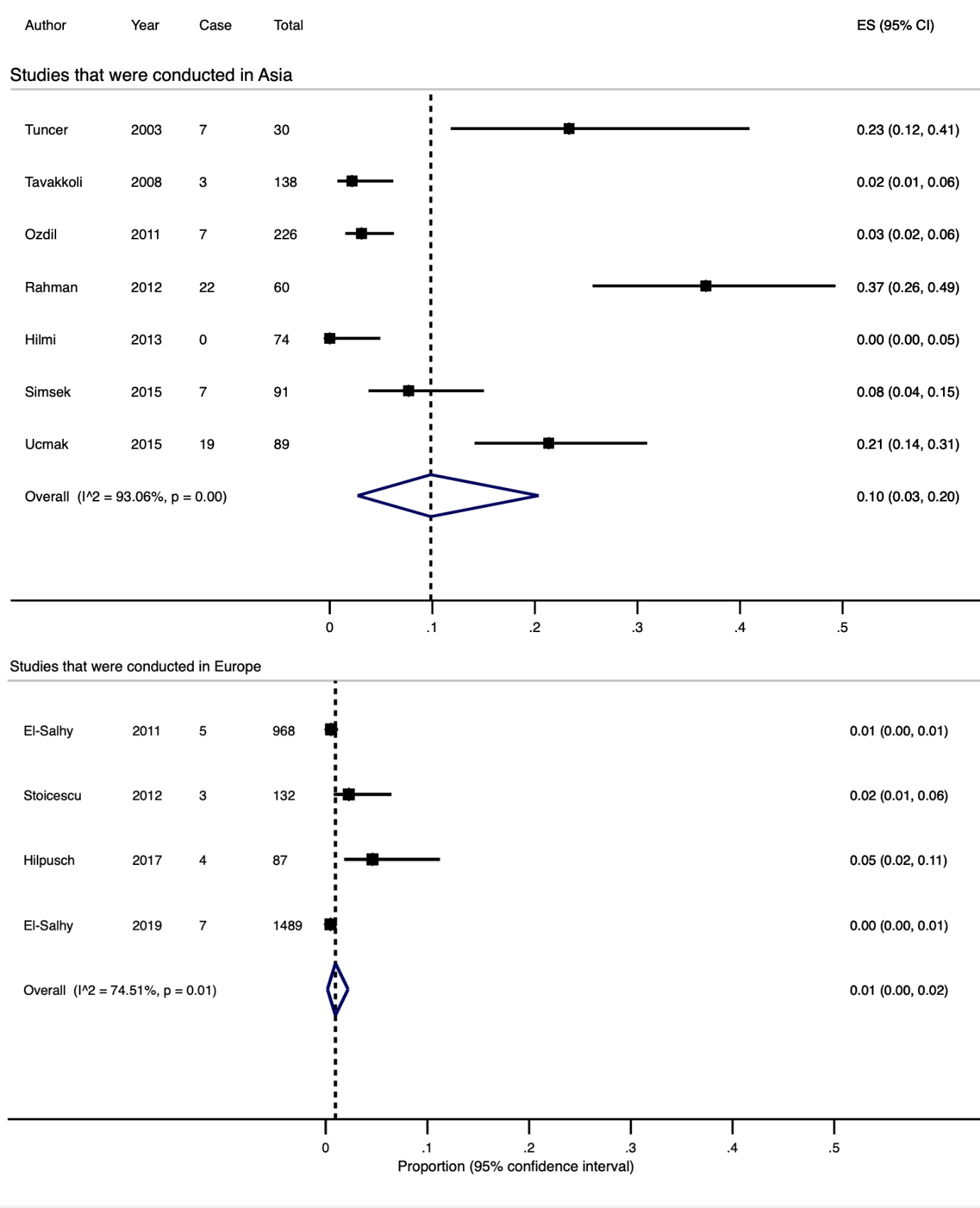
**

**Supplementary figure 20. Subgroup analyses of prevalence of lymphocytic colitis using**

1. **studies that utilised >15 and >20 lymphocytes in 100 epithelial cells to define the condition**

**
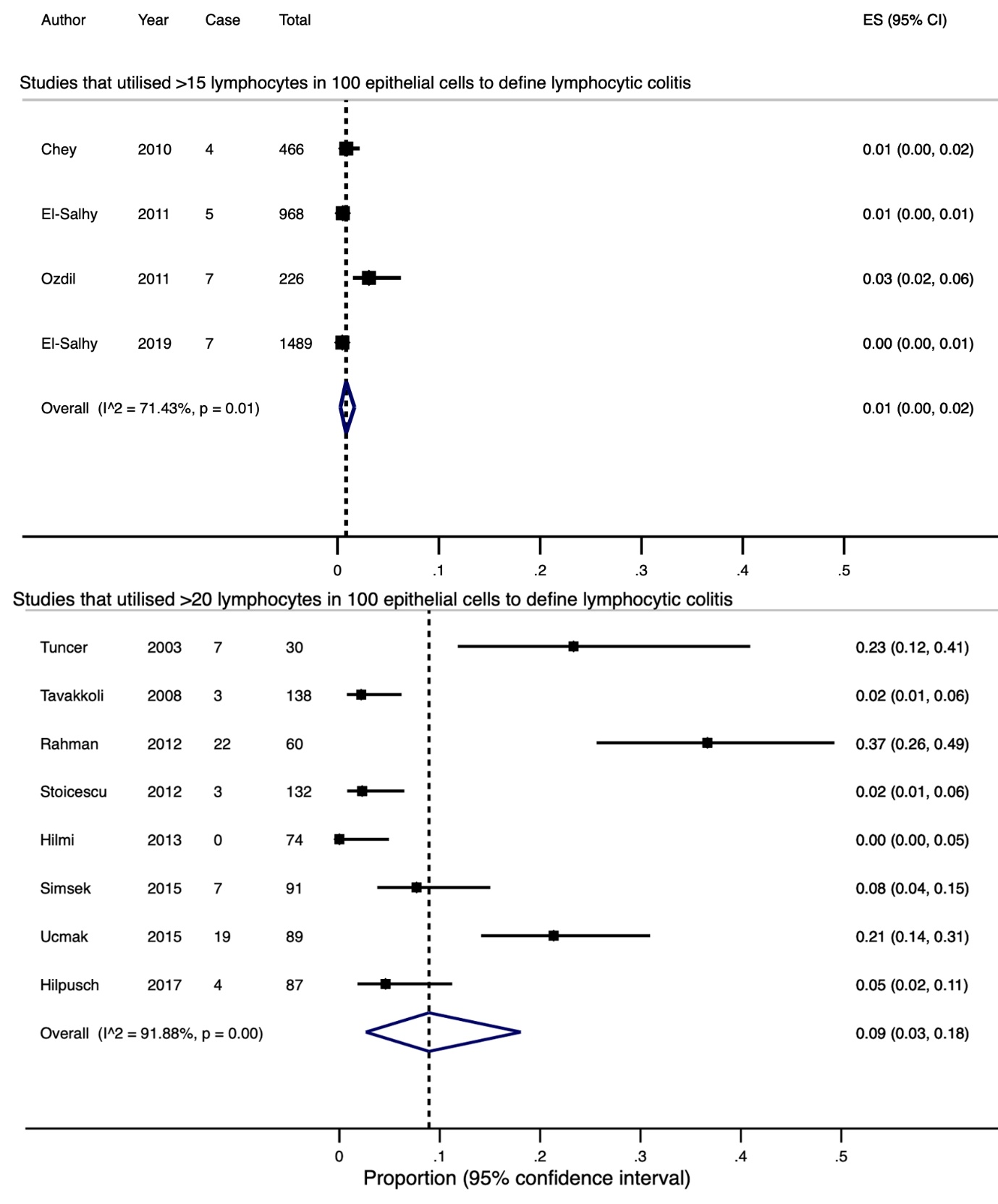
**

**Supplementary figure 21. Subgroup analyses of prevalence of collagenous colitis using**

1. **studies with a sample size ≥100, prospective studies and studies that utilised >10μm sub-epithelial collagenous band to define the condition**

**
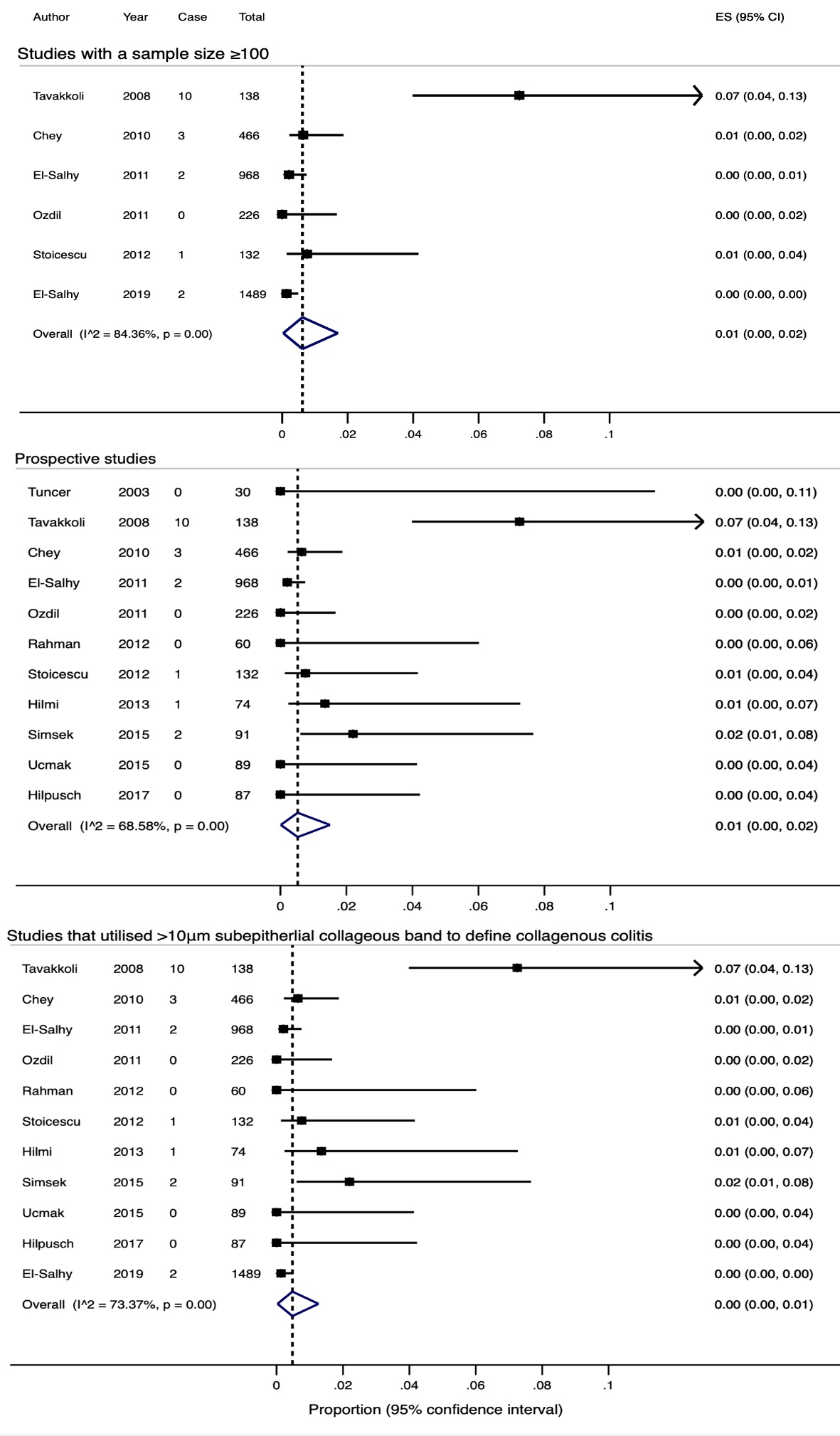
**

**Supplementary figure 21. Subgroup analyses of prevalence of collagenous colitis using**

1. **the Rome criteria**

**
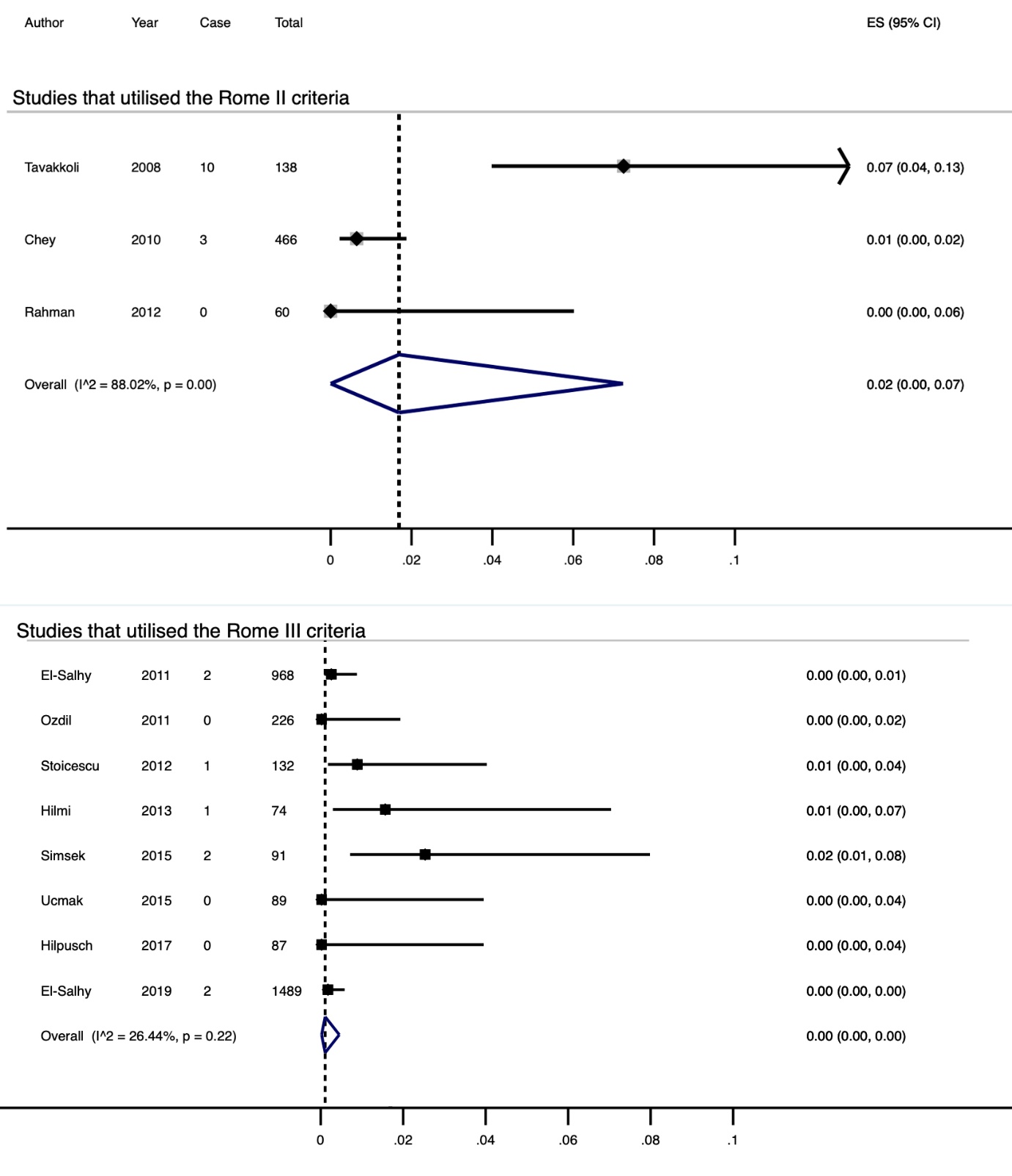
**

**Supplementary figure 21. Subgroup analyses of prevalence of collagenous colitis using**

1. **study location**

**
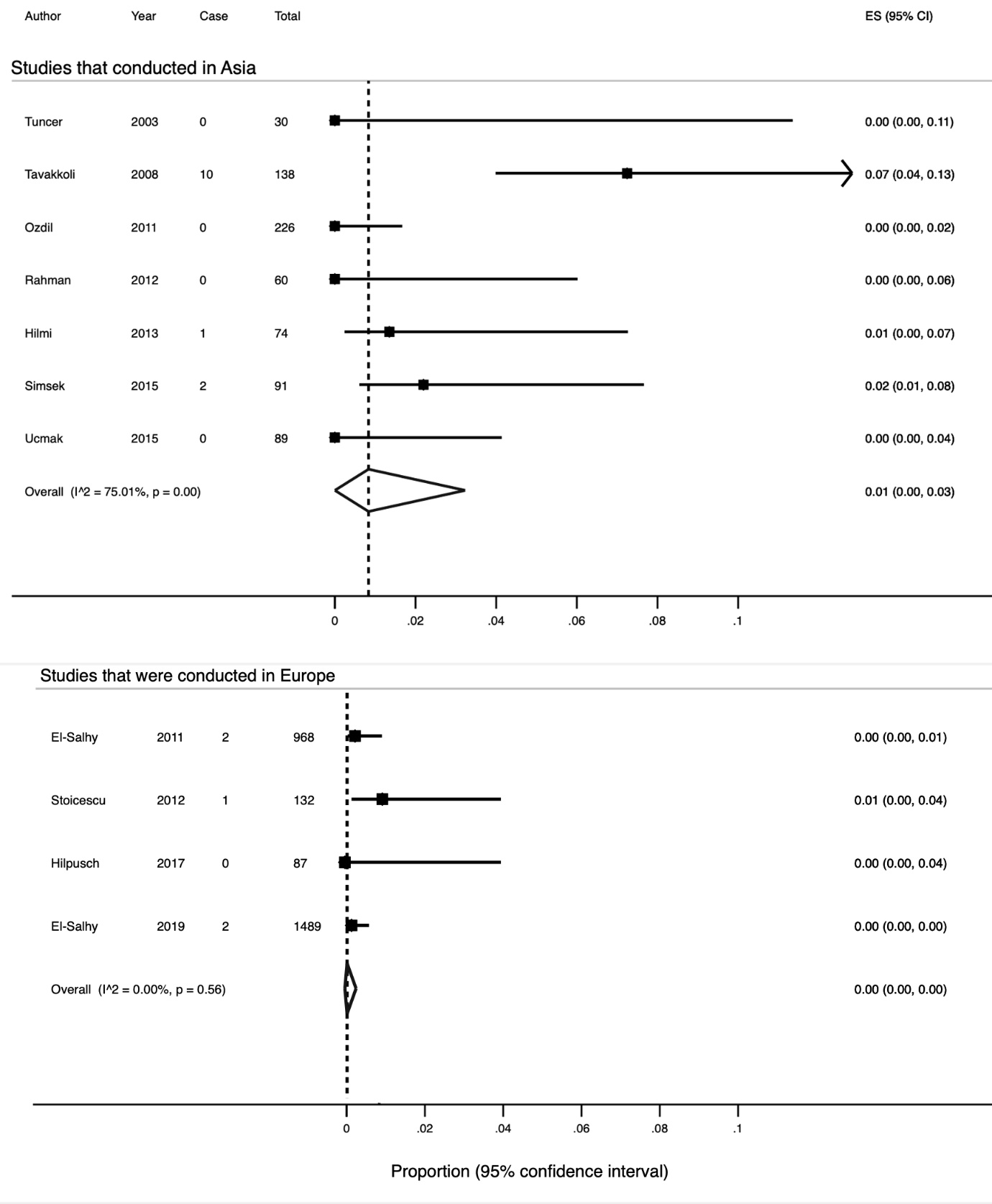
**

**Supplementary figure 22. A funnel plot indicated substantial small-study effects or publication bias among included studies of lymphocytic colitis.**

**
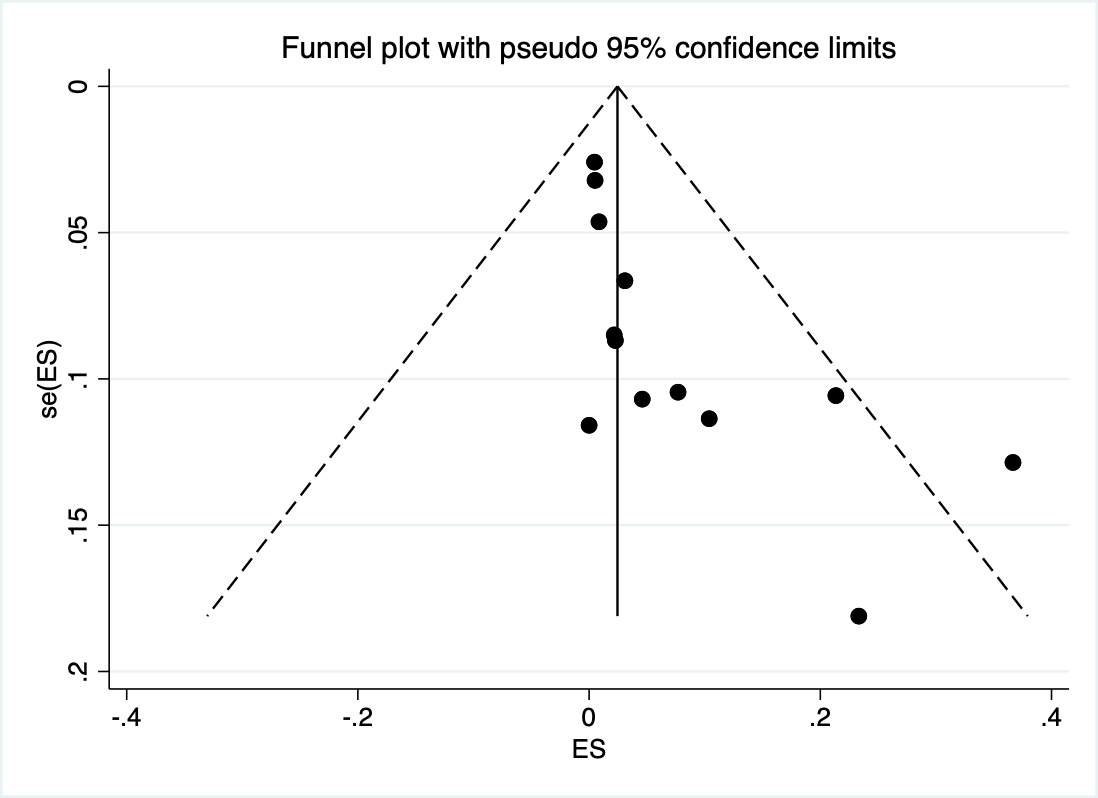
**

**Supplementary figure 23. A funnel plot indicated no small-study effects or publication bias among included studies of collagenous colitis.**

**
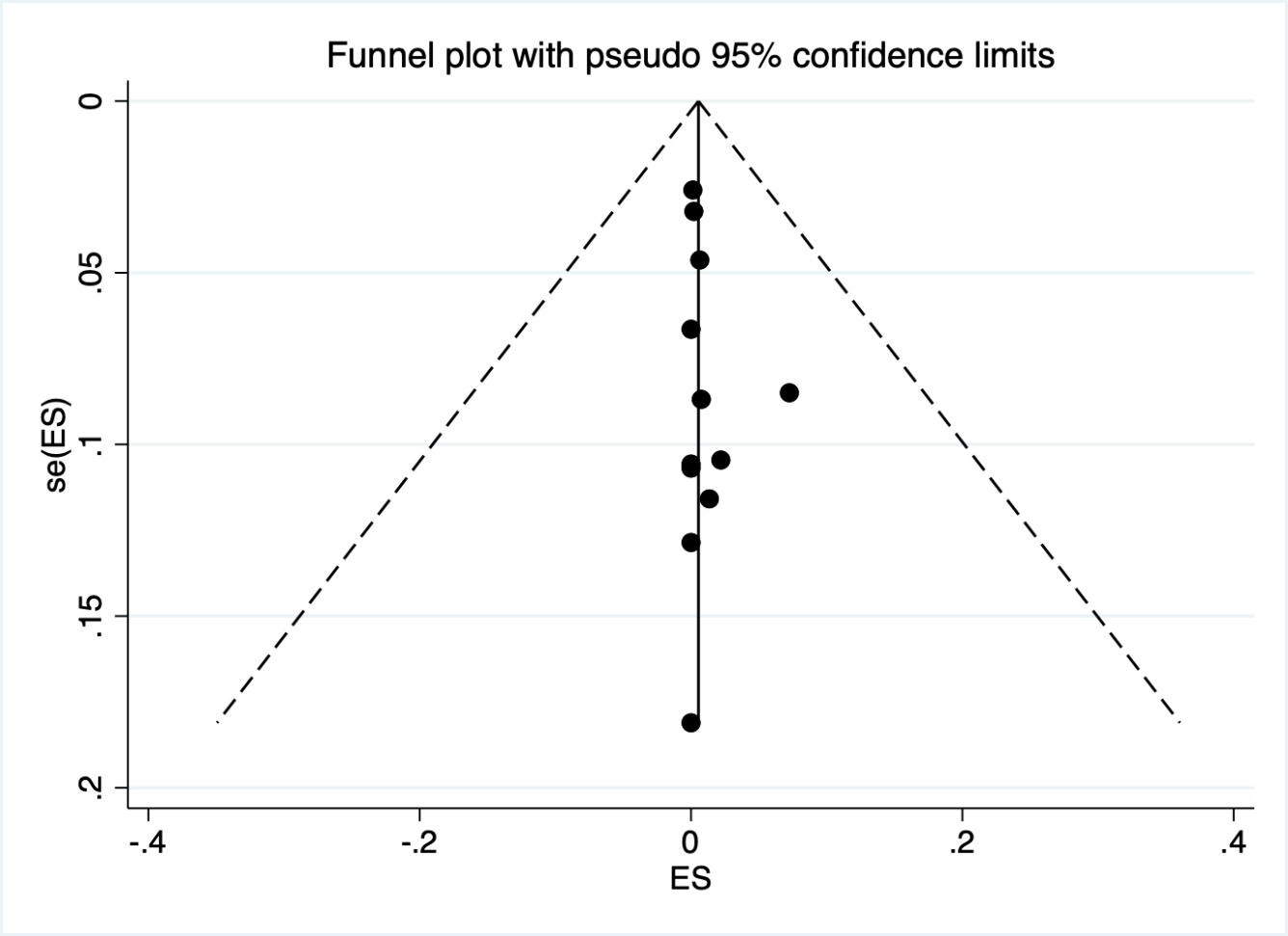
**

**Supplementary figure 24. Flow diagram showing results of literature search for PEI.**


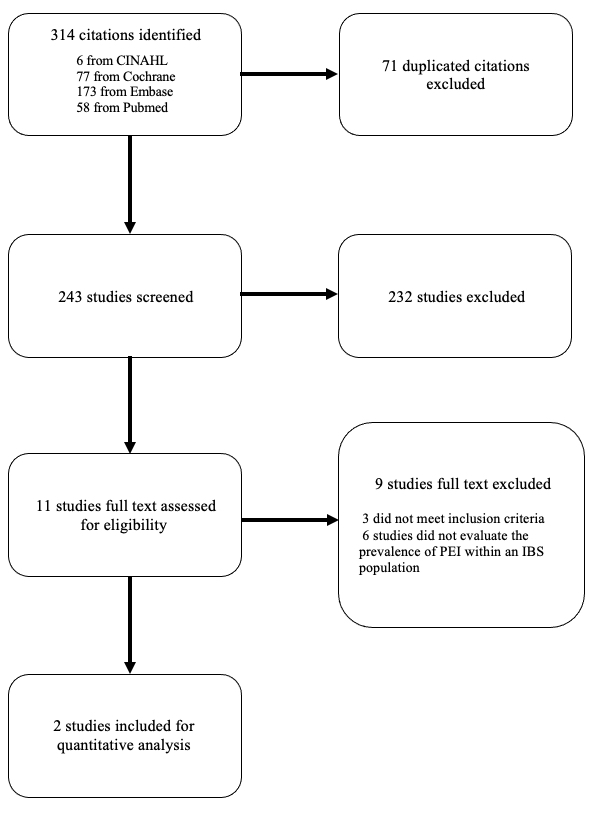


**Supplementary figure 25. Flow diagram showing results of literature search for SIBO.**


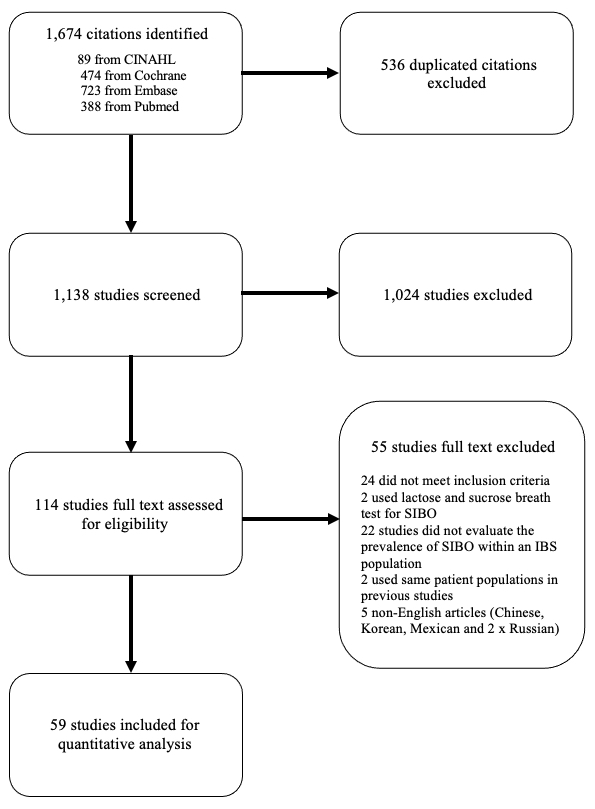


**Supplementary figure 26. Subgroup analyses of prevalence of SIBO diagnosed with lactulose breath test using**

1. **studies with a sample size ≥100**

**
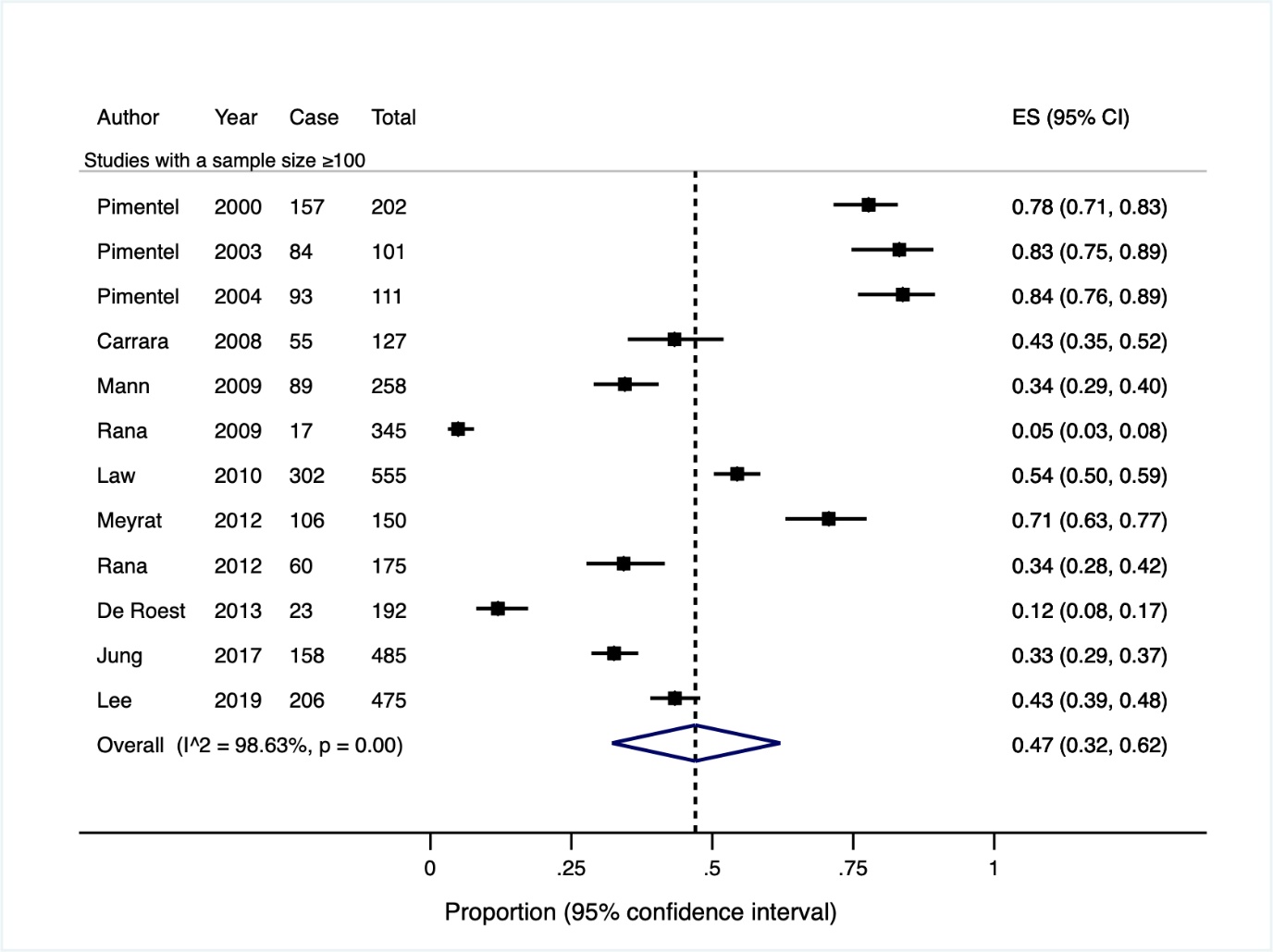
**

**Supplementary figure 26. Subgroup analyses of prevalence of SIBO diagnosed with lactulose breath test using**

1. **
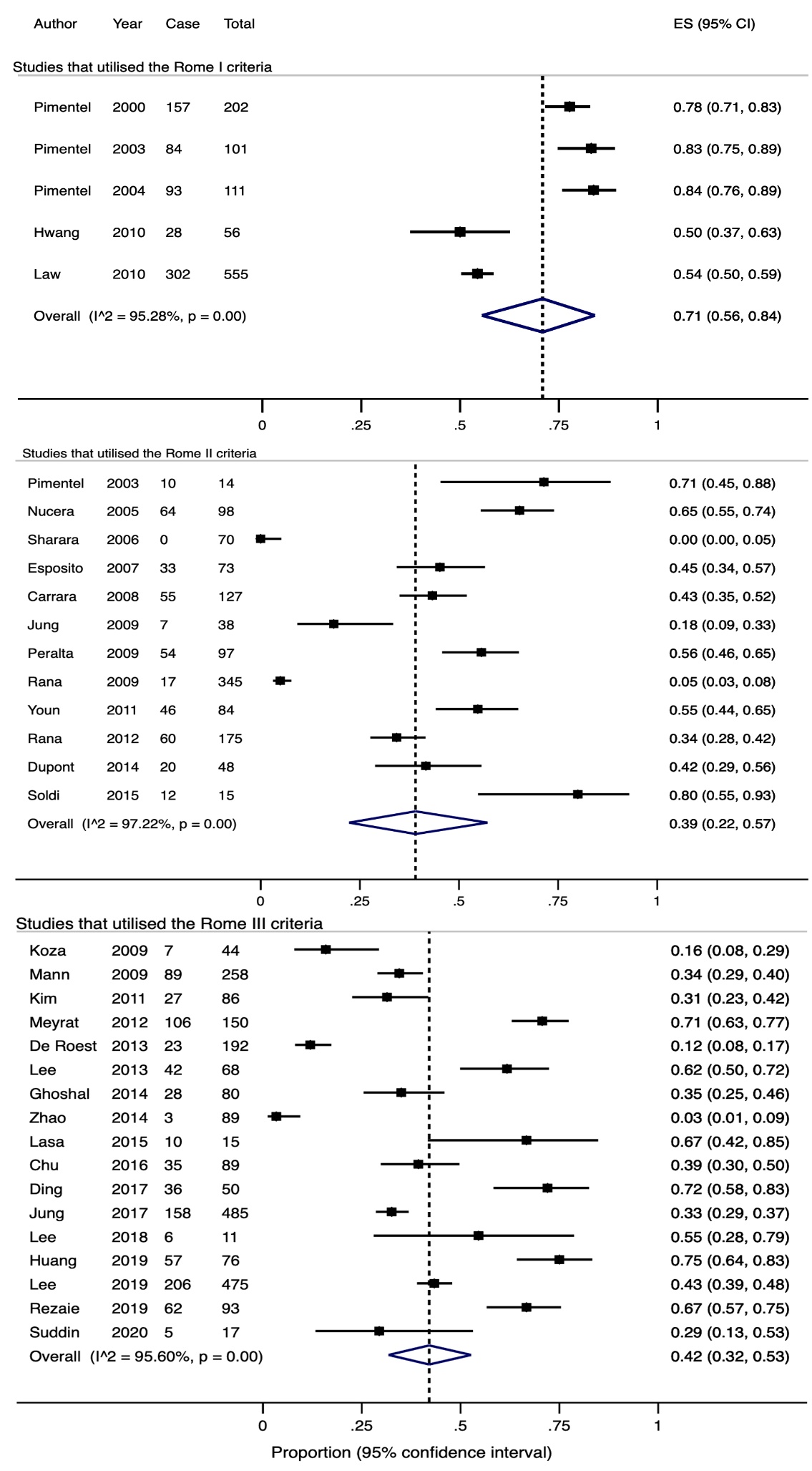
the Rome criteria**

**Supplementary figure 26. Subgroup analyses of prevalence of SIBO diagnosed with lactulose breath test using**

1. **prospective studies**

**
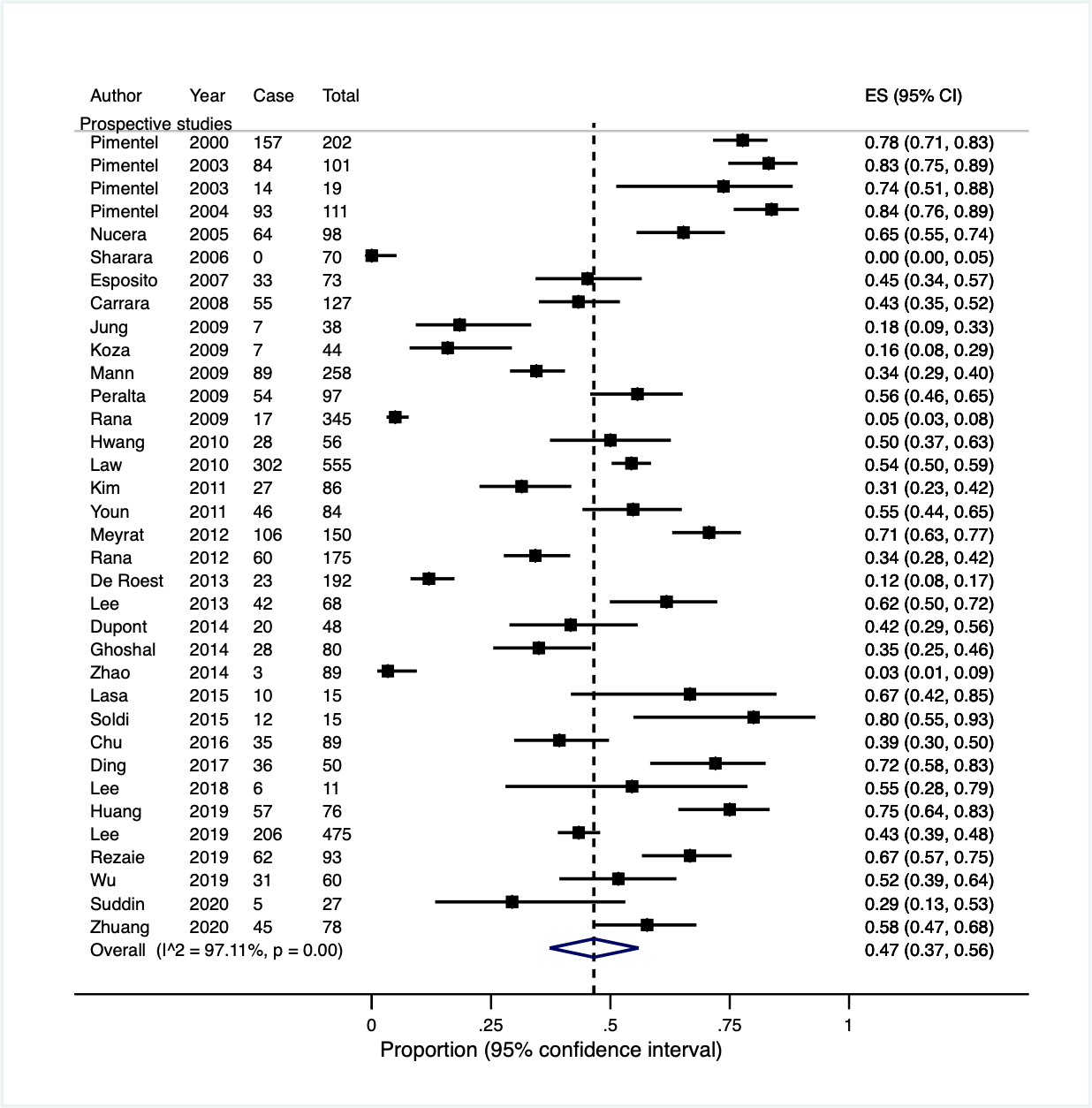
**

**Supplementary figure 26. Subgroup analyses of prevalence of SIBO diagnosed with lactulose breath test using**

1. **
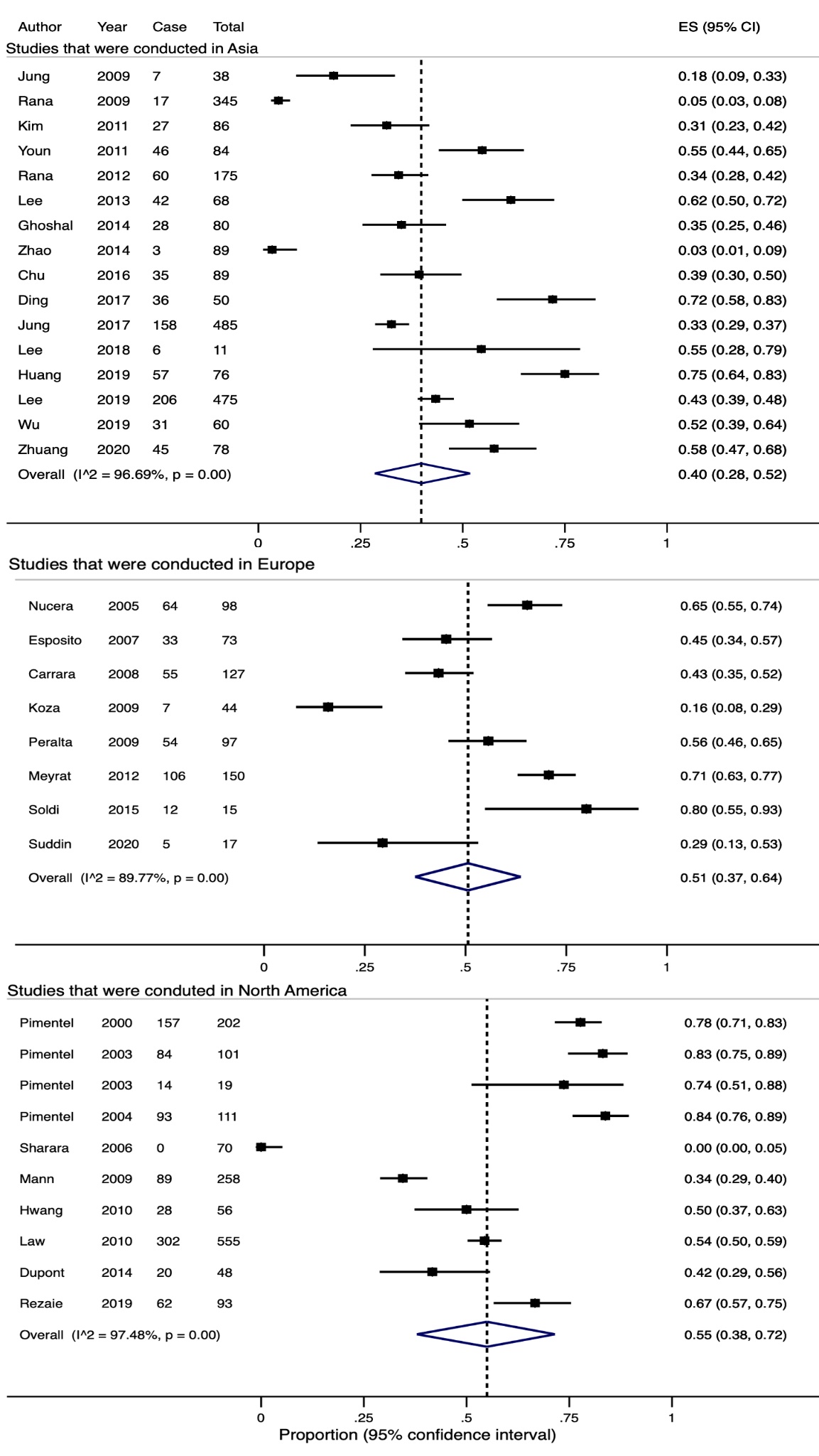
study location**

**Supplementary figure 26. Subgroup analyses of prevalence of SIBO diagnosed with lactulose breath test using**

1. **studies that utilised 10g lactulose as the test substrate**

**
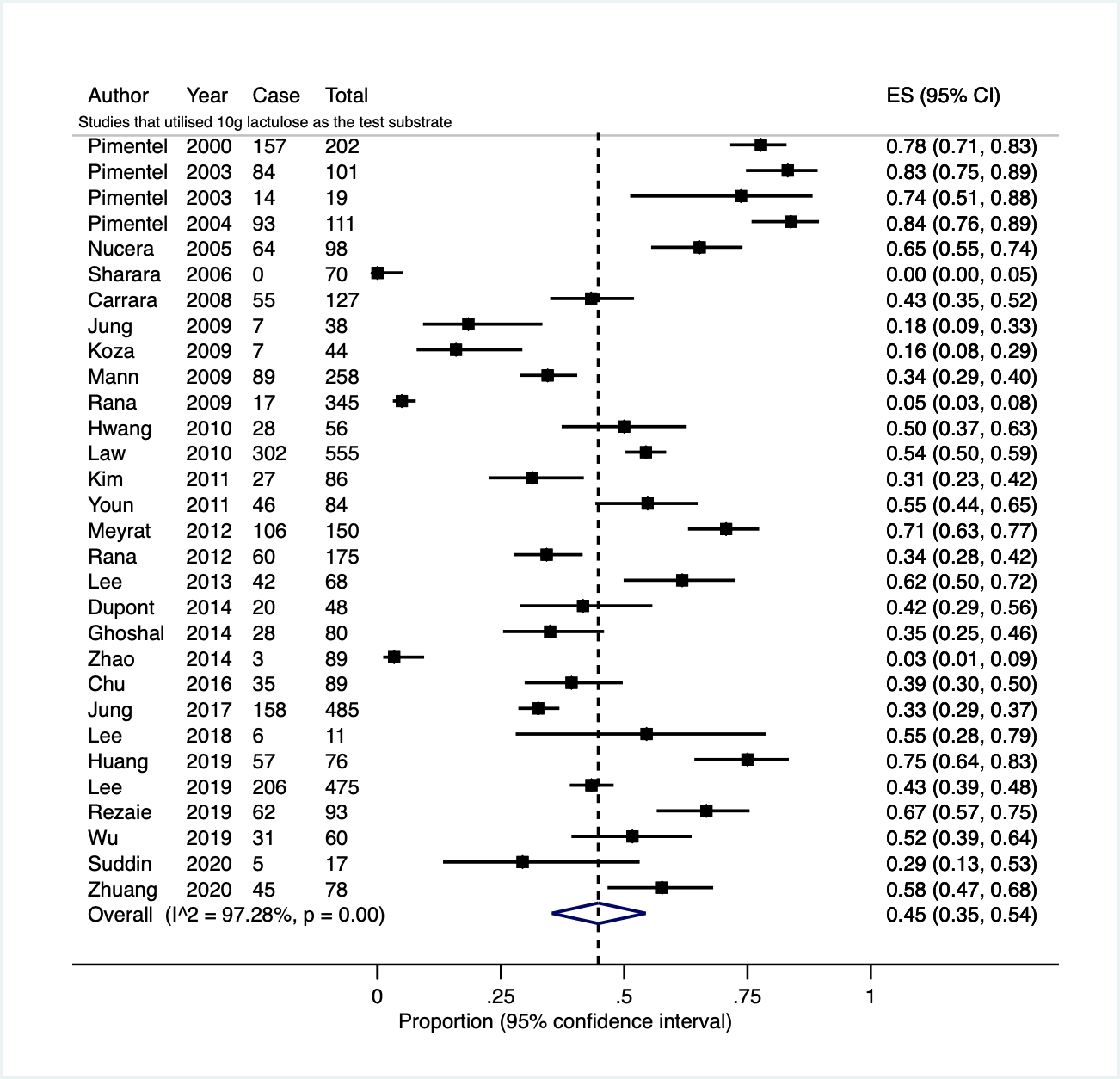
**

**Supplementary figure 27. A funnel plot indicated substantial small-study effects or publication bias among included studies of SIBO using lactulose breath testing.**


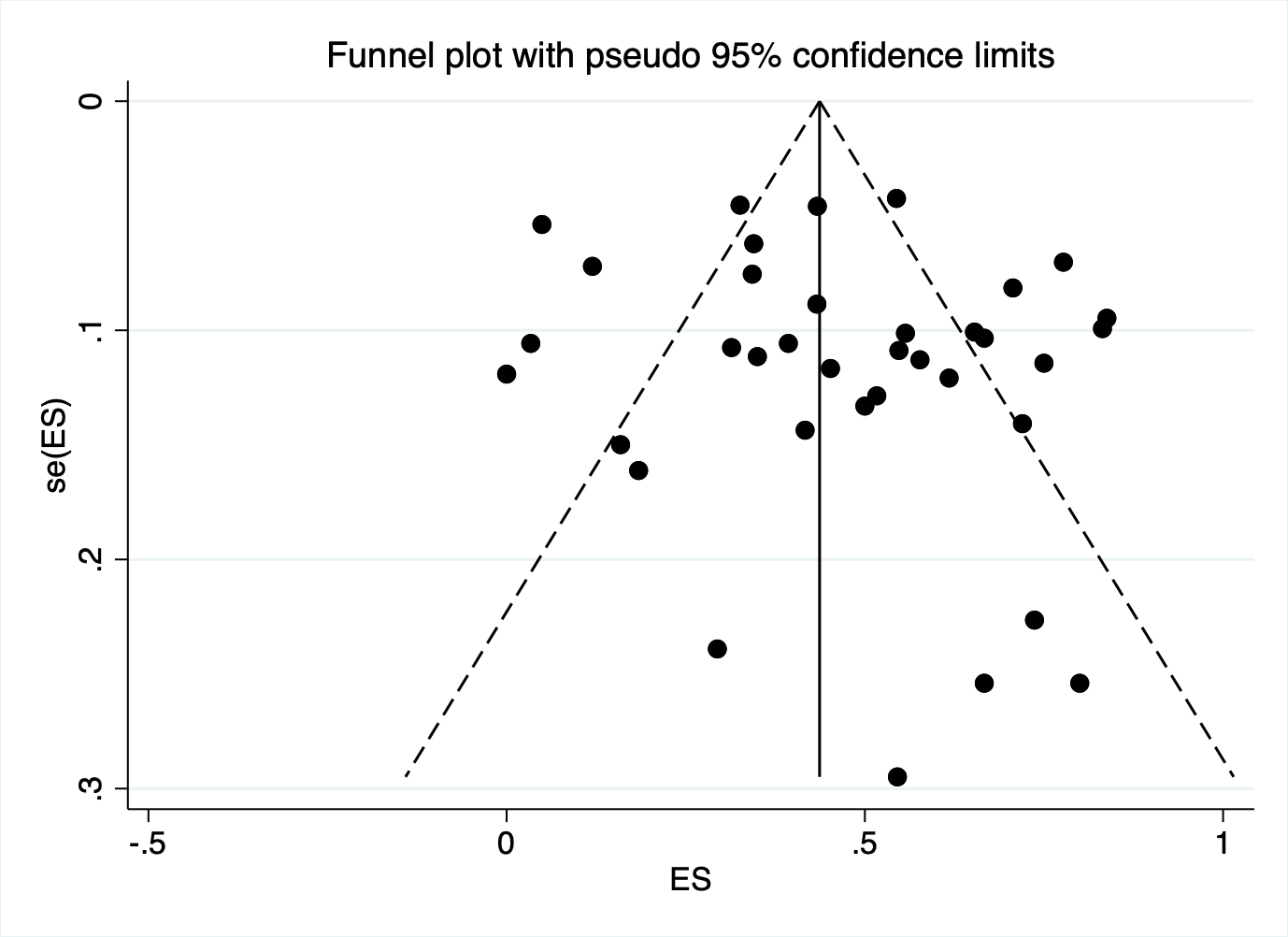


**Supplementary figure 28. A Forest plot of the 24 studies, after excluding 12 studies with high risks of bias, showing the estimated pooled prevalence of small intestinal bacterial overgrowth using lactulose hydrogen breath testing.**

**
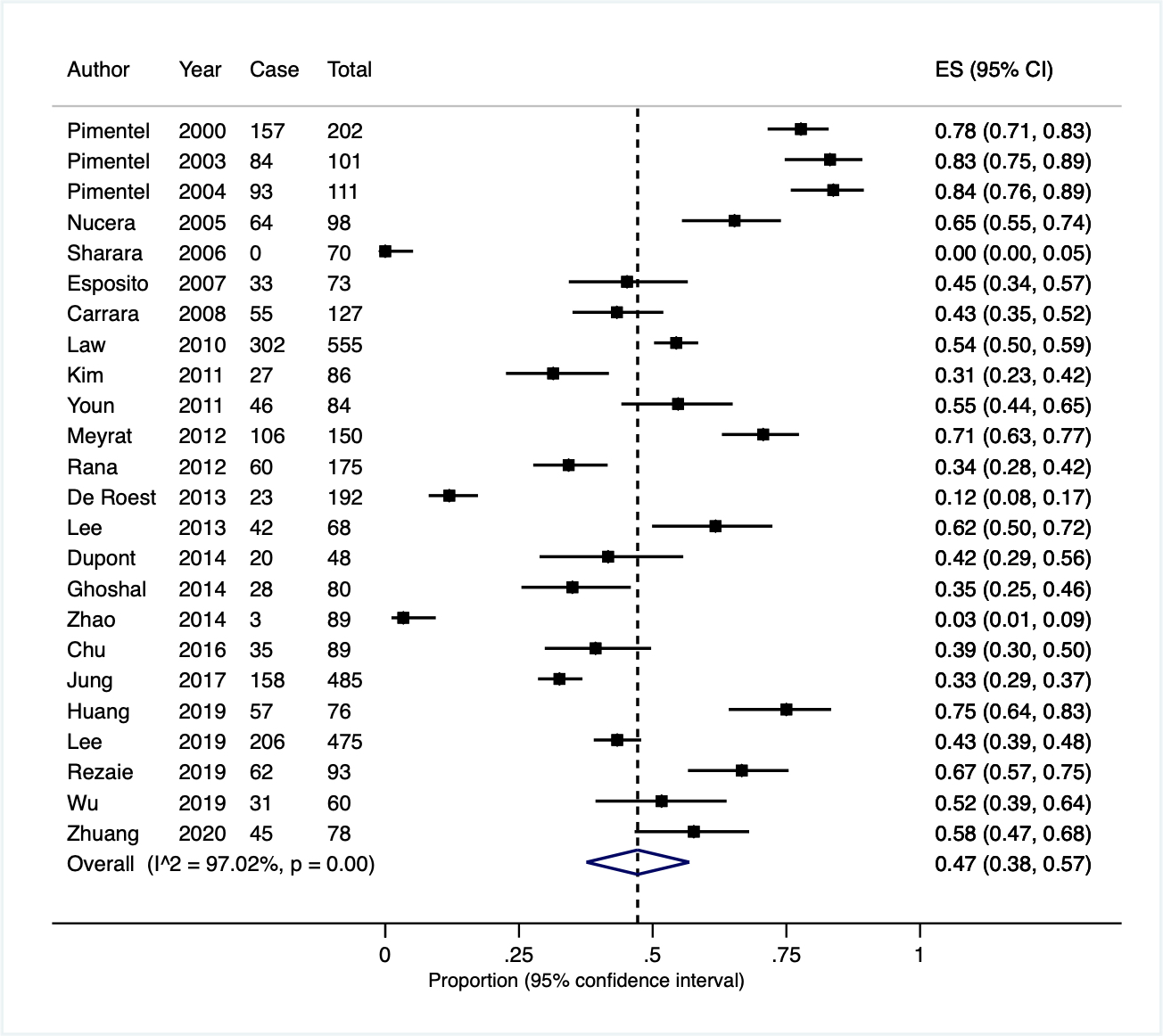
**

**Supplementary figure 29. Subgroup analyses of prevalence of SIBO diagnosed with glucose breath test using**

1. **
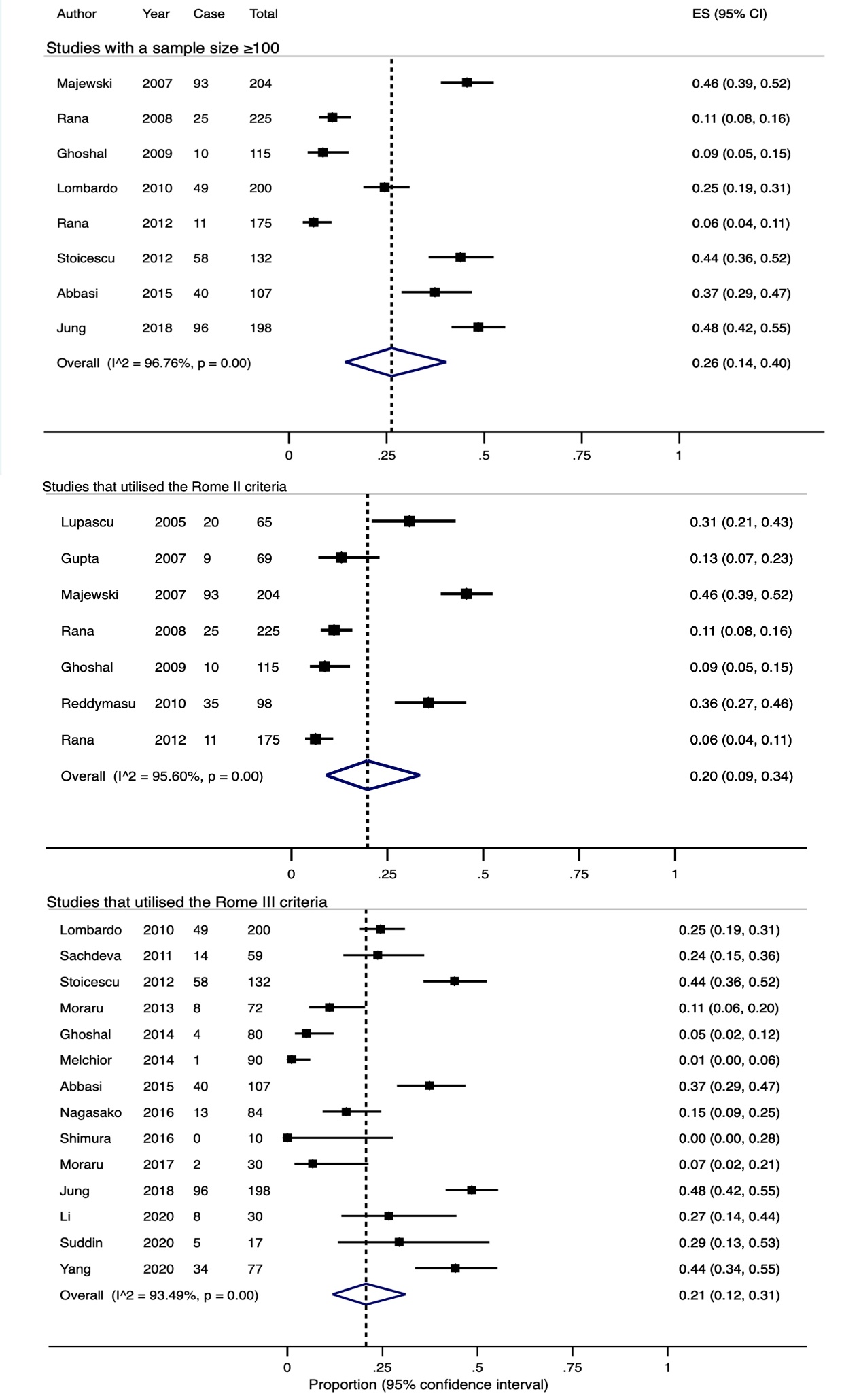
studies with a sample size ≥100 and the Rome criteria**

**Supplementary figure 29. Subgroup analyses of prevalence of SIBO diagnosed with glucose breath test using**

1. **study location**

**
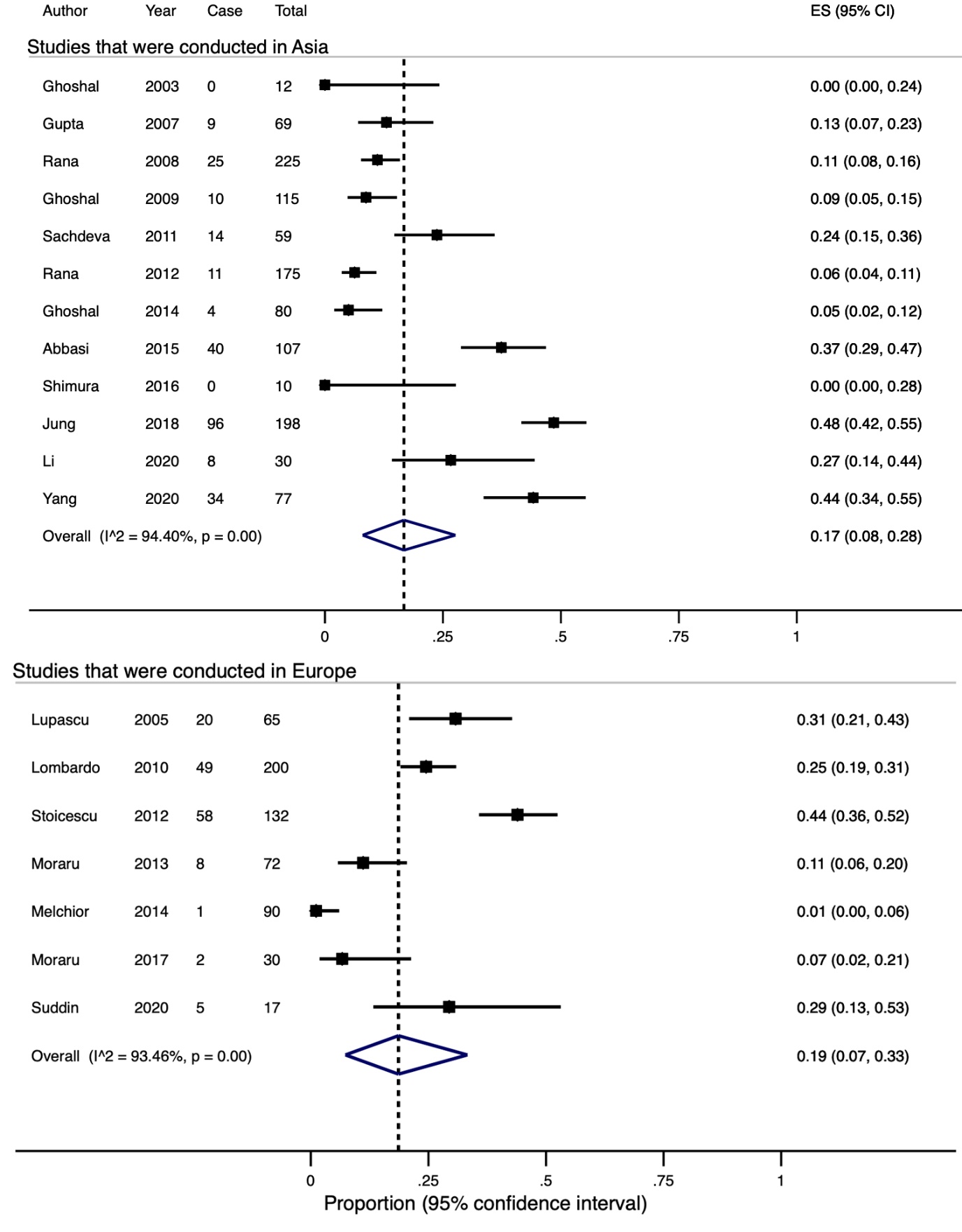
**

**Supplementary figure 29. Subgroup analyses of prevalence of SIBO diagnosed with glucose breath test using**

1. **studies that utilised 50g and 100g glucose as the test substrate**

**
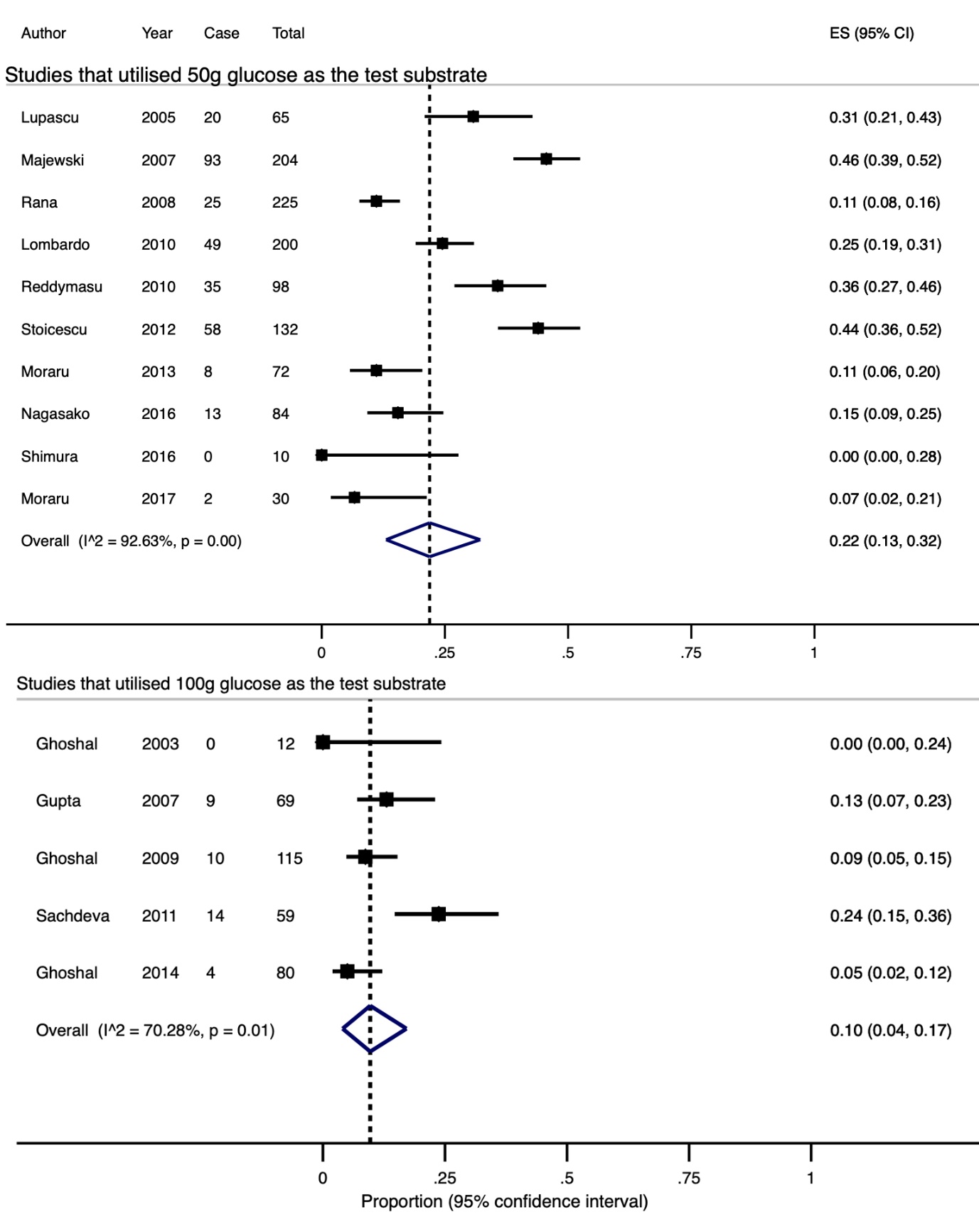
**

**Supplementary figure 30. A funnel plot indicated substantial small-study effects or publication bias among included studies of SIBO using glucose breath testing.**

**
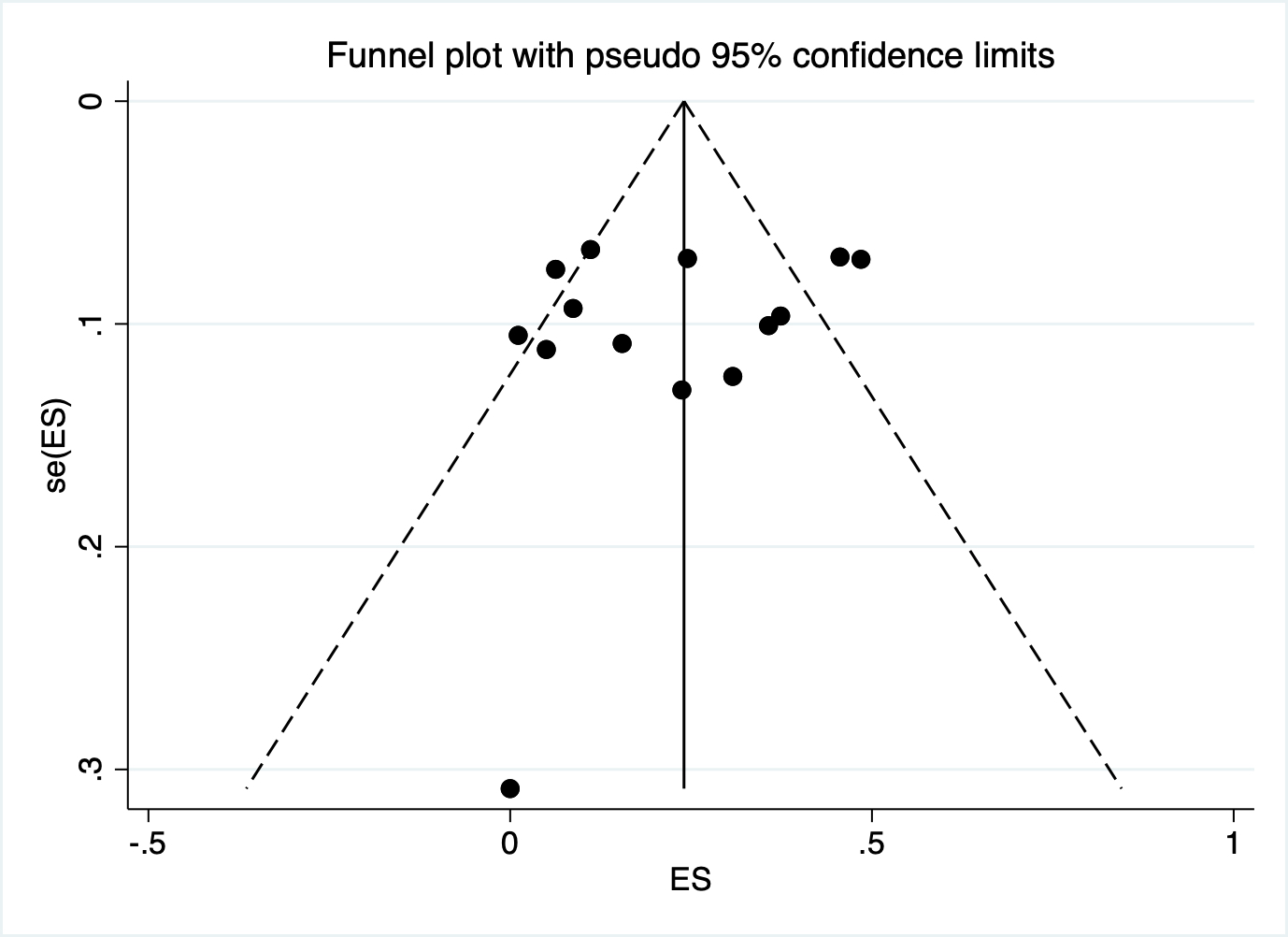
**

**Supplementary figure 31. A Forest plot of the 14 studies, after excluding eight studies with high risks of bias, showing the estimated pooled prevalence of small intestinal bacterial overgrowth using glucose hydrogen breath testing.**

**
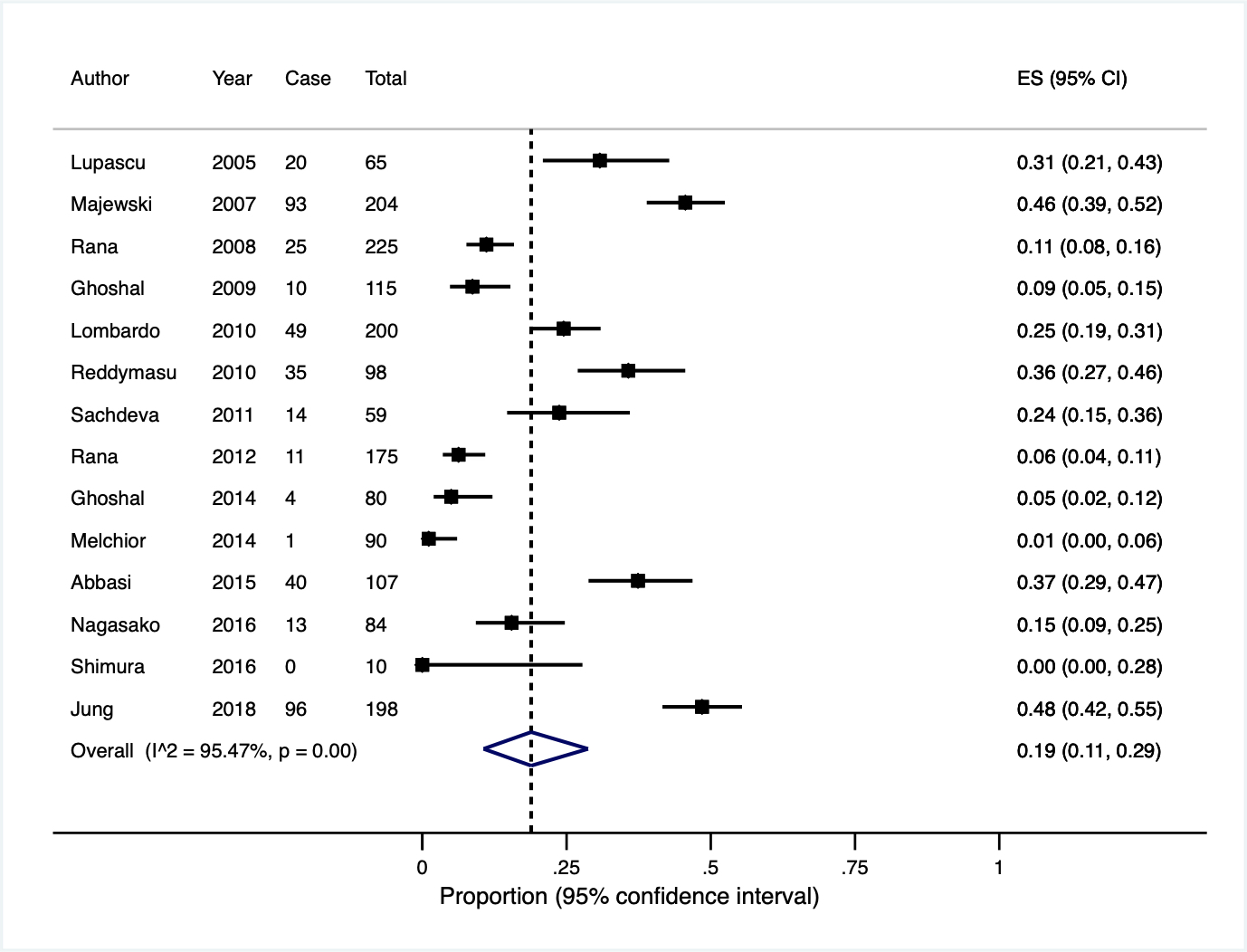
**

**Supplementary figure 32. A Forest plot of the seven studies showing the estimated pooled prevalence of SIBO, based on examination of the small bowel aspiration.**

**
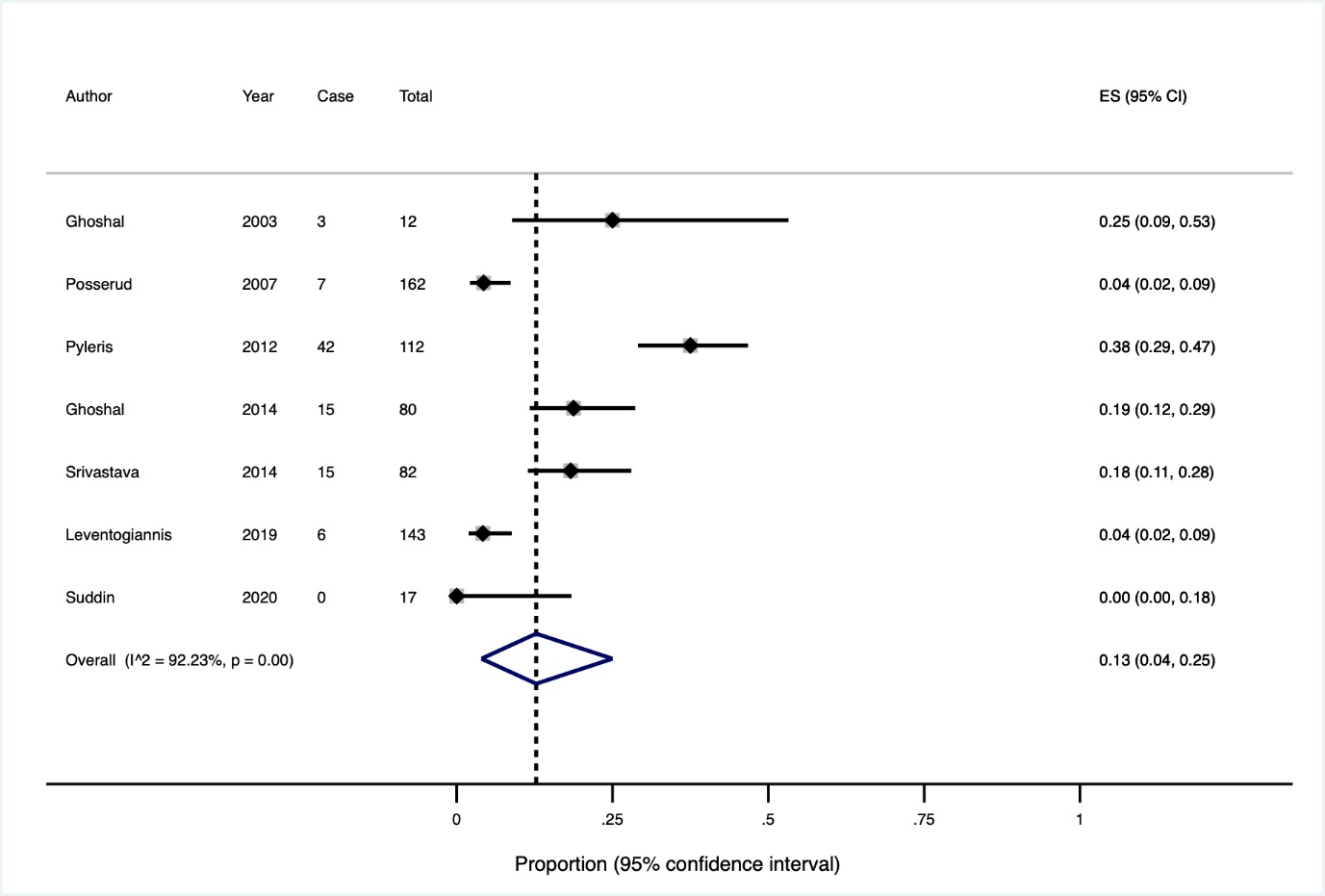
**

**Supplementary
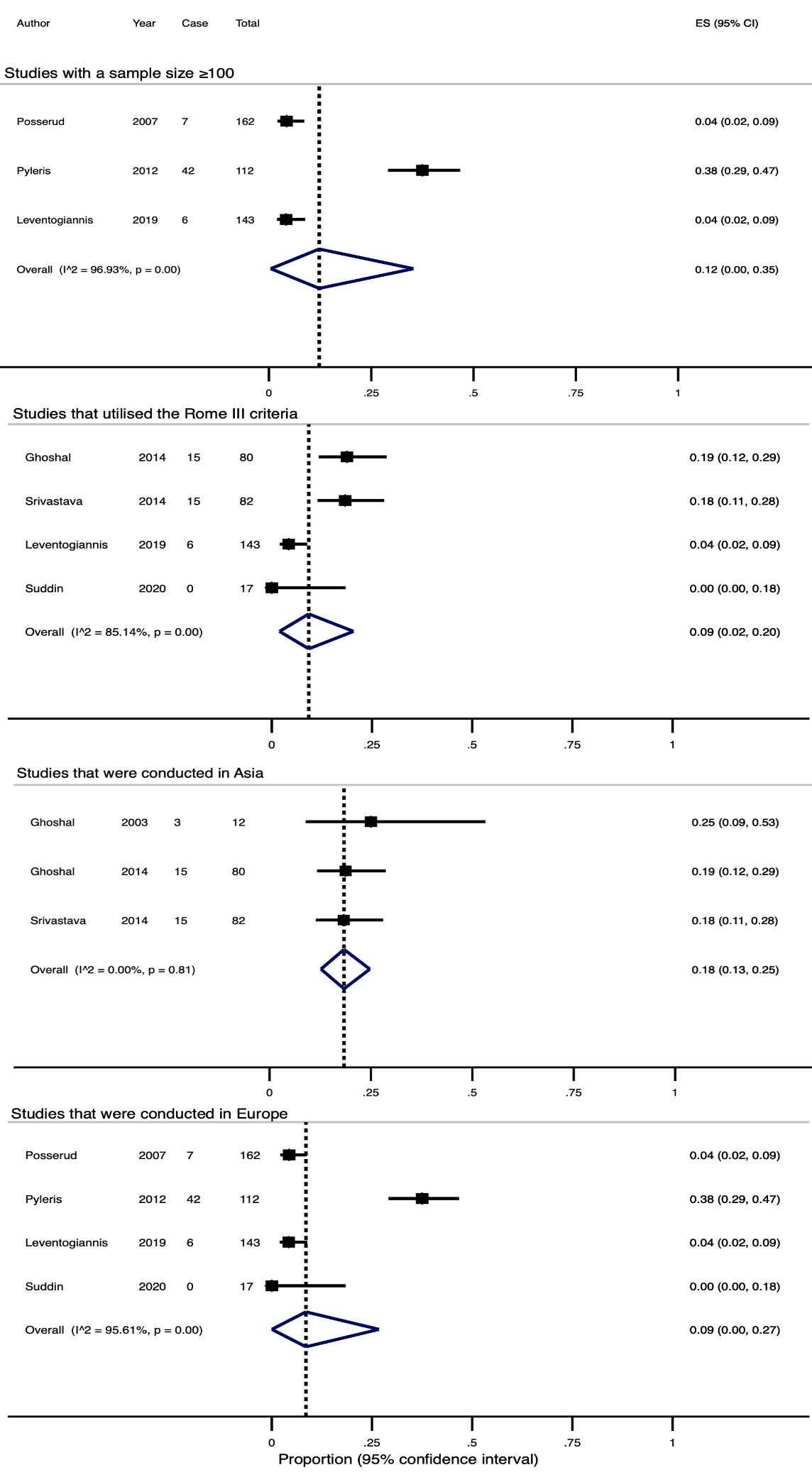
figure 33. Subgroup analyses of prevalence of SIBO diagnosed with small bowel aspiration.**

**Supplementary figure 34. A Forest plot of the five studies, after excluding two studies with high risks of bias, showing the estimated pooled prevalence of small intestinal bacterial overgrowth using small bowel aspirate.**

**
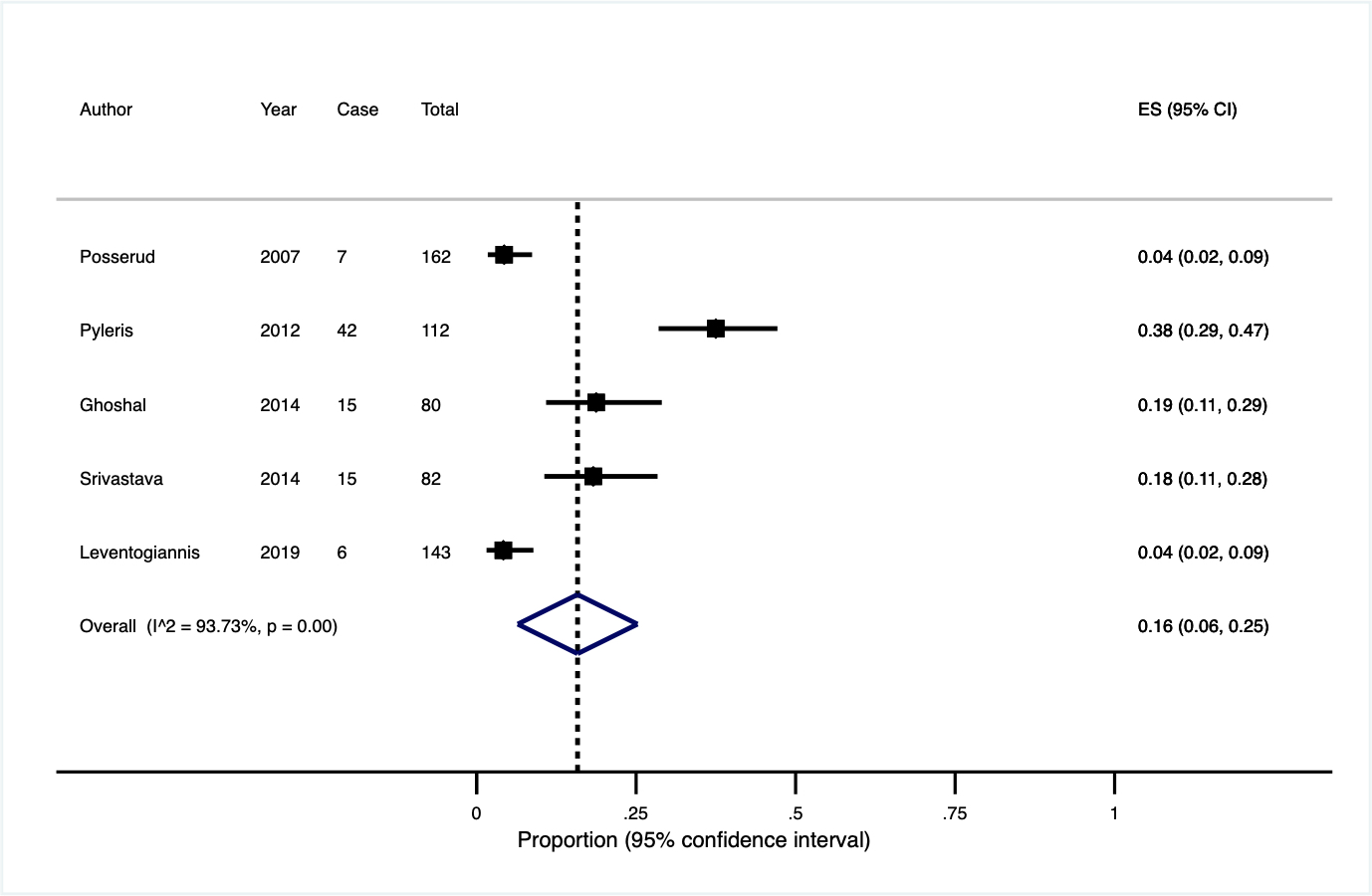
**

**Supplementary table 1. Appraisal of studies evaluating prevalence of BAD**

| Author (Year) | Did the study address a clearly focused issue? | Was the cohort recruited in an acceptable way? | Was the exposure accurately measured to minimise bias? | Was the outcome accurately measured to minimise bias? | Have the authors identified all important confounding factors? | Have they taken account of the confounding factors in the design and/or analysis? | Do you believe the results? | Can the results be applied to the local population? | Do the results of this study fit with other available evidence? | What are the implications of this study for practice? | Total score |
| --- | --- | --- | --- | --- | --- | --- | --- | --- | --- | --- | --- |
|  |  |  |  |  |  |  |  |  |  |  |  |
|  |  |  |  |  |  |  |  |  |  |  |  |
| Galatola (1992) | 1 | 1 | 1 | 1 | 1 | 1 | 1 | 1 | 1 | 1 | 10 |
| Sinha (1998) | 1 | 1 | 1 | 1 | 1 | 1 | 1 | 0 | 1 | 1 | 9 |
| Smith (2000) | 1 | 1 | 1 | 1 | 1 | 1 | 1 | 1 | 1 | 1 | 10 |
| Fernandez-Banares (2007) | 1 | 1 | 1 | 1 | 1 | 1 | 0 | 0 | 1 | 1 | 8 |
| Gracie (2012) | 1 | 1 | 1 | 1 | 1 | 1 | 1 | 1 | 1 | 1 | 10 |
| Bajor (2014) | 1 | 1 | 1 | 1 | 1 | 1 | 1 | 1 | 1 | 1 | 10 |
| Camilleri (2014) | 1 | 1 | 1 | 1 | 1 | 1 | 1 | 0 | 1 | 1 | 9 |
| Aziz (2015) | 1 | 1 | 1 | 1 | 1 | 1 | 1 | 1 | 1 | 1 | 10 |
| Fernandez-Banares (2015) | 1 | 0 | 1 | 1 | 1 | 1 | 0 | 0 | 1 | 1 | 7 |
| Dior (2016) | 1 | 1 | 1 | 1 | 1 | 1 | 0 | 0 | 1 | 1 | 8 |
| Donato (2018) | 1 | 0 | 0 | 1 | 0 | 0 | 1 | 0 | 1 | 1 | 5 |
| Vijayvargiya (2018) | 1 | 1 | 1 | 1 | 1 | 1 | 1 | 1 | 1 | 1 | 10 |
| Jeffery (2020) | 1 | 1 | 1 | 1 | 1 | 1 | 1 | 0 | 1 | 1 | 9 |
| Shiha et al. (2020) | 1 | 1 | 1 | 1 | 1 | 1 | 1 | 1 | 1 | 1 | 10 |
| Vijayvargiya (2020) | 1 | 1 | 1 | 1 | 1 | 1 | 1 | 0 | 1 | 1 | 9 |

**Supplementary table 2. Appraisal of studies evaluating prevalence of CM**

| Author (Year) | Did the study address a clearly focused issue? | Was the cohort recruited in an acceptable way? | Was the exposure accurately measured to minimise bias? | Was the outcome accurately measured to minimise bias? | Have the authors identified all important confounding factors? | Have they taken account of the confounding factors in the design and/or analysis? | Do you believe the results? | Can the results be applied to the local population? | Do the results of this study fit with other available evidence? | What are the implications of this study for practice? | Total score |
| --- | --- | --- | --- | --- | --- | --- | --- | --- | --- | --- | --- |
|  |  |  |  |  |  |  |  |  |  |  |  |
|  |  |  |  |  |  |  |  |  |  |  |  |
| Neils (1990) | 1 | 1 | 0 | 1 | 0 | 0 | 1 | 0 | 1 | 1 | 6 |
| Vernia (1995) | 1 | 1 | 1 | 1 | 0 | 0 | 1 | 1 | 1 | 1 | 8 |
| Böhmer (1996) | 1 | 1 | 0 | 1 | 1 | 1 | 1 | 0 | 1 | 1 | 8 |
| Hamm (1999) | 1 | 1 | 0 | 1 | 1 | 1 | 1 | 1 | 1 | 1 | 9 |
| Goldstein (2000) | 1 | 1 | 1 | 1 | 0 | 0 | 1 | 1 | 1 | 1 | 8 |
| Parker (2001) | 1 | 1 | 0 | 1 | 1 | 1 | 1 | 1 | 1 | 1 | 9 |
| Vernia (2001) | 1 | 1 | 0 | 1 | 1 | 1 | 1 | 1 | 1 | 1 | 9 |
| Pimentel (2003) | 1 | 1 | 1 | 1 | 1 | 1 | 1 | 0 | 1 | 1 | 9 |
| Farup (2004) | 1 | 0 | 0 | 1 | 1 | 1 | 1 | 1 | 1 | 1 | 8 |
| Vernia (2004) | 1 | 0 | 0 | 1 | 1 | 1 | 1 | 1 | 1 | 1 | 8 |
| Nucera (2005) | 1 | 1 | 1 | 1 | 1 | 1 | 1 | 0 | 1 | 1 | 9 |
| Bernardes-Silva (2007) | 1 | 1 | 1 | 1 | 1 | 1 | 1 | 0 | 1 | 1 | 9 |
| Fernandez-Banares (2007) | 1 | 1 | 0 | 1 | 0 | 0 | 1 | 0 | 0 | 1 | 5 |
| Gupta (2007) | 1 | 1 | 0 | 0 | 1 | 1 | 1 | 1 | 1 | 1 | 8 |
| Choi (2008) | 1 | 1 | 1 | 1 | 1 | 1 | 1 | 0 | 1 | 1 | 9 |
| Skoog (2008) | 1 | 1 | 0 | 1 | 1 | 1 | 1 | 0 | 1 | 1 | 8 |
| Author (Year) | Did the study address a clearly focused issue? | Was the cohort recruited in an acceptable way? | Was the exposure accurately measured to minimise bias? | Was the outcome accurately measured to minimise bias? | Have the authors identified all important confounding factors? | Have they taken account of the confounding factors in the design and/or analysis? | Do you believe the results? | Can the results be applied to the local population? | Do the results of this study fit with other available evidence? | What are the implications of this study for practice? | Total score |
| Corlew-Roath (2009) | 1 | 1 | 0 | 0 | 0 | 0 | 1 | 1 | 1 | 1 | 6 |
| Ghoshal (2009) | 1 | 1 | 1 | 0 | 1 | 1 | 1 | 1 | 1 | 1 | 9 |
| Yakoob (2011) | 1 | 1 | 1 | 0 | 1 | 1 | 0 | 1 | 1 | 1 | 8 |
| Kumar (2012) | 1 | 1 | 1 | 1 | 1 | 1 | 1 | 1 | 1 | 1 | 10 |
| Berg (2013) | 1 | 1 | 0 | 1 | 0 | 0 | 1 | 1 | 1 | 1 | 7 |
| De Roest (2013) | 1 | 1 | 0 | 1 | 0 | 1 | 1 | 1 | 1 | 1 | 8 |
| Ghoshal (2013) | 1 | 1 | 0 | 1 | 1 | 1 | 1 | 1 | 1 | 1 | 9 |
| Yang (2013) | 1 | 1 | 0 | 1 | 1 | 1 | 1 | 0 | 1 | 1 | 8 |
| Zhu (2013) | 1 | 1 | 0 | 1 | 1 | 1 | 1 | 1 | 1 | 1 | 9 |
| Dainese (2014) | 1 | 1 | 0 | 1 | 1 | 1 | 1 | 0 | 1 | 1 | 8 |
| Goebel-Stengel (2014) | 1 | 1 | 0 | 1 | 1 | 1 | 1 | 0 | 1 | 1 | 8 |
| Jafari (2014) | 1 | 0 | 0 | 0 | 0 | 0 | 1 | 1 | 1 | 1 | 5 |
| Melchoir (2014) | 1 | 1 | 1 | 1 | 1 | 1 | 1 | 0 | 1 | 1 | 9 |
| Sharma (2014) | 1 | 1 | 0 | 1 | 0 | 0 | 1 | 0 | 1 | 1 | 6 |
| Yang (2014) | 1 | 1 | 0 | 1 | 1 | 1 | 1 | 0 | 1 | 1 | 8 |
| Yao (2014) | 1 | 1 | 0 | 1 | 0 | 0 | 1 | 0 | 1 | 1 | 6 |
| Wang (2015) | 1 | 1 | 1 | 1 | 1 | 1 | 1 | 1 | 1 | 1 | 10 |
| Dabak (2017) | 1 | 1 | 0 | 1 | 1 | 1 | 1 | 0 | 1 | 1 | 8 |
| Xiong (2017) | 1 | 1 | 0 | 1 | 1 | 1 | 1 | 0 | 1 | 1 | 8 |

| Author (Year) | Did the study address a clearly focused issue? | Was the cohort recruited in an acceptable way? | Was the exposure accurately measured to minimise bias? | Was the outcome accurately measured to minimise bias? | Have the authors identified all important confounding factors? | Have they taken account of the confounding factors in the design and/or analysis? | Do you believe the results? | Can the results be applied to the local population? | Do the results of this study fit with other available evidence? | What are the implications of this study for practice? | Total score |
| --- | --- | --- | --- | --- | --- | --- | --- | --- | --- | --- | --- |
| Almazar (2018) | 1 | 1 | 0 | 1 | 1 | 1 | 0 | 0 | 1 | 1 | 7 |
| Jung (2018) | 1 | 1 | 1 | 1 | 1 | 1 | 1 | 0 | 1 | 1 | 9 |
| Melchoir (2019) | 1 | 1 | 1 | 1 | 1 | 1 | 1 | 0 | 1 | 1 | 9 |
| Mack (2020) | 1 | 1 | 1 | 1 | 1 | 1 | 0 | 0 | 0 | 1 | 7 |

**Supplementary table 3. Appraisal of studies evaluating prevalence of MC**

| Author (Year) | Did the study address a clearly focused issue? | Was the cohort recruited in an acceptable way? | Was the exposure accurately measured to minimise bias? | Was the outcome accurately measured to minimise bias? | Have the authors identified all important confounding factors? | Have they taken account of the confounding factors in the design and/or analysis? | Do you believe the results? | Can the results be applied to the local population? | Do the results of this study fit with other available evidence? | What are the implications of this study for practice? | Total score |
| --- | --- | --- | --- | --- | --- | --- | --- | --- | --- | --- | --- |
|  |  |  |  |  |  |  |  |  |  |  |  |
|  |  |  |  |  |  |  |  |  |  |  |  |
| Chadwick (2002) | 1 | 1 | 1 | 1 | 1 | 1 | 1 | 0 | 1 | 1 | 9 |
| Tuncer (2003) | 1 | 1 | 1 | 1 | 1 | 1 | 0 | 0 | 1 | 1 | 8 |
| Tavakkoli (2008) | 1 | 1 | 1 | 1 | 1 | 1 | 1 | 1 | 1 | 1 | 10 |
| Chey (2010) | 1 | 1 | 1 | 0 | 1 | 1 | 1 | 1 | 1 | 1 | 9 |
| El-Salhy (2011) | 1 | 1 | 1 | 1 | 1 | 1 | 1 | 1 | 1 | 1 | 10 |
| Ozdil (2011) | 1 | 1 | 1 | 1 | 1 | 1 | 1 | 1 | 1 | 1 | 10 |
| Rahman (2012) | 1 | 1 | 1 | 1 | 1 | 1 | 1 | 0 | 1 | 1 | 9 |
| Stoicescu (2012) | 1 | 1 | 1 | 1 | 1 | 1 | 1 | 1 | 1 | 1 | 10 |
| Hilmi (2013) | 1 | 1 | 0 | 1 | 1 | 1 | 1 | 0 | 1 | 1 | 8 |
| Patel (2015) | 1 | 1 | 1 | 1 | 1 | 1 | 1 | 1 | 1 | 1 | 10 |
| Simsek (2015) | 1 | 1 | 1 | 1 | 1 | 1 | 1 | 0 | 1 | 1 | 9 |
| Ucmak (2015) | 1 | 1 | 1 | 1 | 1 | 1 | 1 | 0 | 1 | 1 | 9 |
| Hilpusch (2017) | 1 | 1 | 1 | 1 | 1 | 1 | 1 | 0 | 1 | 1 | 9 |
| Johnsen (2018) | 1 | 1 | 0 | 1 | 0 | 0 | 1 | 0 | 1 | 1 | 6 |
| Paudel (2018) | 1 | 1 | 0 | 0 | 0 | 0 | 0 | 0 | 0 | 1 | 3 |

| Author (Year) | Did the study address a clearly focused issue? | Was the cohort recruited in an acceptable way? | Was the exposure accurately measured to minimise bias? | Was the outcome accurately measured to minimise bias? | Have the authors identified all important confounding factors? | Have they taken account of the confounding factors in the design and/or analysis? | Do you believe the results? | Can the results be applied to the local population? | Do the results of this study fit with other available evidence? | What are the implications of this study for practice? | Total score |
| --- | --- | --- | --- | --- | --- | --- | --- | --- | --- | --- | --- |
| El-Salhy (2019) | 1 | 1 | 1 | 1 | 1 | 1 | 1 | 1 | 1 | 1 | 10 |
| Asghar (2020) | 1 | 1 | 0 | 0 | 0 | 0 | 1 | 1 | 1 | 1 | 6 |

**Supplementary table 4. Appraisal of studies evaluating prevalence of PEI**

| Author (Year) | Did the study address a clearly focused issue? | Was the cohort recruited in an acceptable way? | Was the exposure accurately measured to minimise bias? | Was the outcome accurately measured to minimise bias? | Have the authors identified all important confounding factors? | Have they taken account of the confounding factors in the design and/or analysis? | Do you believe the results? | Can the results be applied to the local population? | Do the results of this study fit with other available evidence? | What are the implications of this study for practice? | Total score |
| --- | --- | --- | --- | --- | --- | --- | --- | --- | --- | --- | --- |
|  |  |  |  |  |  |  |  |  |  |  |  |
|  |  |  |  |  |  |  |  |  |  |  |  |
| Leeds (2010) | 1 | 1 | 1 | 1 | 1 | 1 | 1 | 1 | 1 | 1 | 10 |
| Talley (2017) | 1 | 1 | 1 | 1 | 1 | 1 | 1 | 1 | 1 | 1 | 10 |

**Supplementary table 5. Appraisal of studies evaluating prevalence of SIBO**

| Author (Year) | Did the study address a clearly focused issue? | Was the cohort recruited in an acceptable way? | Was the exposure accurately measured to minimise bias? | Was the outcome accurately measured to minimise bias? | Have the authors identified all important confounding factors? | Have they taken account of the confounding factors in the design and/or analysis? | Do you believe the results? | Can the results be applied to the local population? | Do the results of this study fit with other available evidence? | What are the implications of this study for practice? | Total score |
| --- | --- | --- | --- | --- | --- | --- | --- | --- | --- | --- | --- |
|  |  |  |  |  |  |  |  |  |  |  |  |
|  |  |  |  |  |  |  |  |  |  |  |  |
| Pimentel (2000) | 1 | 1 | 1 | 1 | 0 | 0 | 1 | 1 | 1 | 1 | 8 |
| Ghoshal (2003) | 1 | 1 | 1 | 1 | 1 | 1 | 0 | 0 | 0 | 1 | 7 |
| Pimentel (2003) | 1 | 1 | 1 | 1 | 1 | 1 | 1 | 1 | 1 | 1 | 10 |
| Pimentel (2003) | 1 | 1 | 1 | 1 | 0 | 0 | 1 | 0 | 1 | 1 | 7 |
| Pimentel (2004) | 1 | 1 | 1 | 1 | 1 | 1 | 1 | 1 | 1 | 1 | 10 |
| Lupascu (2005) | 1 | 1 | 1 | 1 | 1 | 1 | 1 | 0 | 1 | 1 | 9 |
| Nucera (2005) | 1 | 1 | 1 | 1 | 1 | 1 | 1 | 0 | 1 | 1 | 9 |
| Sharara (2006) | 1 | 1 | 1 | 1 | 1 | 1 | 0 | 0 | 1 | 1 | 8 |
| Esposito (2007) | 1 | 1 | 1 | 1 | 0 | 1 | 1 | 0 | 1 | 1 | 8 |
| Gupta (2007) | 1 | 1 | 0 | 0 | 1 | 1 | 1 | 0 | 1 | 1 | 7 |
| Majewski (2007) | 1 | 1 | 1 | 1 | 1 | 1 | 1 | 1 | 1 | 1 | 10 |
| Posserud (2007) | 1 | 1 | 1 | 1 | 1 | 1 | 1 | 1 | 1 | 1 | 10 |
| Carrara (2008) | 1 | 1 | 1 | 1 | 0 | 1 | 1 | 1 | 1 | 1 | 9 |
| Author (Year) | Did the study address a clearly focused issue? | Was the cohort recruited in an acceptable way? | Was the exposure accurately measured to minimise bias? | Was the outcome accurately measured to minimise bias? | Have the authors identified all important confounding factors? | Have they taken account of the confounding factors in the design and/or analysis? | Do you believe the results? | Can the results be applied to the local population? | Do the results of this study fit with other available evidence? | What are the implications of this study for practice? | Total score |
| Grover (2008) | 1 | 1 | 1 | 0 | 1 | 1 | 1 | 1 | 1 | 1 | 9 |
| Rana (2008) | 1 | 1 | 1 | 1 | 0 | 0 | 1 | 1 | 1 | 1 | 8 |
| Jung (2009) | 1 | 1 | 1 | 1 | 0 | 0 | 1 | 0 | 1 | 1 | 7 |
| Ghoshal (2009) | 1 | 1 | 1 | 1 | 1 | 1 | 1 | 1 | 1 | 1 | 10 |
| Koza (2009) | 1 | 1 | 1 | 0 | 0 | 0 | 0 | 0 | 0 | 0 | 3 |
| Mann (2009) | 1 | 1 | 1 | 0 | 0 | 0 | 0 | 0 | 0 | 0 | 3 |
| Peralta (2009) | 1 | 1 | 1 | 0 | 0 | 0 | 1 | 0 | 1 | 1 | 6 |
| Rana (2009) | 1 | 1 | 1 | 0 | 1 | 1 | 1 | 0 | 0 | 1 | 7 |
| Hwang (2010) | 1 | 1 | 1 | 0 | 0 | 1 | 0 | 0 | 0 | 1 | 5 |
| Law (2010) | 1 | 1 | 1 | 1 | 1 | 0 | 1 | 1 | 1 | 1 | 9 |
| Lombardo (2010) | 1 | 1 | 1 | 0 | 1 | 1 | 1 | 1 | 1 | 1 | 9 |
| Reddymasu (2010) | 1 | 1 | 1 | 1 | 1 | 1 | 1 | 0 | 1 | 1 | 9 |
| Kim (2011) | 1 | 1 | 1 | 1 | 1 | 1 | 1 | 0 | 1 | 1 | 9 |
| Sachdeva (2011) | 1 | 1 | 1 | 1 | 1 | 1 | 1 | 0 | 1 | 1 | 9 |
| Yakoob (2011) | 1 | 1 | 1 | 0 | 0 | 0 | 0 | 0 | 0 | 1 | 4 |
| Youn (2011) | 1 | 1 | 1 | 1 | 1 | 1 | 1 | 0 | 1 | 1 | 9 |
| Meyrat (2012) | 1 | 1 | 1 | 1 | 0 | 1 | 1 | 1 | 1 | 1 | 9 |

| Author (Year) | Did the study address a clearly focused issue? | Was the cohort recruited in an acceptable way? | Was the exposure accurately measured to minimise bias? | Was the outcome accurately measured to minimise bias? | Have the authors identified all important confounding factors? | Have they taken account of the confounding factors in the design and/or analysis? | Do you believe the results? | Can the results be applied to the local population? | Do the results of this study fit with other available evidence? | What are the implications of this study for practice? | Total score |
| --- | --- | --- | --- | --- | --- | --- | --- | --- | --- | --- | --- |
| Pyleris (2012) | 1 | 1 | 1 | 0 | 1 | 1 | 1 | 1 | 1 | 1 | 9 |
| Rana (2012) | 1 | 1 | 1 | 1 | 1 | 1 | 1 | 1 | 1 | 1 | 10 |
| Stoicescu (2012) | 1 | 1 | 1 | 0 | 0 | 0 | 0 | 0 | 1 | 1 | 5 |
| De Roest (2013) | 1 | 1 | 1 | 1 | 0 | 0 | 1 | 1 | 1 | 1 | 8 |
| Lee (2013) | 1 | 1 | 1 | 1 | 1 | 1 | 1 | 1 | 1 | 1 | 10 |
| Moraru (2013) | 1 | 1 | 1 | 1 | 0 | 0 | 1 | 0 | 1 | 1 | 7 |
| Dupont (2014) | 1 | 1 | 1 | 1 | 1 | 1 | 1 | 0 | 1 | 1 | 9 |
| Ghoshal (2014) | 1 | 1 | 1 | 1 | 1 | 1 | 1 | 0 | 1 | 1 | 9 |
| Melchior (2014) | 1 | 1 | 1 | 1 | 1 | 1 | 1 | 0 | 1 | 1 | 9 |
| Srivastava (2014) | 1 | 1 | 1 | 1 | 0 | 1 | 1 | 0 | 1 | 1 | 8 |
| Zhao (2014) | 1 | 1 | 1 | 1 | 0 | 1 | 1 | 0 | 1 | 1 | 8 |
| Abbasi (2015) | 1 | 1 | 1 | 0 | 1 | 1 | 1 | 0 | 1 | 1 | 8 |
| Lasa (2015) | 1 | 1 | 1 | 0 | 0 | 0 | 0 | 0 | 0 | 1 | 4 |
| Soldi (2015) | 1 | 1 | 1 | 0 | 0 | 0 | 0 | 0 | 0 | 1 | 4 |
| Chu (2016) | 1 | 1 | 1 | 1 | 0 | 1 | 1 | 0 | 1 | 1 | 8 |
| Nagasako (2016) | 1 | 1 | 1 | 1 | 1 | 1 | 1 | 0 | 1 | 1 | 9 |

| Author (Year) | Did the study address a clearly focused issue? | Was the cohort recruited in an acceptable way? | Was the exposure accurately measured to minimise bias? | Was the outcome accurately measured to minimise bias? | Have the authors identified all important confounding factors? | Have they taken account of the confounding factors in the design and/or analysis? | Do you believe the results? | Can the results be applied to the local population? | Do the results of this study fit with other available evidence? | What are the implications of this study for practice? | Total score |
| --- | --- | --- | --- | --- | --- | --- | --- | --- | --- | --- | --- |
| Shimura (2016) | 1 | 1 | 1 | 1 | 1 | 1 | 1 | 0 | 0 | 1 | 8 |
| Ding (2017) | 1 | 1 | 1 | 0 | 0 | 0 | 1 | 0 | 1 | 1 | 6 |
| Jung (2017) | 1 | 1 | 1 | 1 | 0 | 1 | 1 | 1 | 1 | 1 | 9 |
| Moraru (2017) | 1 | 1 | 1 | 1 | 0 | 0 | 1 | 0 | 0 | 1 | 6 |
| Jung (2018) | 1 | 1 | 1 | 1 | 0 | 1 | 1 | 1 | 1 | 1 | 9 |
| Lee (2018) | 1 | 1 | 1 | 1 | 0 | 1 | 0 | 0 | 1 | 1 | 7 |
| Huang (2019) | 1 | 1 | 1 | 1 | 1 | 1 | 0 | 0 | 1 | 1 | 8 |
| Lee (2019) | 1 | 1 | 1 | 1 | 1 | 1 | 1 | 1 | 1 | 1 | 10 |
| Leventogiannis (2019) | 1 | 1 | 1 | 1 | 1 | 1 | 1 | 1 | 1 | 1 | 10 |
| Rezaie (2019) | 1 | 1 | 1 | 1 | 1 | 1 | 1 | 0 | 1 | 1 | 9 |
| Wu (2019) | 1 | 1 | 1 | 1 | 1 | 1 | 1 | 0 | 1 | 1 | 9 |
| Li (2020) | 1 | 1 | 0 | 0 | 1 | 1 | 1 | 0 | 1 | 1 | 7 |
| Sundin (2020) | 1 | 1 | 0 | 0 | 1 | 1 | 0 | 0 | 0 | 1 | 5 |
| Yang (2020) | 1 | 1 | 0 | 0 | 1 | 1 | 0 | 0 | 0 | 1 | 5 |
| Zhuang (2020) | 1 | 1 | 1 | 1 | 1 | 1 | 1 | 0 | 1 | 1 | 9 |

**Supplementary table 6. Summary of the studies that reported the prevalence of BAD based upon a positive SeHCAT study in patients fulfilling IBS criteria, ordered by year of publication.**

| Author | Year | Country | Study design | Total number of IBS patients | Number of patients with a SeHCAT retention rate at 7 days | | | | | Crude pooled rate(s) | Diagnostic criteria for IBS |
| --- | --- | --- | --- | --- | --- | --- | --- | --- | --- | --- | --- |
|  |  |  |  |  | <5% | <10% | <11% | <11·7% | <15% |  |  |
| Galatola et al.[32] | 1992 | Italy | Cross-sectional study | 98 | ·· | ·· | ·· | 56 | ·· | 57·1% | Rome I |
| Sinha et al.[33] | 1998 | UK | Retrospective cross-sectional study | 17 | ·· | ·· | ·· | ·· | 9 | 52·9% | Manning |
| Smith et al.[34] | 2000 | UK | Cross-sectional study | 197 | ·· | 65 | ·· | ·· | ·· | 33·0% | Rome I |
| Fernandez-Banares et al.[35] | 2007 | Spain | Cross-sectional study | 32 | ·· | ·· | 14 | ·· | ·· | 43·8% | Rome II |
| Gracie et al.[36] | 2012 | UK | Retrospective cross-sectional study | 77 | 2 | 6 | ·· | ·· | 21 | 2·6%, 7·8%, 27·3% | Rome III |
| Bajor et al.[37] | 2014 | Sweden | Prospective case-control study | 141 | 14 | 26 | ·· | ·· | 45 | 9·9%, 18·4%, 31·9% | Rome II |
| Aziz et al.[38] | 2015 | UK | Cross-sectional study | 118 | 12 | 20 | ·· | ·· | 28 | 10·2%, 16·9%, 23·7% | Rome III |
| Fernandez-Banares et al.[39] | 2015 | Spain | Randomised control trial | 26 | 14 | 17 | ·· | ·· | 22 | 53·8%, 65·3%, 84·6% | Rome III |
| Jeffery et al.[40] | 2020 | Ireland | Prospective case-control study | 45 | 4 | 11 | ·· | ·· | 18 | 8·9%, 24·4%, 40·0% | Rome IV |
| Shiha et al.[41] | 2020 | UK | Cross-sectional study | 173  139 | 16  15 | 32  27 | ··  ·· | ··  ·· | 63  53 | 9·2%, 18·4%, 36·4%  10·8%, 19·4%, 38·1% | Rome III  Rome IV |

**Supplementary table 7. Summary of the studies that reported the prevalence of BAD based upon an elevated level of 48-hour faecal bile acids in patients fulfilling IBS criteria, ordered by year of publication.**

| Author | Year | Country | Study design | Definition for an elevated level of total faecal bile acids over 48 hours | Total number of patients | Number of patients with an elevated level of total faecal bile acids | Crude pooled rate | Diagnostic criteria used for IBS |
| --- | --- | --- | --- | --- | --- | --- | --- | --- |
| Camilleri et al.[42] | 2014 | US | Cross-sectional study | >2,337 μmol | 54 | 19 | 35·2% | Rome III |
| Donato et al.[43] | 2018 | US | Cross-sectional study | >2,619 μmol | 55 | 17 | 30·9% | Rome III |
| Vijayvargiya et al.[44] | 2018 | US | Prospective case-control study | >2,337 μmol | 938 | 69 | 7·4% | Rome III |
| Vijayvargiya et al.[45] | 2020 | US | Randomised control study | >2,337 μmol | 30 | 9 | 30·0% | Rome III |

**Supplementary table 8. Summary of the studies that reported the prevalence of BAD based upon an elevated level of 7α-C4 in patients fulfilling IBS criteria, ordered by year of publication.**

| Author | Year | Country | Study design | Definition for an elevated 7α-C4 level | Total number of patients | Number of patients with an elevated 7α-C4 level | Crude pooled rate | Diagnostic criteria used for IBS |
| --- | --- | --- | --- | --- | --- | --- | --- | --- |
| Bajor et al.[37] | 2014 | Sweden | Prospective case-control study | >8·0 mg/mol for men  >6·0 mg/mol for women | 133 | 30 | 22·6% | Rome II |
| Camilleri et al.[42] | 2014 | US | Cross-sectional study | >47·1 ng/mL | 54 | 13 | 24·1% | Rome III |
| Dior et al.[46] | 2016 | France | Prospective case-control study | >13·63 ng/mL | 15 | 5 | 33·3% | Rome III |
| Vijayvargiya et al.[45] | 2020 | US | Randomised control study | >52·5 ng/mL | 30 | 4 | 13·3% | Rome III |

**Supplementary table 9. Summary of the studies that reported the prevalence of lactose malabsorption +/- intolerance based upon a positive hydrogen breath test in patients fulfilling IBS criteria, ordered by year of publication.**

| Author | Year | Country | Study design | Mode of diagnosis of lactose malabsorption/intolerance | Dose of lactose for HBT | Test duration of HBT | Sample intervals of HBT | Cut-off criteria for diagnosis of lactose malabsorption | Total number of IBS patients | Number of IBS patients with lactose malabsorption | Crude pooled rate for lactose malabsorption | Number of IBS patients with lactose intolerance | Crude pooled rate for lactose intolerance | Diagnostic criteria used for IBS |
| --- | --- | --- | --- | --- | --- | --- | --- | --- | --- | --- | --- | --- | --- | --- |
| Vernia et al.[47] | 1995 | Italy | Cross-sectional study | HBT | 0·5 g/kg body weight up to a maximum of 25g | 4 h | 30 min | An increase of H_2_ > 20 ppm above the basal value in ≥ 2 samples | 230 | 157 | 68·2% | ·· | ·· | Manning |
| Böhmer et al.[48] | 1996 | Holland | Prospective case-control study | HBT + glucose blood test | 50 g in 100 mL of water | 1.5 h | at 15, 30, 60, 120, 150 and 180 min | An increase of H_2_ ≥ 20 ppm above the basal value, and a positive glucose blood test, defined as a flat curve with a maximum rise in glucose of 1·1 mmol/L | 70 | 17 | 24·3% | ·· | ·· | Manning |
| Hamm et al.[49] | 1999 | Multi-national (Canada, Germany, Netherlands, UK, US) | Cross-sectional study | HBT | 25 g | 3 h | Not mentioned | An increase of H_2_ > 20 ppm, or CH_4_ > 12 ppm, or  a combined increase of > 15 ppm for both H_2_ and CH_4_ above the basal value | 1122 | 256 | 22·8% | ·· | ·· | Rome I |
| Goldstein et al.[50] | 2000 | Israel | Prospective case-control study | HBT | 18 g | 4 h | 30 min | An increase of H_2_ ≥ 20 ppm, or CH_4_ ≥ 5 ppm | 94 | 77 | 81·9% | ·· | ·· | Rome I |
| Parker et al.[51] | 2001 | UK | Prospective case-control study | HBT | 50 g | 1.5 h | at 30, 60, 90, 120, 150 min | An increase of H_2_ > 20 ppm above the basal value | 122 | 33 | 27·0% | ·· | ·· | Rome I |
| Vernia et al.[52] | 2001 | Italy | Prospective case-control study | HBT | 0·5 g/kg body weight up to a maximum of 25g | 4 h | 30 min | An increase of H_2_ > 20 ppm above the basal value in ≥ 2 samples | 503 | 337 | 67·0% | ·· | ·· | Rome I |
| Pimentel et al.[53] | 2003 | US | Cross-sectional study | HBT | 50 g in 250 mL of water | 3 h | 15 min | A rise in H_2_ or CH_4_ > 20 ppm within 3 h | 19 | 10 | 52·6% | 8 | 42·1% | Rome II |
| Author | Year | Country | Study design | Mode of diagnosis of lactose malabsorption/intolerance | Dose of lactose for HBT | Test duration of HBT | Sample intervals of HBT | Cut-off criteria for diagnosis of lactose malabsorption | Total number of IBS patients | Number of IBS patients with lactose malabsorption | Crude pooled rate for lactose malabsorption | Number of IBS patients with lactose intolerance | Crude pooled rate for lactose intolerance | Diagnostic criteria used for IBS |
| Farup et al.[54] | 2004 | Norway | Prospective case-control study | HBT | 25 g | 1.5 h | at 30, 60, 120 and 180 min | An increase of H_2_ >20 ppm above the lowest preceding value, or peak CH_4_ excretion >12 ppm above baseline, or an increase of combined H_2_ and CH_4_ >15 ppm | 74 | 3 | 4·1% | ·· | ·· | Rome II |
| Vernia et al.[55] | 2004 | Italy | Prospective case-control study | HBT | 0·5 g/kg body weight up to a maximum of 25g | 4 h | 30 min | An increase of H_2_ > 20 ppm above the basal value in ≥ 2 samples | 402 | 290 | 72·1% | ·· | ·· | Rome I |
| Nucera et al.[56] | 2005 | Italy | Cross-sectional study | HBT | 20 g | 4 h | 30 min | An increase of H_2_ > 20 ppm above the basal value | 34 | 22 | 64·7% | ·· | ·· | Rome II |
| Bernardes-Silva et al.[57] | 2007 | Brazil | Prospective case-control study | HBT | 25 g in 250 mL of water | 1.5 h | at 60, 90, 120, 150 and 180 min | An increase of H_2_ ≥ 20 ppm above the basal value | 75 | 31 | 41·3% | 28 | 37·3% | Rome II |
| Gupta et al.[25] | 2007 | India | Prospective case-control study | HBT | 50 g of lactose in 200 mL of water | 4 h | 15 min | A persistent rise in H_2_ of ≥ 12 ppm and ≥ 20 pm above basal level in ≥ 2 samples | 124 | ·· | ·· | 89 | 71·7% | Rome II |
| Corlew-Roath et al.[4] | 2009 | US | Cross-sectional study | HBT | 50 g | Not mentioned | Not mentioned | Not mentioned | 66 | 21 | 31·8% | ·· | ·· | Rome III |
| Ghoshal et al.[58] | 2009 | India | Cross-sectional study | HBT | 50 g in 200 mL of water | 3 - 4 h | 15 min | A persistent rise in H_2_ > 20 ppm above the basal value on ≥ 2 consecutive readings | 192 | 125 | 65·1% | ·· | ·· | Rome II |
| Yakoob et al.[59] | 2011 | Pakistan | Retrospective Case-control study | HBT | 50 g | 2 h | at 30, 60, 90, and 120 min | A rise of H2 > 20 ppm at 90 or 120-minute samples | 119 | ·· | ·· | 25 | 21·0% | Rome III |

| Author | Year | Country | Study design | Mode of diagnosis of lactose malabsorption/intolerance | Dose of lactose for HBT | Test duration of HBT | Sample intervals of HBT | Cut-off criteria for diagnosis of lactose malabsorption | Total number of IBS patients | Number of IBS patients with lactose malabsorption | Crude pooled rate for lactose malabsorption | Number of IBS patients with lactose intolerance | Crude pooled rate for lactose intolerance | Diagnostic criteria used for IBS |
| --- | --- | --- | --- | --- | --- | --- | --- | --- | --- | --- | --- | --- | --- | --- |
| De Roest et al.[60] | 2013 | New Zealand | Cross-sectional study | HBT | 50 g | 3 h or when HBT became positive | 15 min | A rise in H_2_ > 10 ppm above the basal value in two consecutive readings within 60 min of lactose ingestion | 192 | 61 | 31·8% | ·· | ·· | Rome III |
| Ghoshal et al.[2] | 2013 | India | Cross-sectional study | HBT | 12 g, 25 g and 50 g | 3 - 4 h | 15 min | A persistent rise in H_2_ > 20 ppm above the basal value on ≥ 2 consecutive readings | 150 - 50 had 12 g - 50 had 25 g - 50 had 50 g | 12 g: 14  25 g: 41  50 g: 37 | 12 g: 28·0%  25 g: 82·0%  50 g: 74·0% | ·· | ·· | Rome III |
| Yang et al.[3] | 2013 | China | Prospective case-control study | HBT | 10 g, 20 g, and 40 g in 250 mL of water | 3 h | 15 min | An increase of H_2_ > 20 ppm above the basal value on ≥ 2 consecutive readings | 60 | 10 g: 25  20 g: 52  40 g: 56 | 10 g: 41·6%  20 g: 86·7%  40 g: 93·3% | 10 g: 11  20 g: 28  40 g: 51 | 10 g: 18·3%  20 g: 46·7%  40 g: 85·0% | Rome III |
| Zhu et al.[61] | 2013 | China | Prospective case-control study | HBT | 20 g | 3 h | 15 min | An increase of H_2_ > 20 ppm above the basal value on ≥ 2 consecutive readings | 277 | 211 | 76·2% | 149 | 53·8% | Rome III |
| Dainese et al.[62] | 2014 | Spain | Cross-sectional study | HBT | 50 g | 3 h | 30 min | An increase of H_2_ > 25 ppm above the basal value | 51 | 24 | 47·1% | 14 | 27·5% | Rome III |
| Goebel-Stengel et al.[63] | 2014 | Germany | Retrospective case-control study | HBT | 50 g in 200 mL of water | 1.5 h | 10 min | An increase of H_2_ > 20 ppm above the basal value | 2390 | 1023 | 42·8% | 848 | 35·5% | Rome II |
| Jafari et al.[5] | 2014 | Iran | Randomised controlled trial | HBT | Not mentioned | Not mentioned | Not mentioned | Not mentioned | 332 | 214 | 64·5% | ·· | ·· | Rome III |
| Yang et al.[64] | 2014 | China | Prospective case-control study | HBT | 20 g | 3 h | 15 min | An increase of H_2_ ≥ 20 ppm on ≥ 2 consecutive readings | 55 | 48 | 87·3% | 25 | 45·5% | Rome III |
| Wang et al.[65] | Year | Country | Study design | Mode of diagnosis of lactose malabsorption/intolerance | Dose of lactose for HBT | Test duration of HBT | Sample intervals of HBT | Cut-off criteria for diagnosis of lactose malabsorption | Total number of IBS patients | Number of IBS patients with lactose malabsorption | Crude pooled rate for lactose malabsorption | Number of IBS patients with lactose intolerance | Crude pooled rate for lactose intolerance | Diagnostic criteria used for IBS |
| Author | 2015 | China | Cross-sectional study | HBT | 25 g in 100 mL of water | 3 h | 15 min | An increase of H_2_ > 20 ppm above the basal value | 37 | 31 | 83·8% | 15 | 40·5% | Rome III |
| Dabak et al.[66] | 2017 | Turkey | Prospective case-control study | HBT | 25 g in 250 mL of water | 2 h | at 15, 30, 60, 90 and 120 min | An increase of H2 ≥ 20 ppm on ≥ 2 samples within 2 h | 100 | 47 | 47·0% | ·· | ·· | Rome III |
| Xiong et al.[67] | 2017 | China | Prospective case-control study | HBT | 25 g in 100 mL of water | 3 h | 15 min | An increase of H2 > 20 ppm above the basal value on ≥ 2 samples within 3 h | 96 | 82 | 85·4% | 43 | 44·8% | Rome III |
| Almazar et al.[6] | 2018 | US | Prospective case-control study | HBT | 0·5 g/kg of body weight | 2 h | 15 - 30 min | A rise of H_2_ ≥ 20 ppm, or a rise of ≥ 12 ppm for H_2_ and CH_4_ combined | 20 | 3 | 15·0% | ·· | ·· | Rome II |
| Mack et al.[7] | 2020 | Germany | Prospective case-control study | HBT | 50 g | 2.5 h | 30 min | An increase of H2 ≥ 20 ppm above basal value | 37 | 8 | 21·6% | ·· | ·· | Rome IV |

CH4: methane; H_2_: hydrogen; HBT: hydrogen breath test; ppm: parts per million

**Supplementary table 10. Summary of the studies that reported the prevalence of lactose intolerance based upon genotyping studies in patients fulfilling IBS criteria, ordered by year of publication.**

| Author | Year | Country | Study design | Total number of IBS patients | Number of IBS patients with C/C-13910 | Crude pooled rate for C/C-13910 | Number of IBS patients with G/G-22018 | Crude pooled rate for G/G-22018 | Diagnostic criteria used for IBS |
| --- | --- | --- | --- | --- | --- | --- | --- | --- | --- |
| Bernardes-Silva et al.[57] | 2007 | Brazil | Prospective case-control study | 72 underwent genotyping for C/C-13910 75 underwent genotyping for G/G-22018 | 34 | 47·2% | 37 | 49·3% | Rome II |
| Kumar et al.[68] | 2012 | India | Prospective case-control study | 150 | 102 | 68·0% | 97 | 64·7% | Rome III |
| Ghoshal et al.[2] | 2013 | India | Cross-sectional study | 150 | 91 | 60·7% | ·· | ·· | Rome III |
| Yang et al.[69] | 2013 | China | Prospective case-control study | 60 | 60 | 100·0% | ·· | ·· | Rome III |
| Almazar et al.[6] | 2018 | US | Prospective case-control study | 538 | 81 | 15·1% | ·· | ·· | Rome II |

**Supplementary table 11. Summary of the studies that reported the prevalence of fructose malabsorption +/- intolerance based upon a positive hydrogen breath test in patients fulfilling IBS criteria, ordered by year of publication.**

| Author | Year | Country | Study design | Mode of diagnosis of fructose malabsorption/intolerance | Dose of fructose for HBT | Test duration of HBT | Sample intervals of HBT | Cut-off criteria for diagnosis of fructose malabsorption | Total number of IBS patients | Number of IBS patients with fructose malabsorption | Crude pooled rate for fructose malabsorption | Number of IBS patients with fructose intolerance | Crude pooled rate for fructose intolerance | Diagnostic criteria used for IBS |
| --- | --- | --- | --- | --- | --- | --- | --- | --- | --- | --- | --- | --- | --- | --- |
| Goldstein et al.[50] | 2000 | Israel | Prospective case-control study | HBT | 25 g | 4 h | 30 min | A rise of H_2_ ≥ 20 ppm, or CH_4_ ≥ 5 ppm | 94 | 41 | 44·0% | ·· | ·· | Rome I |
| Nucera et al.[56] | 2005 | Italy | Cross-sectional study | HBT | 25 g | 4 h | 30 min | An increase of H_2_ > 20 ppm above the basal value | 34 | 12 | 35·3% | ·· | ·· | Rome II |
| Choi et al.[70] | 2008 | US | Cross-sectional study | HBT | 25 g in 250 mL of water | 5 h | 30 min | An incremental rise in H_2_ and/or CH_4_ of > 5ppm in ≥ 3 samples, or a value > 20 ppm above the baseline value in 2 consecutive samples | 80 | 31 | 38·8% | 28 | 35·0% | Rome II |
| Skoog et al.[71] | 2008 | US | Randomised controlled trial | HBT | 40 g in 330 mL of water | 3 h | 30 min | A rise of H_2_ ≥ 20 ppm over the baseline value | 30 | 21 | 70·0% | 12 | 40·0% | Rome II |
| Corlew-Roath et al.[4] | 2009 | US | Prospective case-control study | HBT | 25 g | Not mentioned | Not mentioned | Not mentioned | 66 | 2 | 3·0% | ·· | ·· | Rome III |
| Berg et al.[9] | 2013 | Norway | Prospective case-control study | HBT | 50 g | 2 -4 h | 30 min | A rise of H_2_ > 20 ppm, or CH_4_ > 12 ppm, or a sum of combined peaks > 15 ppm | 182 | 109 | 59·9% | ·· | ·· | Rome II |
| De Roest et al.[60] | 2013 | New Zealand | Cross-sectional study | HBT | 35 g | 3 h | 15 min | A rise of H_2_ > 10 ppm above the baseline value on 2 consecutive readings within 60 min of fructose ingestion | 192 | 145 | 75·5% | ·· | ·· | Rome III |
| Goebel-Stengel et al.[63] | 2014 | Germany | Retrospective case series | HBT | 50 g in 200 mL water | 1.5 h | 10 min | A rise of H_2_ > 20 ppm above the baseline value | 2390 | 1818 | 76·1% | 1531 | 64·1% | Rome II |

| Author | Year | Country | Study design | Mode of diagnosis of fructose malabsorption/intolerance | Dose of fructose for HBT | Test duration of HBT | Sample intervals of HBT | Cut-off criteria for diagnosis of fructose malabsorption | Total number of IBS patients | Number of IBS patients with fructose malabsorption | Crude pooled rate for fructose malabsorption | Number of IBS patients with fructose intolerance | Crude pooled rate for fructose intolerance | Diagnostic criteria used for IBS |
| --- | --- | --- | --- | --- | --- | --- | --- | --- | --- | --- | --- | --- | --- | --- |
| Melchior et al.[72] | 2014 | France | Cross-sectional study | HBT | 25 g in 250 mL of water | 5 h | 30 min | A rise of H_2_ and/or CH_4_ > 20 ppm | 90 | 20 | 22·2% | 7 | 7·8% | Rome III |
| Sharma et al.[10] | 2014 | India | Prospective case-control study | HBT | 25 g in 250 mL of water | 3 h | 15 min | A sustained rise of H_2_ > 20 ppm above the baseline value | 97 | 14 | 14·4% | ·· | ·· | Rome III |
| Jung et al.[8] | 2018 | Korea | Prospective case-control study | HBT | 15 g and 25 g in 250 mL of water | 3 h | 30 min | A sustained rise of H2 > 20 ppm over the baseline value, or CH_4_ > 10 ppm, or H_2_ and CH_4_ combined > 15 ppm, or a sustained rise in H_2_ and/or CH_4_ of ≥ 5 ppm on ≥ 3 consecutive samples | 35 | 15 g: 7  25 g: 16 | 15 g: 20·0%  25 g: 45·7% | ·· | ·· | Rome III |
| Melchior et al.[73] | 2019 | France | Cross-sectional study | HBT | 25 g in 250 mL of water | 5 h | 30 min | A rise of H_2_ and/or CH_4_ > 20 ppm | 88 | 40 | 45·5% | ·· | ·· | Rome III |
| Mack et al.[7] | 2020 | Germany | Prospective case-control study | HBT | 25 g | 2.5 h | 30 min | An increase of H2 ≥ 20 ppm above basal value | 37 | 9 | 24·3% | ·· | ·· | Rome IV |

CH_4_: methane; H_2_: hydrogen; HBT: hydrogen breath test; ppm: parts per million

**Supplementary table 12. Summary of the studies that reported the prevalence of alternate or mixed forms of carbohydrate malabsorption in patients fulfilling IBS criteria, ordered by year of publication.**

| Author | Year | Country | Study design | Type of malabsorption | Mode of diagnosis of malabsorption | Substrate used for HBT | Dose of substrate for HBT | Test duration | Sample intervals of HBT | Cut-off criteria for diagnosis of malabsorption | Total number of IBS patients | Number of IBS patients with malabsorption | Crude pooled rate for malabsorption | Number of IBS patients with intolerance | Crude pooled rate for intolerance | Diagnostic criteria used for IBS |
| --- | --- | --- | --- | --- | --- | --- | --- | --- | --- | --- | --- | --- | --- | --- | --- | --- |
| Nelis et al.[74] | 1990 | Netherlands | Prospective case-control study | Combined fructose and sorbitol | HBT | Fructose-sorbitol | 25 g of fructose + 5 g of sorbitol in 150 mL of water | 3 h | 30 min | An increase of H_2_ > 20 ppm above the basal value | 70 | 22 | 31·4% | 12 | 17·1% | Kruis |
| Goldstein et al.[50] | 2000 | Israel | Prospective case-control study | Combined fructose and sorbitol | HBT | Fructose-sorbitol | 25 g of fructose + 5 g of sorbitol | 4 h | 30 min | An increase of H_2_ ≥ 20 ppm, or CH_4_ ≥ 5 ppm | 94 | 66 | 70·2% | ·· | ·· | Rome I |
| Nucera et al.[56] | 2005 | Italy | Cross-sectional study | Sorbitol | HBT | Sorbitol | 20 g | 4 h | 30 min | An increase of H_2_ > 20 ppm above the basal value | 34 | 12 | 35·3% | ·· | ·· | Rome II |
| Fernandez-Banares et al.[35] | 2007 | Spain | Cross-sectional study | Lactose  Combined fructose and sorbitol | HBT | Lactose  Fructose-sorbitol | 20 g of lactose in 250 mL of water  20 g of fructose + 3.5 g of sorbitol in 250 mL of water | 3 h  3 h | 15 min  15 min | An increase of H_2_ > 20 ppm above the basal value | 32 | 3 (for lactose malabsorption and combined fructose-sorbitol malabsorption, breakdown data not available) | 9·4% | ·· | ·· | Rome II |
| Yao et al.[75] | 2014 | Australia | Prospective case-control study | Sorbitol  Mannitol | HBT | Sorbitol  Mannitol | 10 g  10 g | 4 h  4 h | 15 min  15 min | A rise of H_2_ ≥ 10 ppm above the baseline value on 2 consecutive readings, or CH_4_ ≥ 15 ppm from baseline | 20 | 12 sorbitol malabsorption  4 mannitol malabsorption | 60·0% sorbitol malabsorption  20·0% mannitol malabsorption | 3 sorbitol malabsorption  3 mannitol  malabsorption | 15·0% sorbitol malabsorption  15·0% mannitol malabsorption | Rome III |
| Mack et al.[7] | 2020 | Germany | Prospective case-control study | Sorbitol | HBT | Sorbitol | 10 g | 2.5 h | 30 min | An increase of H2 ≥ 20 ppm above basal value | 37 | 19 | 51·4% | ·· | ·· | Rome IV |

CH_4_: methane; H_2_: hydrogen; HBT: hydrogen breath test; ppm: parts per million

**Supplementary table 13. Summary of the studies that reported the prevalence of MC in patients fulfilling IBS criteria, ordered by year of publication.**

| Author | Year | Country | Study design | Subtype(s) of MC evaluated | Definition for | | | Total number of IBS patients | Number of IBS patients with | | | Crude pooled rate(s) | | | Diagnostic criteria used for IBS |
| --- | --- | --- | --- | --- | --- | --- | --- | --- | --- | --- | --- | --- | --- | --- | --- |
|  |  |  |  |  | MC | LC | CC |  | MC | LC | CC | MC | LC | CC |  |
| Chadwick et al.[76] | 2002 | New Zealand | Prospective case-control study | LC | ·· | Not specifically mentioned. Stated histologic criteria for classic LC was used. | ·· | 77 | ·· | 8 | ·· | ·· | 10·4% | ·· | Rome I |
| Tuncer et al.[77] | 2003 | Turkey | Cross-sectional study | LC   CC | ·· | Flattening of surface epithelium, degenerative cuboidal changes, significant intraepithelial lymphocyte infiltration (> 20 lymphocytes against 100 epithelial cells), and mononuclear cell infiltration in the lamina propria | Sub-epithelial collagenous band thickness of > 15 μm investigated with trichrome dye and H&E stain | 30 | ·· | 7 | 0 | ·· | 23·3% | 0·0% | Manning |
| Tavakkoli et al.[78] | 2008 | Iran | Cross-sectional study | LC   CC | ·· | The presence of > 20 lymphocytes per 100 epithelial cells without thickened collagen band | Thickening of the sub-epithelial collagenous band > 10 μm with surface epithelial damage, increased plasma cells, eosinophil in lamina propria, and increased intraepithelial lymphocytes | 138 | ·· | 3 | 10 | ·· | 2·2% | 7·2% | Rome II |
| Chey et al.[79] | 2010 | US | Prospective case-control study | LC   CC | ·· | An increase in intraepithelial lymphocytes (> 15 lymphocytes per 100 epithelial cells) and surface epithelial damage with increased lamina propria plasma cells and absent or minimal crypt architectural disruption | An increase or irregularity in sub-epithelial collagen (> 10 μm) that typically trapped superficial capillaries as well as the other inflammatory changes seen in LC | 466 | ·· | 4 | 3 | ·· | 0·9% | 0·6% | Rome II |

| Author | Year | Country | Study design | Subtype(s) of MC evaluated | Definition for | | | Total number of IBS patients | Number of IBS patients with | | | Crude pooled rate(s) | | | Diagnostic criteria used for IBS |
| --- | --- | --- | --- | --- | --- | --- | --- | --- | --- | --- | --- | --- | --- | --- | --- |
|  |  |  |  |  | MC | LC | CC |  | MC | LC | CC | MC | LC | CC |  |
| El-Salhy et al.[80] | 2011 | Norway | Cross-sectional study | LC   CC | ·· | An increase in intraepithelial lymphocytes to > 15 lymphocytes per 100 epithelial cells, surface epithelial damage with increased lamina propria, plasma cells and absent or minimal crypt architectural disruption | An increase or irregularity in sub-epithelial collagen (> 10 μm) that typically trapped superficial capillaries as well as the other inflammatory changes seen in LC | 968 | ·· | 5 | 2 | ·· | 0·5% | 0·2% | Rome III |
| Ozdil et al.[81] | 2011 | Turkey | Prospective case-control study | LC   CC | ·· | An increase in intraepithelial lymphocytes to > 15 lymphocytes per 100 epithelial cells, surface epithelial damage with increased lamina propria, plasma cells and absent or minimal crypt architectural disruption | An increase or irregularity in sub-epithelial collagen (> 10 μm) that typically trapped superficial capillaries as well as the other inflammatory changes seen in LC | 226 | ·· | 7 | 0 | ·· | 3·1% | 0·0% | Rome III |
| Rahman et al.[82] | 2012 | Bangladesh | Cross-sectional study | LC   CC | ·· | Intraepithelial lymphocytes > 20 per 100 epithelial cells and chronic inflammatory cell infiltration in the lamina propria | Intraepithelial lymphocytes ≥ 20 per 100 epithelial cells and chronic inflammatory cell infiltration in the lamina propria with sub-epithelial collagen band thickening ≥ 10 μm | 60 | ·· | 22 | 0 | ·· | 36·7% | 0·0% | Rome II |

| Author | Year | Country | Study design | Subtype(s) of MC evaluated | Definition for | | | Total number of IBS patients | Number of IBS patients with | | | Crude pooled rate(s) | | | Diagnostic criteria used for IBS |
| --- | --- | --- | --- | --- | --- | --- | --- | --- | --- | --- | --- | --- | --- | --- | --- |
|  |  |  |  |  | MC | LC | CC |  | MC | LC | CC | MC | LC | CC |  |
| Stoicescu et al. [26] | 2012 | Hungary | Cross-sectional study | LC   CC | ·· | > 20 intraepithelial lymphocytes per 100 epithelial cells and an increased number of inflammatory cells in the lamina propria, associated with normal collagen band. Superficial epithelial injuries were also identified. | Sub-epithelial collagenous band thickening > 10 μm, increased number of inflammatory cells in lamina propria, increased intraepithelial lymphocytes and possible surface epithelial damage | 132 | ·· | 3 | 1 | ·· | 2·3% | 0·8% | Rome III |
| Hilmi et al.[83] | 2013 | Malaysia | Prospective case-control study | LC   CC | ·· | Increased intraepithelial lymphocytes of ≥ 20 per 100 epithelial cells in conjunction with surface epithelial damage, normal collagen layer and normal crypt architecture. | Abnormally thickened sub-epithelial collagen band of ≥ 10 μm, chronic inflammation including increased intraepithelial lymphocytes and normal crypt architecture | 74 | ·· | 0 | 1 | ·· | 0·0% | 1·4% | Rome III |
| Patel et al.[84] | 2015 | Canada | Cross-sectional study | MC | Not mentioned | ·· | ·· | 559 | 12 | ·· | ·· | 2·1% | ·· | ·· | Rome III |

| Author | Year | Country | Study design | Subtype(s) of MC evaluated | Definition for | | | Total number of IBS patients | Number of IBS patients with | | | Crude pooled rate(s) | | | Diagnostic criteria used for IBS |
| --- | --- | --- | --- | --- | --- | --- | --- | --- | --- | --- | --- | --- | --- | --- | --- |
|  |  |  |  |  | MC | LC | CC |  | MC | LC | CC | MC | LC | CC |  |
| Simsek et al.[85] | 2015 | Turkey | Prospective case-control study | LC   CC | ·· | Flattening of the surface epithelium, degenerative cuboidal changes, significant intraepithelial lymphocyte infiltration (> 20 lymphocytes for every 100 epithelial cells), and mononuclear cell infiltration in the lamina propria | Sub-epithelial collagenous bands >10 μm in thickness that were dyed with trichrome and H&E stains | 91 | ·· | 7 | 2 | ·· | 7·7% | 2·2% | Rome III |
| Ucmak et al.[86] | 2015 | Turkey | Cross-sectional study | MC | ·· | Chronic inflammatory cell infiltration in lamina propria in addition to > 20 lymphocytes in 100 epithelial cells counted during examination | Sub-epithelial collagenous band ≥ 10 μm, determined by Masson-Trichrome staining | 89 | ·· | 19 | 0 | ·· | 21·3% | 0·0% | Rome III |
| Hilpusch et al.[87] | 2017 | Norway | Cross-sectional study | LC   CC | ·· | An increase in the number of lymphocytes in the colon mucosa (intraepithelial lymphocytes > 20 per 100 epithelial cells) | CC will also contain a sub-epithelial collagen band (at least 10 μm, typically 2–7 μm) | 87 | ·· | 4 | 0 | ·· | 4·6% | 0·0% | Rome III |
| Johnsen et al.[88] | 2018 | Norway | Randomised controlled trial | MC | Not mentioned | ·· | ·· | 90 | 4 | ·· | ·· | 4·4% | ·· | ·· | Rome III |
| Paudel et al.[89] | 2018 | Nepal | Cross-sectional study | MC | Not mentioned | ·· | ·· | 140 | 1 | ·· | ·· | 0·7% | ·· | ·· | Rome IV |

| Author | Year | Country | Study design | Subtype(s) of MC evaluated | Definition for | | | Total number of IBS patients | Number of IBS patients with | | | Crude pooled rate(s) | | | Diagnostic criteria used for IBS |
| --- | --- | --- | --- | --- | --- | --- | --- | --- | --- | --- | --- | --- | --- | --- | --- |
|  |  |  |  |  | MC | LC | CC |  | MC | LC | CC | MC | LC | CC |  |
| El-Salhy et al.[90] | 2019 | Norway | Retrospective case series | LC   CC | ·· | In the presence of an increase in intraepithelial lymphocytes (> 15 lymphocytes per 100 epithelial cells), surface epithelial damage, increased lamina propria plasma cells, and absent or minimal crypt architectural distribution | An increase or irregularity in subepithelial collagen (> 10 μm) as well as the other inflammatory changes seen in LC | 1489 | ·· | 7 | 2 | ·· | 0·5% | 0·1% | Rome III |
| Asghar et al.[91] | 2020 | UK | Cross-sectional study | MC | Not mentioned | ·· | ·· | 352 | 12 | ·· | ·· | 3·4% | ·· | ·· | Rome IV |

CC: collagenous colitis; LC: lymphocytic colitis; MC: microscopic colitis

**Supplementary table 14. Summary of the studies that reported the prevalence of PEI in patients fulfilling IBS criteria, ordered by year of publication.**

| Author | Year | Country | Study design | Definition for PEI | Total number of IBS patients | Number of IBS patients with PEI | Crude pooled rate | Diagnostic criteria used for IBS |
| --- | --- | --- | --- | --- | --- | --- | --- | --- |
| Leeds et al.[92] | 2010 | UK | Prospective case-control study | Faecal elastase-1 <100 μg/g | 314 | 19 | 6·1% | Rome II |
| Talley et al.[93] | 2017 | Australia | Cross-sectional study | Faecal elastase-1 <200 μg/g | 164 | 3 | 1·8% | Rome III |

**Supplementary table 15. Summary of the studies that reported the prevalence of SIBO in patients fulfilling IBS criteria, ordered by year of publication.**

| Author | Year | Country | Study design | Mode of diagnosis of SIBO | Substrate used for HBT | Dose of substrate for HBT | Test duration | Sample intervals of HBT | Cut-off criteria for diagnosis of SIBO | Total number of IBS patients | Number of IBS patients with SIBO | Crude pooled rate for SIBO | Diagnostic criteria used for IBS |
| --- | --- | --- | --- | --- | --- | --- | --- | --- | --- | --- | --- | --- | --- |
| Pimentel et al.[94] | 2000 | US | Cross-sectional study | HBT | Lactulose | 10 g | 3 h | 15 min | 2 distinct peaks representing small intestinal (early peak) and colonic (late peak) flora, or   H_2_ production within 90 min after ingestion of lactulose, or   An absolute change in H_2_ concentration of > 20 ppm | 202 | 157 | 77·7% | Rome |
| Ghoshal et al.[24] | 2003 | India | Prospective case-control study | HBT  Small bowel aspirate | Glucose  ·· | 100 g in 200 mL of water  ·· | 3 h  ·· | 10 min  ·· | Rise of breath H_2_ by 14 ppm above basal level following glucose administration  Positive culture of small bowel aspirate | 12 | 0  3 | 0·0%  25·0% | Rome |
| Pimentel et al.[95] | 2003 | US | Randomised controlled trial | HBT | Lactulose | 10 g | 3 h | 15 min | A rise of breath H_2_ or CH_4_ > 20 ppm before 90 min during 180 min, and the presence of the traditional two peaks | 101 | 84 | 83·2% | Rome |
| Pimentel et al.[12] | 2003 | US | Cross-sectional study | HBT | Lactulose | 10 g | 3 h | 15 min | A rise of > 20 ppm in H_2_ or CH_4_ within 90 min of lactulose ingestion | 19 | 14 | 73·7 | Rome II |
| Pimentel et al.[96] | 2004 | US | Prospective case-control study | HBT | Lactulose | 10 g | 3 h | 15 min | A rise of breath H_2_ or CH_4_ > 20 ppm before 90 min during 180 min | 111 | 93 | 83·8% | Rome |
| Lupascu et al.[97] | 2005 | Italy | Prospective case-control study | HBT | Glucose | 50 g | 2 h | 10 min | An increase over the baseline of H_2_ level > 12 ppm | 65 | 20 | 30·8% | Rome II |
| Nucera et al.[56] | 2005 | Italy | Cross-sectional study | HBT | Lactulose | 10 g | 4 h | 15 min | At least 2 distinct peaks, consisting of 2 consecutive H_2_ values > 10 ppm above basal value after lactulose ingestion, or  H_2_ production within 90 min after lactulose ingestion | 98 | 64 | 65·3% | Rome II |
| Sharara et al.[98] | 2006 | US | Randomised controlled trial | HBT | Lactulose | 10 g | 3 h | 15 min | An early rise in H_2_ of > 20 ppm within the first 90 min | 70 | 0 | 0·0% | Rome II |
| Esposito et al.[99] | 2007 | Italy | Cross-sectional study | HBT | Lactulose | 75 g | 3 h | 15 min | An increase of H_2_ > 10 ppm over basal values | 73 | 33 | 45·2% | Rome II |
| Gupta et al.[25] | 2007 | India | Prospective case-control study | HBT | Glucose | 100 g in 200 mL of water | 4 h | 15 min | A persistent rise in breath H_2_ by ≥ 12 ppm and ≥ 20 ppm above basal level on ≥ 2 consecutive readings | 69 | 9 | 13·0% | Rome II |
| Majewski et al.[100] | 2007 | US | Cross-sectional study | HBT | Glucose | 50 g in 150 mL of water | 1.5 h | at 30, 45, 60, 75 and 90 min | A H_2_ and/or CH_4_ peak > 20 ppm when the baseline was < 10 ppm, or  An increase of H_2_ and/or CH_4_ > 12 ppm when baseline was > 10 ppm | 204 | 93 | 45·6% | Rome II |
| Posserud et al.[101] | 2007 | Sweden | Prospective case-control study | Small bowel aspirate | ·· | ·· | ·· | ·· | > 10^5^ cfu/mL of colonic bacteria | 162 | 7 | 4·3% | Rome II |
| Carrara et al.[102] | 2008 | Italy | Cross-sectional study | HBT | Lactulose | 10 g | 3 h | 15 min | An increase of H_2_ > 20 ppm over basal values within the first 90 min since oral administration of lactulose | 127 | 55 | 43·3% | Rome II |
| Grover et al.[103] | 2008 | India | Prospective case-control study | HBT | Glucose | 50 g in 200 mL of water | 2 h | 15 min | An increase in breath H_2_ > 12 ppm over baseline value | 225 | 25 | 11·1% | Rome II |

| Author | Year | Country | Study design | Mode of diagnosis of SIBO | Substrate used for HBT | Dose of substrate for HBT | Test duration | Sample intervals of HBT | Cut-off criteria for diagnosis of SIBO | Total number of IBS patients | Number of IBS patients with SIBO | Crude pooled rate for SIBO | Diagnostic criteria used for IBS |
| --- | --- | --- | --- | --- | --- | --- | --- | --- | --- | --- | --- | --- | --- |
| Jung et al.[13] | 2009 | Korea | Prospective case-control study | HBT | Lactulose | 10 g of in 100 mL of water | 3 h | 15 min | A peak of H_2_ and/or CH_4_ >20 ppm that occurred 15 min before the colonic peak, or   An elevated fasting H_2_ and/or CH_4_ >12-15 ppm | 38 | 7 | 18·4% | Rome II |
| Ghoshal et al.[58] | 2009 | India | Cross-sectional study | HBT | Glucose | 100 g in 200 mL of water | 3 - 4 h | 15 min | Persistent rise in breath H_2_ by 20 ppm above the basal level on ≥ 2 consecutive readings, or  A rise of H_2_ > 12 ppm above the basal level | 115 | 10 | 8·7% | Rome II |
| Koza et al.[14] | 2009 | Poland | Prospective case-control study | HBT | Lactulose | 10 g in 400 mL of water | 30 min | 15 min | A rise in H_2_ ≥ 12 ppm above baseline level | 44 | 7 | 15·9% | Rome III |
| Mann et al.[15] | 2009 | US | Cross-sectional study | HBT | Lactulose | 10 g in 30 mL of water | 70 min | 5-10 min | A rise of H_2_ > 5 ppm over the fasting value at 60 min or sooner | 258 | 89 | 34·5% | Rome III |
| Peralta et al.[16] | 2009 | Italy | Cross-sectional study | HBT | Lactulose | 25 g | 3 h | 30 min | An early increase of H_2_ > 20 ppm over basal values within 90 min of the oral administration of lactulose, followed by a second distinct peak after additional 15 min or more | 97 | 54 | 55·7% | Rome II |
| Rana et al.[17] | 2009 | India | Prospective case-control study | HBT | Lactulose | 10 g | 4 h | 30 min | A H_2_ or CH_4_ peak > 20 ppm over the baseline value in ≥ 2 samples, or  A concentration of CH_4_ > 10 ppm | 345 | 17 | 4·9% | Rome II |
| Hwang et al.[18] | 2010 | US | Cross-sectional study | HBT | Lactulose | 10 g in 240 mL of water | 2 h | 15 min | Any detection of CH_4_ > 5 ppm | 56 | 28 | 50·0% | Rome |
| Law et al.[104] | 2010 | US | Cross-sectional study | HBT | Lactulose | 10 g in 250 mL of water | 2.5 h | 15 min | A rise in H_2_ ≥ 20 ppm above baseline at or before 90 min from the time of ingestion of lactulose | 555 | 302 | 54·4% | Rome |
| Lombardo et al.[105] | 2010 | Italy | Prospective case-control study | HBT | Glucose | 50 g in 250 mL of water | 2 h | 15 min | An increase over the baseline of H_2_ level > 10 ppm | 200 | 49 | 24·5% | Rome III |
| Reddymasu et al.[106] | 2010 | US | Cross-sectional study | HBT | Glucose | 50 g in 150 mL of water | 2 h | 15 min | H_2_ or CH_4_ peak was > 20 ppm when baseline was < 10 ppm, or  H_2_ or CH_4_ peak increased by > 12 ppm when baseline was ≥ 10 ppm | 98 | 35 | 35·7% | Rome II |
| Kim et al.[107] | 2011 | Korea | Cross-sectional study | HBT | Lactulose | 10 g | 2.5 h | 15 min | Baseline H_2_ concentrations of > 20 ppm, or an increase in the H_2_ concentration of > 20 ppm above the baseline within 60 min, and  Baseline CH_4_ concentrations of > 10 ppm, or an increase in the CH_4_ concentration of > 10 ppm above baseline within 60 min | 86 | 27 | 31·4% | Rome III |
| Sachdeva et al.[108] | 2011 | India | Prospective case-control study | HBT | Glucose | 100 g in 200 mL of water | 3 h | 15 min | A persistent rise in breath H_2_ or CH_4_ > 12 ppm above basal on ≥ 2 readings, or  A fasting CH_4_ concentration of > 10 ppm | 59 | 14 | 23·7% | Rome III |
| Yakoob et al.[59] | 2011 | Korea | Prospective case-control study | HBT | Lactulose | 10 g in 200 mL of water | 3 h | 15 min | H_2_ > 20 ppm at baseline, or   An increase of H_2_ > 20 ppm over baseline within 90 min, or  CH_4_ > 10 ppm at baseline, or  An increase of CH_4_ > 10 ppm over baseline within 90 min | 84 | 46 | 54·8% | Rome II |
| Youn et al.[109] | 2012 | Switzer-land | Cross-sectional study | HBT | Lactulose | 10 g in 400 mL of water | 2 h | 20 min for the first hour, then 30 min for the second hour | An increase in H_2_ ≥ 12 ppm above the basal value within 60 min of ingesting lactulose on the condition that this early rise in H_2_ preceded the second prolonged rise in H_2_ by at least 15 min | 150 | 106 | 70·7% | Rome III |
| Meyrat et al.[110] | 2012 | Greece | Cross-sectional study | Small bowel aspirate | ·· | ·· | ·· | ·· | The presence of > 10^3^ cfu/mL of colonic type aerobic bacteria in the duodenal aspirate. | 112 | 42 | 37·5% | Rome II |

| Author | Year | Country | Study design | Mode of diagnosis of SIBO | Substrate used for HBT | Dose of substrate for HBT | Test duration | Sample intervals of HBT | Cut-off criteria for diagnosis of SIBO | Total number of IBS patients | Number of IBS patients with SIBO | Crude pooled rate for SIBO | Diagnostic criteria used for IBS |
| --- | --- | --- | --- | --- | --- | --- | --- | --- | --- | --- | --- | --- | --- |
| Rana et al.[111] | 2012 | India | Prospective case-control study | HBT | Lactulose  Glucose | 10 g  80 g | 2.5 h | 10 min | Lactulose HBT: Sustained increase in breath concentration of H_2_ or CH_4_ or both ≥ 10 ppm over a baseline value within 90 min  Glucose HBT: Sustained increase in breath concentration of H_2_ or CH_4_ or both ≥ 10 ppm over a baseline value within 120 min | 175 | Lactulose HBT: 60  Glucose HBT: 11 | Lactulose HBT: 34·2%  Glucose HBT: 6·2% | Rome II |
| Stoicescu et al.[26] | 2012 | Romania | Cross-sectional study | HBT | Glucose | 50 g of in 250 mL of water | 2 h | 15 min | Expired H_2_ > 10-12 ppm over baseline | 132 | 58 | 43·9% | Rome III |
| De Roest et al.[60] | 2013 | New Zealand | Cross-sectional study | HBT | Lactulose | 6·7 g | 3 h | 15 min | A rise in breath H_2_ > 10 ppm on 2 consecutive readings above baseline within 60 min | 192 | 23 | 12·0% | Rome III |
| Lee et al.[112] | 2013 | Korea | Prospective case-control study | HBT | Lactulose | 10 g in 200 mL of water | 2.5 h | 15 min | A rise in H_2_ > 20 ppm or CH_4_ > 10 ppm within 90 min | 68 | 42 | 61.8% | Rome III |
| Moraru et al.[27] | 2013 | Romania | Cross-sectional study | HBT | Glucose | 50 g in 250 mL of water | 2 h | 15 min | A breath H_2_ > 20 ppm before 120 min | 72 | 8 | 11·1% | Rome III |
| Dupont et al.[113] | 2014 | US | Cross-sectional study | HBT | Lactulose | 10 g | 2.5 h | 15 min | An increase of ≥ 20 ppm in H_2_ or CH_4_ during the first 90 min of the test | 48 | 20 | 41·7% | Rome II |
| Ghoshal et al.[114] | 2014 | India | Cross-sectional study | HBT          Small bowel aspirate | Lactulose  Glucose       ·· | 10 g of lactulose  100 g of glucose in 200 mL of water   ·· | 4 h for lactulose 3 h for glucose       ·· | 15 min  ·· | A sustained increase in breath H_2_ in ≥ 2 consecutive readings by 12 ppm above basal level following the administration of glucose, or   A characteristic double peak or an early peak (increase in breath H_2_ 20 ppm above basal levels within 90 min) in breath H_2_, or  A fasting breath CH_4_ level of ≥ 10 ppm or increase by ≥ 10 ppm above basal after substrate ingestion   > 10^5^ cfu/mL of bacteria | 80 | Lactulose HBT: 28  Glucose HBT: 4   Small bowel aspirate: 15 | Lactulose HBT: 35·0%  Glucose HBT: 5·0%   Small bowel aspirate: 18·8% | Rome III |
| Melchior et al.[72] | 2014 | France | Cross-sectional study | HBT | Glucose | 75 g in 250 mL of water | 2 h | 15 min | Peak of H_2_ or CH_4_ > 20 ppm, or  Increase of H_2_ or CH_4_ levels > 10 ppm in 2 samples by comparison with individual baseline levels, or  Baseline H_2_ or CH_4_ levels > 20 ppm despite good compliance with the diet | 90 | 1 | 1·1% | Rome III |
| Srivastava et al.[115] | 2014 | India | Prospective case-control study | Small bowel aspirate | ·· | ·· | ·· | ·· | Colony count ≥ 10^5^ cfu/mL in quantitative upper gut aspirate culture | 82 | 15 | 18·3% | Rome III |

| Author | Year | Country | Study design | Mode of diagnosis of SIBO | Substrate used for HBT | Dose of substrate for HBT | Test duration | Sample intervals of HBT | Cut-off criteria for diagnosis of SIBO | Total number of IBS patients | Number of IBS patients with SIBO | Crude pooled rate for SIBO | Diagnostic criteria used for IBS |
| --- | --- | --- | --- | --- | --- | --- | --- | --- | --- | --- | --- | --- | --- |
| Zhao et al.[11] | 2014 | China | Prospective case-control study | HBT | Lactulose, labelled with 37 MBq ^99m^Tc-diethylene triamine pentaacetic acid | 10 g in 100 mL of water | 3 h | 15 min | Criteria 1: A H_2_ rise of ≥ 20 ppm within 180 min  Criteria 2: A H_2_ rise of ≥ 20 ppm within 90 min  Criteria 3: Dual breath H_2_ peaks, a 12-ppm increase in breath H_2_ over baseline with a decrease in 5 ppm before the second peak, or  Criteria 4: Initial H_2_ rise, involving ≥ 2 consecutive values ≥ 5 ppm above baseline, commenced at least 15 min before an increase in radioactivity (≥ 5% of administered dose) in the caecal region, or  Criteria 5: Initial H_2_ rise, involving ≥ 2 consecutive values ≥ 10 ppm above baseline, commenced at least 15 min before an increase in radioactivity (≥5% of administered dose) in the caecal region, or   Criteria 6: Initial H_2_ rise, involving ≥ 2 consecutive values ≥ 20 ppm above baseline, commenced at least 15 min before an increase in radioactivity (≥ 5% of administered dose) in the caecal region | 89 | Criteria1: 67  Criteria 2: 28  Criteria 3: 39  Criteria 4: 35  Criteria 5: 15  Criteria 6: 3 | Criteria1: 75·2%  Criteria 2: 31·5%  Criteria 3: 43·8%  Criteria 4: 39·3%  Criteria 5: 16·9%  Criteria 6: 3·4% | Rome III |
| Abbasi et al.[116] | 2015 | Iran | Prospective case-control study | HBT | Glucose | 1g/kg of glucose in 150 mL of water | 100 min | 20 min | H_2_ levels > 20 ppm when the baseline was < 10 ppm, or  Increased by > 12 ppm when the baseline was ≥ 10 ppm | 107 | 40 | 37·4% | Rome III |
| Lasa et al.[19] | 2015 | Argentina | Prospective case-control study | HBT | Lactulose | 6·7 g | 2.5 h | 20 min | H_2_ excretion > 20 ppm before 90 min, or  An initial peak of 12 ppm before 90 min followed by a decrease of 5 ppm and a later increase (double H_2_ peak), and  An increase in CH_4_ excretion > 3 ppm at any time during the test | 15 | 10 | 66·7% | Rome III |
| Soldi et al.[20] | 2015 | Italy | Cross-sectional study | HBT | Lactulose | Not reported | Not reported | Not reported | Not reported | 15 | 12 | 80·0% | Rome II |
| Chu et al.[117] | 2016 | China | Prospective case-control study | HBT | Lactulose, labelled with 35 MBq ^99m^Tc-diethylene triamine pentaacetic acid | 10 g in 100 mL of water | 3 h | 15 min | An initial H_2_ increase involving at least two consecutive values of ≥5 ppm above baseline, beginning at least 15 min before an increase in radioactivity (≥ 5% of administered dose) in the caecal region | 89 | 35 | 39·3% | Rome III |
| Nagasako et al.[118] | 2016 | Brazil | Cross-sectional study | HBT | Glucose | 50 g | 2 h | 10 min | An increase over the baseline H_2_ level > 12 ppm | 84 | 13 | 15·5% | Rome III |
| Ning et al.[119] | 2016 | Japan | Prospective case-control study | HBT | Glucose | 50 g in 300 mL of water | 3 h | 15 min | The basal value was < 10 ppm and the subtracted peak value above basal was ≥ 10 ppm at 60 - 120 min after glucose loading | 10 | 0 | 0·0% | Rome III |

| Author | Year | Country | Study design | Mode of diagnosis of SIBO | Substrate used for HBT | Dose of substrate for HBT | Test duration | Sample intervals of HBT | Cut-off criteria for diagnosis of SIBO | Total number of IBS patients | Number of IBS patients with SIBO | Crude pooled rate for SIBO | Diagnostic criteria used for IBS |
| --- | --- | --- | --- | --- | --- | --- | --- | --- | --- | --- | --- | --- | --- |
| Ding et al.[21] | 2017 | China | Prospective case-control study | HBT | Lactulose | 6·7 g | 2.5 h | 20 min | H_2_ in fasting breath ≥ 20 ppm, mean H_2_ level of duplicate measurements at an interval of 20 min ≥ 20 ppm, or  Classical dual peaks, i.e. small intestine peak and colon peak were seen after oral administrated of lactulose. The level of H_2_ peak was higher than fasting level by 12 ppm, with a starting point before 90 min, and another higher peak appeared 60 min later, or  After oral administrated of lactulose, a high flat H_2_ peak appeared as a starting point before 90 min, and the highest level was higher than fasting level by 20 ppm within 180 min, or  An increase in H_2_ ≥ 20 ppm 90 min after oral administrated of lactulose, or  CH_4_ in fasting breath ≥ 10 ppm, mean CH_4_ level of duplicate measurements at an interval of 20 min ≥ 10 ppm, or  After oral administrated of lactulose, a high flat CH_4_ peak appeared as a starting point before 90 min, and the highest level was higher than fasting level by 10 ppm within 180 min | 50 | 36 | 72·0% | Rome III |
| Jung et al.[120] | 2017 | Korea | Retrospective case-control study | HBT | Lactulose | 10 g of in 200 mL of water | 2 h | 20 min for the first hour, then 15 min for the second hour | A baseline value of H_2_ ≥ 20 or CH_4_ ≥ 10 ppm, or/and  A > 20 ppm increase in H_2_, or   ≥ 10 in CH_4_ over the baseline within 90 minutes | 485 | 158 | 32·6% | Rome III |
| Moraru et al.[28] | 2017 | Romania | Prospective case-control study | HBT | Glucose | 50 g in 250 mL of water | 2 h | 15 min | A clear H_2_ peak > 20 ppm before the 120 min | 30 | 2 | 6·7% | Rome III |
| Jung et al.[8] | 2018 | Korea | Prospective case-control study | HBT | Glucose | 75 g | 3 h | 20 min | H_2_ or CH_4_ peak concentrations > 12 ppm if baseline levels were < 10 ppm, or  An increase in H_2_ or CH_4_ peak concentrations > 12 ppm if baseline levels were > 10 ppm | 198 | 96 | 48·5% | Rome III |
| Lee et al.[22] | 2018 | Korea | Cross-sectional study | HBT | Lactulose | 10 g | 1.5 h | 20 min | A baseline value of H_2_ > 20 ppm, and/or  An increase in H_2_ above the baseline value of > 20 ppm between 15 and 90 min after lactulose ingestion | 11 | 6 | 54·5% | Rome III |
| Huang et al.[121] | 2019 | China | Prospective case-control study | HBT | Lactulose | 10 g in 200 mL of water | 2.5 h | 20 min | A baseline value of H_2_ > 20 ppm with a continuous increase in H_2_ content, or  An increase in H_2_ above the baseline value of > 20 ppm within 90 min, or  A baseline value of CH_4_ > 10 ppm with a continuous increase in CH_4_ content, or  An increase in baseline value of CH_4_ > 10 ppm | 76 | 57 | 75·0% | Rome III |
| Lee et al.[122] | 2019 | Korea | Cross-sectional study | HBT | Lactulose | 10 g in 20 mL of water | 1.5 h | 20 min | A baseline value of H_2_ > 20 ppm, and/or  An increase in H_2_ above the baseline value of > 20 ppm between 15 and 90 min after lactulose ingestion | 475 | 206 | 43·4% | Rome III |
| Leventogiannis et al.[123] | 2019 | Greece | Prospective case-control study | Small bowel aspirate | ·· | ·· | ·· | ·· | Colony count ≥ 10^5^ cfu/mL and/or presence of colonic type bacteria in the duodenal aspirate | 143 | 6 | 4·2% | Rome III |
| Rezaie et al.[124] | 2019 | US | Prospective case-control study | HBT | Lactulose | 10 g in 120 mL of water | up to 2 h | 20 min | H_2_ rise of > 20 ppm within 90 min, and/or  CH_4_ of ≥ 10 ppm at any timepoint | 93 | 62 | 66·7% | Rome III |
| Wu et al.[125] | 2019 | China | Prospective case-control study | HBT | Lactulose | 10 g | 105 min | 15 min | A baseline value of H_2_ ≥ 20 ppm, or  CH_4_ of ≥ 10 ppm, or  20 ppm increase in H_2_ or ≥ 10 ppm increase in CH_4_ above the baseline value within 90 min | 60 | 31 | 51·7% | Rome IV |

| Author | Year | Country | Study design | Mode of diagnosis of SIBO | Substrate used for HBT | Dose of substrate for HBT | Test duration | Sample intervals of HBT | Cut-off criteria for diagnosis of SIBO | Total number of IBS patients | Number of IBS patients with SIBO | Crude pooled rate for SIBO | Diagnostic criteria used for IBS |
| --- | --- | --- | --- | --- | --- | --- | --- | --- | --- | --- | --- | --- | --- |
| Li et al.[29] | 2020 | China | Prospective case-control study | HBT | Glucose | 1 g per each kg of body weight (max. 60 g) | 2 h | 20 min | An increase of H_2_ > 10 ppm from baseline | 30 | 8 | 26·7% | Rome III |
| Sundin et al.[30] | 2020 | Sweden | Prospective case-control study | HBT  Small bowel aspirate | Lactulose  Glucose  ·· | 10 g in 300 mL of water  45 g in 300 mL of water  ·· | 2.5 h  2 h  ·· | 15 min  15 min  ·· | Fasting H_2_ or CH_4_ of ≥ 15 ppm, or  Rise of H_2_ or CH_4_ of ≥ 20 ppm above baseline within 90 min, or  (for lactulose) Two distinct peaks of H_2_ or CH_4_ of > 20 ppm  ≥ 10^5^ cfu/mL anaerobic mixed bacterial flora | 17 | Lactulose HBT:  5  Glucose HBT:  5  Small bowel aspirate:  0 | Lactulose HBT:  41·7%  Glucose HBT:  41·7%  Small bowel aspirate:  0% | Rome III |
| Yang et al.[31] | 2020 | China | Prospective case-control study | HBT | Glucose | 1 g per each kg of body weight (max. 60 g) | 2 h | 20 min | Basal H_2_ > 10 ppm, or  An increase of H_2_ > 12 ppm above baseline | 77 | 34 | 44·2% | Rome III |
| Zhuang et al.[126] | 2020 | China | Cross-sectional study | HBT | Lactulose | 10 g in 100 mL of water | 3 h | 30 min | A basal value of H_2_ ≥ 20 ppm, or  A > 20 ppm increase in H_2_ over basal values within 90 min | 78 | 45 | 57·7% | Rome IV |

CH_4_: methane; cfu: colony-forming unit; H_2_: hydrogen; HBT: hydrogen breath test; ppm: parts per million

**Supplementary table 16. Summary of the studies that reported the prevalence of BAD in patients fulfilling IBS criteria and healthy controls.**

| Author | Diagnostic criteria for BAD | Total number of IBS patients | Number of IBS patients with BAD | Crude pooled rate of BAD in IBS patients | Total number of healthy controls | Number of healthy controls with BAD | Crude pooled rate of BAD in healthy controls | Diagnostic criteria used for IBS |
| --- | --- | --- | --- | --- | --- | --- | --- | --- |
|  |  |  |  |  |  |  |  |  |
| Bajor et al.[37] | 7-day SeHCAT <15% | 141 | 45 | 31·9% | 29 | 0 | 0% | Rome III |
| Jeffery et al.[40] | 7-day SeHCAT <15% | 45 | 18 | 40·0% | 9 | 1 | 11·1% | Rome IV |
| Vijayvargiya et al.[44] | 48-hour faecal bile acids >2,337 μmol | 938 | 69 | 7·4% | 30 | 0 | 0% | Rome III |
| Dior et al.[46] | 7α-C4 >13·63 ng/mL | 15 | 5 | 33·3% | 12 | 0 | 0% | Rome III |

**Supplementary table 17. Summary of the studies that reported the prevalence of lactose malabsorption +/- intolerance based upon a positive hydrogen breath test in patients fulfilling IBS criteria and healthy controls.**

| Author | Dose of lactose for HBT | Test duration of HBT | Sample intervals of HBT | Cut-off criteria for diagnosis of lactose malabsorption | Total number of IBS patients | Number of IBS patients with lactose malabsorption | Crude pooled rate for lactose malabsorption in IBS patients | Number of IBS patients with lactose intolerance | Crude pooled rate for lactose intolerance in IBS patients | Total number of healthy controls | Number of healthy controls with lactose malabsorption | Crude pooled rate of lactose malabsorption in healthy controls | Number of healthy controls with lactose intolerance | Crude pooled rate of lactose intolerance in healthy controls | Diagnostic criteria used for IBS |
| --- | --- | --- | --- | --- | --- | --- | --- | --- | --- | --- | --- | --- | --- | --- | --- |
| Böhmer et al.[48] | 50 g in 100 mL of water | 1.5 h | at 15, 30, 60, 120, 150 and 180 min | An increase of H_2_ ≥ 20 ppm above the basal value, and a positive glucose blood test, defined as a flat curve with a maximum rise in glucose of 1·1 mmol/L | 70 | 17 | 24·3% | ·· | ·· | 35 | 2 | 5·7% | ·· | ·· | Manning |
| Farup et al.[54] | 25 g | 1.5 h | at 30, 60, 120 and 180 min | An increase of H2 >20 ppm above the lowest preceding value, or peak CH4 excretion >12 ppm above baseline, or an increase of combined H2 and CH4 >15 ppm | 74 | 3 | 4·1% | ·· | ·· | 105 | 4 | 3·8% | ·· | ·· | Rome II |
| Gupta et al.[25] | 50 g of lactose in 200 mL of water | 4 h | 15 min | A persistent rise in H2 of ≥ 12 ppm and ≥ 20 pm above basal level in ≥ 2 samples | 124 | ·· | ·· | 89 | 71·7% | 53 | ·· | ·· | 32 | 60·4% | Rome II |
| Yang et al.[3] | 10 g, 20 g, and 40 g in 250 mL of water | 3 h | 15 min | An increase of H_2_ > 20 ppm above the basal value on ≥ 2 consecutive readings | 60 | 10 g: 25  20 g: 52  40 g: 56 | 10 g: 41·6%  20 g: 86·7%  40 g: 93·3% | 10 g: 11  20 g: 28  40 g: 51 | 10 g: 18·3%  20 g: 46·7%  40 g: 85·0% | 60 | 10 g: 21  20 g: 48  40 g: 55 | 10 g: 35·0%  20 g: 80·0%  40 g: 91·7% | 10 g: 2  20 g: 13  40 g: 41 | 10 g: 3·3%  20 g: 21·7%  40 g: 68·3% | Rome III |
| Zhu et al.[61] | 20 g | 3 h | 15 min | An increase of H_2_ > 20 ppm above the basal value on ≥ 2 consecutive readings | 277 | 211 | 76·2% | 149 | 53·8% | 64 | 48 | 75·0% | 18 | 28·1% | Rome III |
| Yang et al.[64] | 20 g | 3 h | 15 min | An increase of H_2_ ≥ 20 ppm on ≥ 2 consecutive readings | 55 | 48 | 87·3% | 25 | 45·5% | 18 | 16 | 88·9% | 3 | 16·7% | Rome III |
| Dabak et al.[66] | 25 g in 250 mL of water | 2 h | at 15, 30, 60, 90 and 120 min | An increase of H2 ≥ 20 ppm on ≥ 2 samples within 2 h | 100 | 47 | 47·0% | ·· | ·· | 100 | 23 | 23·0% | ·· | ·· | Rome III |
| Xiong et al.[67] | 25 g in 100 mL of water | 3 h | 15 min | An increase of H2 > 20 ppm above the basal value on ≥ 2 samples within 3 h | 96 | 82 | 85·4% | 43 | 44·8% | 47 | 34 | 72·3% | 8 | 17·0% | Rome III |

**Supplementary table 18. Summary of the studies that reported the prevalence of lactose intolerance based upon genotyping studies in patients fulfilling IBS criteria and healthy controls.**

| Author | Total number of IBS patients | Number of IBS patients with C/C-13910 | Crude pooled rate for C/C-13910 in IBS patients | Number of IBS patients with G/G-22018 | Crude pooled rate for G/G-22018 in IBS patients | Total number of healthy controls | Number of healthy controls with C/C-13910 | Crude pooled rate of C/C-13910 in healthy controls | Number of healthy controls with G/G-22018 | Crude pooled rate of G/G-22018 in healthy controls | Diagnostic criteria used for IBS |
| --- | --- | --- | --- | --- | --- | --- | --- | --- | --- | --- | --- |
| Bernardes-Silva et al.[57] | 72 underwent genotyping for C/C-13910 75 underwent genotyping for G/G-22018 | 34 | 47·2% | 37 | 49·3% | 74 | 37 | 50·0% | 38 | 51·4% | Rome II |
| Kumar et al.[68] | 150 | 102 | 68·0% | 97 | 64·7% | 252 | 155 | 61·5% | 154 | 61·1% | Rome III |
| Yang et al.[69] | 60 | 60 | 100·0% | ·· | ·· | 60 | 60 | 100·0% | ·· | ·· | Rome III |

**Supplementary table 19. Summary of the studies that reported the prevalence of fructose malabsorption based upon a positive hydrogen breath test in patients fulfilling IBS criteria and healthy controls.**

| Author | Dose of lactose for HBT | Test duration of HBT | Sample intervals of HBT | Cut-off criteria for diagnosis of fructose malabsorption | Total number of IBS patients | Number of IBS patients with fructose malabsorption | Crude pooled rate for fructose malabsorption in IBS patients | Total number of healthy controls | Number of healthy controls with fructose malabsorption | Crude pooled rate of fructose malabsorption in healthy controls | Diagnostic criteria used for IBS |
| --- | --- | --- | --- | --- | --- | --- | --- | --- | --- | --- | --- |
| Sharma et al.[10] | 25 g in 250 mL of water | 3 h | 15 min | A sustained rise of H_2_ > 20 ppm above the baseline value | 97 | 14 | 14·4% | 41 | 1 | 2·4% | Rome III |
| Jung et al.[8] | 15 g and 25 g in 250 mL of water | 3 h | 30 min | A sustained rise of H2 > 20 ppm over the baseline value, or CH4 > 10 ppm, or H2 and CH4 combined > 15 ppm, or a sustained rise in H2 and/or CH4 of ≥ 5 ppm on ≥ 3 consecutive samples | 35 | 15 g: 7  25 g: 16 | 15 g: 20·0%  25 g: 45·7% | 35 | 15 g: 2  25 g: 8 | 15 g: 5·7%  25 g: 22·9% | Rome III |

CH_4_: methane; H_2_: hydrogen; HBT: hydrogen breath test; ppm: parts per million

**Supplementary table 20. Summary of the studies that reported the prevalence of MC in patients fulfilling IBS criteria and healthy controls.**

| Author | Total number of IBS patients with MC | Number of IBS patients with MC | Crude pooled rate of MC in IBS patients | Total number of healthy controls | Number of healthy controls with MC | Crude pooled rate of MC in healthy controls | Diagnostic criteria used for IBS |
| --- | --- | --- | --- | --- | --- | --- | --- |
| Chey et al.[79] | 466 | 7 | 1·5% | 451 | 0 | 0% | Rome II |
| Ozdil et al.[81] | 226 | 7 | 3·1% | 152 | 0 | 0% | Rome III |
| Hilmi et al.[83] | 74 | 1 | 1·4% | 46 | 0 | 0% | Rome III |

**Supplementary table 21. Summary of the studies that reported the prevalence of SIBO based upon a positive lactulose hydrogen breath test in patients fulfilling IBS criteria and healthy controls.**

| Author | Dose of lactulose for HBT | Test duration | Sample intervals of HBT | Cut-off criteria for diagnosis of SIBO | Total number of IBS patients | Number of IBS patients with SIBO | Crude pooled rate for SIBO in IBS patients | Total number of healthy controls | Number of healthy controls with SIBO | Crude pooled rate of SIBO in healthy controls | Diagnostic criteria used for IBS |
| --- | --- | --- | --- | --- | --- | --- | --- | --- | --- | --- | --- |
| Pimentel et al.[96] | 10 g | 3 h | 15 min | A rise of breath H_2_ or CH_4_ > 20 ppm before 90 min during 180 min | 111 | 93 | 83·8% | 15 | 3 | 20·0% | Rome |
| Jung et al.[13] | 10 g of in 100 mL of water | 3 h | 15 min | A peak of H2 and/or CH4 >20 ppm that occurred 15 min before the colonic peak, or   An elevated fasting H2 and/or CH4 >12-15 ppm | 38 | 7 | 18·4% | 12 | 1 | 8·3% | Rome II |
| Koza et al.[14] | 10 g in 400 mL of water | 30 min | 15 min | A rise in H_2_ ≥ 12 ppm above baseline level | 44 | 7 | 15·9% | 28 | 0 | 0% | Rome III |
| Rana et al.[17]* | 10 g | 4 h | 30 min | A H2 or CH4 peak > 20 ppm over the baseline value in ≥ 2 samples, or  A concentration of CH4 > 10 ppm | 345 | 17 | 4·9% | 254 | 52 | 20·5% | Rome II |
| Youn et al.[109] | 10 g in 200 mL of water | 3 h | 15 min | H2 > 20 ppm at baseline, or   An increase of H2 > 20 ppm over baseline within 90 min, or  CH4 > 10 ppm at baseline, or  An increase of CH4 > 10 ppm over baseline within 90 min | 84 | 46 | 54·8% | 25 | 8 | 32·0% | Rome II |
| Rana et al.[111] | 10 g | 2.5 h | 10 min | Sustained increase in breath concentration of H2 or CH4 or both ≥ 10 ppm over a baseline value within 90 min | 175 | 60 | 34·2% | 150 | 45 | 30·0% | Rome II |
| Lee et al.[112] | 10 g in 200 mL of water | 2.5 h | 15 min | A rise in H_2_ > 20 ppm or CH_4_ > 10 ppm within 90 min | 68 | 42 | 61.8% | 55 | 21 | 38·2% | Rome III |

| Author | Dose of lactulose for HBT | Test duration | Sample intervals of HBT | Cut-off criteria for diagnosis of SIBO | Total number of IBS patients | Number of IBS patients with SIBO | Crude pooled rate for SIBO in IBS patients | Total number of healthy controls | Number of healthy controls with SIBO | Crude pooled rate of SIBO in healthy controls | Diagnostic criteria used for IBS |
| --- | --- | --- | --- | --- | --- | --- | --- | --- | --- | --- | --- |
| Zhao et al.[11] | 10 g in 100 mL of water | 3 h | 15 min | Criteria 1: A H2 rise of ≥ 20 ppm within 180 min  Criteria 2: A H2 rise of ≥ 20 ppm within 90 min  Criteria 3: Dual breath H2 peaks, a 12-ppm increase in breath H2 over baseline with a decrease in 5 ppm before the second peak, or  Criteria 4: Initial H2 rise, involving ≥ 2 consecutive values ≥ 5 ppm above baseline, commenced at least 15 min before an increase in radioactivity (≥ 5% of administered dose) in the caecal region, or  Criteria 5: Initial H2 rise, involving ≥ 2 consecutive values ≥ 10 ppm above baseline, commenced at least 15 min before an increase in radioactivity (≥5% of administered dose) in the caecal region, or   Criteria 6: Initial H2 rise, involving ≥ 2 consecutive values ≥ 20 ppm above baseline, commenced at least 15 min before an increase in radioactivity (≥ 5% of administered dose) in the caecal region | 89 | Criteria1: 67  Criteria 2: 28  Criteria 3: 39  Criteria 4: 35  Criteria 5: 15  Criteria 6: 3 | Criteria1: 75·2%  Criteria 2: 31·5%  Criteria 3: 43·8%  Criteria 4: 39·3%  Criteria 5: 16·9%  Criteria 6: 3·4% | 13 | Criteria1: 10  Criteria 2: 4  Criteria 3: 5  Criteria 4: 1  Criteria 5: 0  Criteria 6: 0 | Criteria1: 76·9%  Criteria 2: 30·8%  Criteria 3: 38·5%  Criteria 4: 7·7%  Criteria 5: 0%  Criteria 6: 0% | Rome III |
| Lasa et al.[19] | 6·7 g | 2.5 h | 20 min | H2 excretion > 20 ppm before 90 min, or  An initial peak of 12 ppm before 90 min followed by a decrease of 5 ppm and a later increase (double H2 peak), and  An increase in CH4 excretion > 3 ppm at any time during the test | 15 | 10 | 66·7% | 15 | 2 | 13·3% | Rome III |
| Chu et al.[117] | 10 g in 100 mL of water | 3 h | 15 min | An initial H_2_ increase involving at least two consecutive values of ≥5 ppm above baseline, beginning at least 15 min before an increase in radioactivity (≥ 5% of administered dose) in the caecal region | 89 | 35 | 39·3% | 13 | 1 | 7·7% | Rome III |
| Author | Dose of lactulose for HBT | Test duration | Sample intervals of HBT | Cut-off criteria for diagnosis of SIBO | Total number of IBS patients | Number of IBS patients with SIBO | Crude pooled rate for SIBO in IBS patients | Total number of healthy controls | Number of healthy controls with SIBO | Crude pooled rate of SIBO in healthy controls | Diagnostic criteria used for IBS |
| Ding et al.[21] | 6·7 g | 2.5 h | 20 min | H2 in fasting breath ≥ 20 ppm, mean H2 level of duplicate measurements at an interval of 20 min ≥ 20 ppm, or  Classical dual peaks, i.e. small intestine peak and colon peak were seen after oral administrated of lactulose. The level of H2 peak was higher than fasting level by 12 ppm, with a starting point before 90 min, and another higher peak appeared 60 min later, or  After oral administrated of lactulose, a high flat H2 peak appeared as a starting point before 90 min, and the highest level was higher than fasting level by 20 ppm within 180 min, or  An increase in H2 ≥ 20 ppm 90 min after oral administrated of lactulose, or  CH4 in fasting breath ≥ 10 ppm, mean CH4 level of duplicate measurements at an interval of 20 min ≥ 10 ppm, or  After oral administrated of lactulose, a high flat CH4 peak appeared as a starting point before 90 min, and the highest level was higher than fasting level by 10 ppm within 180 min | 50 | 36 | 72·0% | 50 | 19 | 38·0% | Rome III |
| Huang et al.[121] | 10 g in 200 mL of water | 2.5 h | 20 min | A baseline value of H2 > 20 ppm with a continuous increase in H2 content, or  An increase in H2 above the baseline value of > 20 ppm within 90 min, or  A baseline value of CH4 > 10 ppm with a continuous increase in CH4 content, or  An increase in baseline value of CH4 > 10 ppm | 76 | 57 | 75·0% | 30 | 11 | 36·7% | Rome III |
| Wu et al.[125] | 10 g | 105 min | 15 min | A baseline value of H2 ≥ 20 ppm, or   CH4 of ≥ 10 ppm, or  20 ppm increase in H2 or ≥ 10 ppm increase in CH4 above the baseline value within 90 min | 60 | 31 | 51·7% | 60 | 10 | 16·7% | Rome IV |
| Author | Dose of lactulose for HBT | Test duration | Sample intervals of HBT | Cut-off criteria for diagnosis of SIBO | Total number of IBS patients | Number of IBS patients with SIBO | Crude pooled rate for SIBO in IBS patients | Total number of healthy controls | Number of healthy controls with SIBO | Crude pooled rate of SIBO in healthy controls | Diagnostic criteria used for IBS |
| Sundin et al.[30] | 10 g in 300 mL of water | 2.5 h | 15 min | Fasting H2 or CH4 of ≥ 15 ppm, or  Rise of H2 or CH4 of ≥ 20 ppm above baseline within 90 min, or  Two distinct peaks of H2 or CH4 of > 20 ppm | 17 | 5 | 41·7% | 20 | 9 | 45·0% | Rome III |

CH_4_: methane; H_2_: hydrogen; HBT: hydrogen breath test; ppm: parts per million

*only methane level was measured during HBT in this study

**Supplementary table 22. Summary of the studies that reported the prevalence of SIBO based upon a positive glucose hydrogen breath test in patients fulfilling IBS criteria and healthy controls.**

| Author | Dose of glucose for HBT | Test duration | Sample intervals of HBT | Cut-off criteria for diagnosis of SIBO | Total number of IBS patients | Number of IBS patients with SIBO | Crude pooled rate for SIBO in IBS patients | Total number of healthy controls | Number of healthy controls with SIBO | Crude pooled rate of SIBO in healthy controls | Diagnostic criteria used for IBS |
| --- | --- | --- | --- | --- | --- | --- | --- | --- | --- | --- | --- |
| Ghoshal et al.[24] | 100 g in 200 mL of water | 3 h | 10 min | Rise of breath H2 by 14 ppm above basal level following glucose administration | 12 | 0 | 0% | 12 | 0 | 0% | Rome |
| Lupascu et al.[97] | 50 g | 2 h | 10 min | An increase over the baseline of H_2_ level > 12 ppm | 65 | 20 | 30·8% | 102 | 4 | 3·9% | Rome II |
| Rana et al.[127] | 50 g in 200 mL of water | 2 h | 15 min | An increase in breath H_2_ > 12 ppm over baseline value | 225 | 25 | 11·1% | 100 | 1 | 1·0% | Rome II |
| Lombardo et al.[105] | 50 g in 250 mL of water | 2 h | 15 min | An increase over the baseline of H_2_ level > 10 ppm | 200 | 49 | 24·5% | 50 | 3 | 6·0% | Rome III |
| Sachdeva et al.[108] | 100 g in 200 mL of water | 3 h | 15 min | A persistent rise in breath H2 or CH4 > 12 ppm above basal on ≥ 2 readings, or  A fasting CH4 concentration of > 10 ppm | 59 | 14 | 23·7% | 37 | 1 | 2·7% | Rome III |
| Rana et al.[111] | 80 g | 2.5 h | 10 min | Sustained increase in breath concentration of H2 or CH4 or both ≥ 10 ppm over a baseline value within 120 min | 175 | 11 | 6·2% | 150 | 1 | 0·7% | Rome II |
| Abbasi et al.[116] | 1g/kg of glucose in 150 mL of water | 100 min | 20 min | H2 levels > 20 ppm when the baseline was < 10 ppm, or  Increased by > 12 ppm when the baseline was ≥ 10 ppm | 107 | 40 | 37·4% | 107 | 14 | 13·1% | Rome III |
| Jung et al.[8] | 75 g | 3 h | 20 min | H2 or CH4 peak concentrations > 12 ppm if baseline levels were < 10 ppm, or  An increase in H2 or CH4 peak concentrations > 12 ppm if baseline levels were > 10 ppm | 198 | 96 | 48·5% | 38 | 2 | 5·3% | Rome III |
| Sundin et al.[30] | 45 g in 300 mL of water | 2 h | 15 min | Fasting H2 or CH4 of ≥ 15 ppm, or  Rise of H2 or CH4 of ≥ 20 ppm above baseline within 90 min | 17 | 5 | 41·7% | 20 | 8 | 40·0% | Rome III |

CH_4_: methane; H_2_: hydrogen; HBT: hydrogen breath test; ppm: parts per million

**Supplementary table 23. Summary of the studies that reported the prevalence of SIBO based upon small bowel aspirate in patients fulfilling IBS criteria and healthy controls.**

| Author | Cut-off criteria for diagnosis of SIBO | Total number of IBS patients | Number of IBS patients with SIBO | Crude pooled rate for SIBO in IBS patients | Total number of healthy controls | Number of healthy controls with SIBO | Crude pooled rate of SIBO in healthy controls | Diagnostic criteria used for IBS |
| --- | --- | --- | --- | --- | --- | --- | --- | --- |
| Posserud et al.[101] | > 10^5^ cfu/mL of colonic bacteria | 162 | 7 | 4·3% | 26 | 1 | 3.8% | Rome II |
| Sundin et al.[30] | ≥ 10^5^ cfu/mL anaerobic mixed bacterial flora | 17 | 0 | 0% | 20 | 0 | 0% | Rome III |

CH_4_: methane; cfu: colony-forming unit; H_2_: hydrogen; HBT: hydrogen breath test; ppm: parts per million

**Supplementary table 24. The prevalence rates of conditions in patients fulfilling IBS criteria, compared to those in healthy controls.**

| Condition | Diagnostic test for the condition | Mean rate in IBS | Mean rate in healthy controls | p-value |
| --- | --- | --- | --- | --- |
| BAD | 7-day SeHCAT <15% | 33·9% (95% CI 27·1-40·7%) | 2·6% (95% CI 0-7·7%) | p =0·0001 |
| Lactose malabsorption | Lactose HBT | 62·8% (95% CI 59·3-66·3%)* | 40·8% (95% CI 36·1-45·4%)* | p <0·0001 |
| Lactose intolerance | Lactose HBT + onset of abdominal symptoms during HBT  Genotyping for C/C-13910  Genotyping for G/G-22018 | 54·6% (95% CI 50·6-58·1%)*  69·5% (95% CI 64·1-74·9%)  59·6% (95% CI 53·1-66·0%) | 30·6% (95% CI 24·8-36·4%)*  65·3% (95% CI 60·5-70·0%)  58·9% (95% CI 53·6-64·2%) | p <0·0001  p =0·25  p =0·88 |
| Fructose malabsorption | Fructose HBT | 22·7% (95% CI 15·6-29·9%) | 11·8% (95% CI 4·6-19·1%) | p =0·005 |
| MC | Histological examination of colonic biopsy | 2·0% (95% CI 1·0-2·9%) | 0% | p =0·0003 |
| SIBO | Lactulose HBT  Glucose HBT  Small bowel aspirate | 40·7% (95% CI 38·0-43·4%)**  24·6% (95% CI 22·0-27·2%)  3·9% (95% CI 1·1-6·8%) | 25·9% (95% CI 22·8-29·1%)**  5·5% (95% CI 3·7-7·3%)  2·2% (95% CI 0-6·3%) | p <0·0001  p <0·0001  p = 0·57 |

CI: confidence interval; HBT: hydrogen breath test

*Prevalence rate calculated based on 20g lactose used in the HBT by Yang et al.[3]

** Prevalence rate calculated based on diagnostic criteria 1 adopted by Zhao et al.[11]

**Supplementary table 25. Advantages and disadvantages of diagnostic tests used to identify the five GI conditions.**

| Organic GI conditions | Diagnostic tests | Advantages | Disadvantages |
| --- | --- | --- | --- |
| BAD | SeHCAT scan | Non-invasive and well tolerated | Relatively expensive |
|  |  | High sensitivity and specificity | Not available in some countries |
|  |  | Quantitative evaluation of BAD predicts response to bile acid sequestrant | Low dose radiation exposure |
|  |  |  | Requires 2 patient visits |
|  | 48-hour faecal bile acids | Non-invasive | Variable daily faecal bile acid excretion |
|  |  | Measures total and individual bile acids | Cumbersome |
|  |  |  |  |
|  | Fasting serum 7α-C4 | Non-invasive | Requires a fasting sample |
|  |  | Not dependent on age, gender, or cholesterol | Diurnal variation |
|  |  |  | False positive in liver disease and treatment with statins |
|  |  |  | Requires further validation |
| CM | Breath test | Non-invasive | Lack of standardisation in pre-test preparation, conduction of test and interpretation of test results |
|  |  | Results immediately available | Can be affected by oro-caecal transit time and concurrent medication use |
|  |  |  | Low sensitivity and specificity |
|  |  |  | Poor test-retest reliability |
| MC | Colonic biopsies | High sensitivity and specificity | Expensive and invasive as lower GI endoscopy is required |
|  |  |  | Risks associated with endoscopy |
|  |  |  | Require multiple biopsies |
|  |  |  | Lack of agreement on the number of biopsies required |
|  |  |  | Skipped lesions |
|  |  |  | Require examination by a specially trained pathologist |
| PEI | Faecal elastase-1 | Non-invasive | Watery, loose stool can cause false positive result |
|  |  |  | Poor sensitivity at diagnosing mild to moderate PEI |
| SIBO | Small bowel aspirate | Successful culture of organisms can guide antibiotic treatment | Expensive and invasive as require lower GI endoscopy |
|  |  |  | Lack of agreement on aspirating technique |
|  |  |  | Contamination from oral bacteria is common |
|  |  |  | Only proximal small bowel fluid can be sampled |
|  |  |  |  |
|  | Breath test | Non-invasive | Lack of standardisation in pre-test preparation, conduction of test and interpretation of test results |
|  |  | Results immediately available | Methane is not always measured so methanogenic overgrowth can be missed |
|  |  |  | Can be affected by oro-caecal transit time and concurrent medication use |
|  |  |  | Low sensitivity and specificity |
|  |  |  | Poor test-retest reliability |

**References**

1. Fernandez-Banares, F. *et al.* Randomised clinical trial: colestyramine vs. hydroxypropyl cellulose in patients with functional chronic watery diarrhoea. *Aliment Pharmacol Ther*. **41** (11), 1132-40 (2015).

2. Ghoshal, U. C., Kumar, S., Misra, A.& Mittal, B. Lactose malabsorption diagnosed by 50-g dose is inferior to assess clinical intolerance and to predict response to milk withdrawal than 25-g dose in an endemic area. *J Gastroenterol Hepatol*. **28** (9), 1462-8 (2013).

3. Yang, J. *et al.* Prevalence and Presentation of Lactose Intolerance and Effects on Dairy Product Intake in Healthy Subjects and Patients With Irritable Bowel Syndrome. *Clinical Gastroenterology and Hepatology*. **11** (3), 262 (2013).

4. Corlew-Roath, M.& Di Palma, J. A. Clinical impact of identifying lactose maldigestion or fructose malabsorption in irritable bowel syndrome or other conditions. *Southern Medical Journal*. **102** (10), 1010-2 (2009).

5. Jafari, E., Vahedi, H., Merat, S., Momtahen, S.& Riahi, A. Therapeutic effects, tolerability and safety of a multi-strain probiotic in Iranian adults with irritable bowel syndrome and bloating. *Archives of iranian medicine*. **17** (7), 466‐70 (2014).

6. Almazar, A. E. *et al.* Comparison of Lactase Variant MCM6-13910 C>T Testing and Self-report of Dairy Sensitivity in Patients with Irritable Bowel Syndrome. *Journal of Clinical Gastroenterology*. **53** (6), 1-5 (2018).

7. Mack, A. *et al.* Changes in gut microbial metagenomic pathways associated with clinical outcomes after the elimination of malabsorbed sugars in an IBS cohort. *Gut Microbes*. **11** (3), 620-31 (2020).

8. Jung, K. W. *et al.* Prevalence of fructose malabsorption in patients with irritable bowel syndrome after excluding small intestinal bacterial overgrowth. *Journal of Neurogastroenterology and Motility*. **24** (2), 307-16 (2018).

9. Berg, L. K. *et al.* Effect of fructose-reduced diet in patients with irritable bowel syndrome, and its correlation to a standard fructose breath test. *Scandinavian Journal of Gastroenterology*. **48** (8), 936-43 (2013).

10. Sharma, A., Srivastava, D., Verma, A., Misra, A.& Ghoshal, U. C. Fructose malabsorption is not uncommon among patients with irritable bowel syndrome in India: A case-control study. *Indian Journal of Gastroenterology*. **33** (5), 466-70 (2014).

11. Zhao, J. *et al.* A study of the methodological and clinical validity of the combined lactulose hydrogen breath test with scintigraphic oro-cecal transit test for diagnosing small intestinal bacterial overgrowth in IBS patients. *Neurogastroenterol Motil*. **26** (6), 794-802 (2014).

12. Pimentel, M., Kong, Y.& Park, S. Breath Testing to Evaluate Lactose Intolerance in Irritable Bowel Syndrome Correlates with Lactulose Testing and May Not Reflect True Lactose Malabsorption. *American Journal of Gastroenterology*. **98** (12), 2700-4 (2003).

13. Jung, H. P. *et al.* The relationship between small-intestinal bacterial overgrowth and intestinal permeability in patients with irritable bowel syndrome. *Gut and Liver*. **3** (3), 174-9 (2009).

14. Koza, J., Meder, A.& Swiatkowski, M. Small intestinal bacterial overgrowth is diagnosed in some cases of irritable bowel syndrome. *Clinical and Experimental Medical Letters*. **50** (3), 197-8 (2009).

15. Mann, N. S.& Limoges-Gonzales, M. The prevalence of small intestinal bacterial overgrowth in irritable bowel syndrome. *Hepato-Gastroenterology*. **56** (91), 718-21 (2009).

16. Peralta, S., Cottone, C., Doveri, T., Almasio, P. L.& Craxi, A. Small intestine bacterial overgrowth and irritable bowel syndrome-related symptoms: experience with Rifaximin. *World J Gastroenterol*. **15** (21), 2628-31 (2009).

17. Rana, S. V. *et al.* Incidence of predominant methanogenic flora in irritable bowel syndrome patients and apparently healthy controls from North India. *Dig Dis Sci*. **54** (1), 132-5 (2009).

18. Hwang, L. *et al.* Evaluating breath methane as a diagnostic test for constipation-predominant IBS. *Dig Dis Sci*. **55** (2), 398-403 (2010).

19. Lasa, J. S., Zubiaurre, I., Fanjul, I., Olivera, P.& Soifer, L. Small intestinal bacterial overgrowth prevalence in celiac disease patients is similar in healthy subjects and lower in irritable bowel syndrome patients. *Rev Gastroenterol Mex*. **80** (2), 171-4 (2015).

20. Soldi, S. *et al.* Modulation of the gut microbiota composition by rifaximin in non-constipated irritable bowel syndrome patients: A molecular approach. *Clinical and Experimental Gastroenterology*. **8**, 309-25 (2015).

21. Ding, X. W. *et al.* The relationship between small intestinal bacterial overgrowth and irritable bowel syndrome. *Eur Rev Med Pharmacol Sci*. **21** (22), 5191-6 (2017).

22. Lee, S. H., Joo, N. S., Kim, K. M.& Kim, K. N. The therapeutic effect of a multistrain probiotic on diarrhea-predominant irritable bowel syndrome: A pilot study. *Gastroenterology Research and Practice*. **2018**, (2018).

23. Tuteja, A. K., Samore, M. H., Talley, N. J., Stoddard, G. J.& Verne, G. N. Risk factors for upper and lower functional gastrointestinal disorders in Persian Gulf War Veterans during and post-deployment. *Neurogastroenterology and Motility*. **31** (3), (2019).

24. Ghoshal, U. C. *et al.* Tropical sprue is associated with contamination of small bowel with aerobic bacteria and reversible prolongation of orocecal transit time. *Journal of Gastroenterology and Hepatology (Australia)*. **18** (5), 540-7 (2003).

25. Gupta, D., Ghoshal, U. C., Misra, A., Choudhuri, G.& Singh, K. Lactose intolerance in patients with irritable bowel syndrome from northern India: A case-control study. *Journal of Gastroenterology and Hepatology (Australia)*. **22** (12), 2261-5 (2007).

26. Stoicescu, A. *et al.* Microscopic colitis and small intestinal bacterial overgrowth--diagnosis behind the irritable bowel syndrome? *Rev Med Chir Soc Med Nat Iasi*. **116** (3), 766-72 (2012).

27. Moraru, I. G., Portincasa, P., Moraru, A. G., Diculescu, M.& Dumitrascu, D. L. Small intestinal bacterial overgrowth produces symptoms in irritable bowel syndrome which are improved by rifaximin. A pilot study. *Rom J Intern Med*. **51** (3-4), 143-7 (2013).

28. Moraru, I.& Dumitrascu, D. Colonoscopy does not induce small intestinal bacterial overgrowth. *Journal of Digestive Endoscopy*. **8** (1), 12-6 (2017).

29. Li, Y. *et al.* Fecal bacteria can predict the efficacy of rifaximin in patients with diarrhea-predominant irritable bowel syndrome. *Pharmacological Research*. **159**, (2020).

30. Sundin, J. *et al.* Evidence of altered mucosa-associated and fecal microbiota composition in patients with Irritable Bowel Syndrome. *Sci Rep*. **10** (1), 593 (2020).

31. Yang, M. *et al.* Duodenal and rectal mucosal microbiota related to small intestinal bacterial overgrowth in diarrhea-predominant irritable bowel syndrome. *J Gastroenterol Hepatol*. **35** (5), 795-805 (2020).

32. Galatola, G. *et al.* The prevalence of bile acid malabsorption in irritable bowel syndrome and the effect of cholestyramine: An uncontrolled open multicentre study. *European Journal of Gastroenterology and Hepatology*. **4** (7), 533-7 (1992).

33. Sinha, L., Liston, R., Moriarty, K. J.& Testa, H. J. Idiopathic bile acid malabsorption: Qualitative and quantitative clinical features a and response to cholestyramine. *Alimentary Pharmacology and Therapeutics*. **12** (9), 839-44 (1998).

34. Smith, M. J. *et al.* Bile acid malabsoprtion in persistent diarrhoea. *Journal of Royal College Physicians London*. **34** (5), 448-51 (2000).

35. Fernandez-Banares, F. *et al.* Systematic evaluation of the causes of chronic watery diarrhea with functional characteristics. *American Journal of Gastroenterology*. **102** (11), 2520-8 (2007).

36. Gracie, D. J. *et al.* Prevalence of, and predictors of, bile acid malabsorption in outpatients with chronic diarrhea. *Neurogastroenterology and Motility*. **24** (11), 983 (2012).

37. Bajor, A., Tornblom, H., Simren, M., Rudling, M.& Ung, K. A. Increased colonic bile acid exposure: A relevant factor for symptoms and treatment in IBS. *Gut*. **64** (1), 84-92 (2014).

38. Aziz, I. *et al.* High Prevalence of Idiopathic Bile Acid Diarrhea Among Patients With Diarrhea-Predominant Irritable Bowel Syndrome Based on Rome III Criteria. *Clin Gastroenterol Hepatol*. **13** (9), 1650-5.e2 (2015).

39. Fernandez-Banares, F. *et al.* Randomised clinical trial: Colestyramine vs. hydroxypropyl cellulose in patients with functional chronic watery diarrhoea. *Alimentary Pharmacology and Therapeutics*. **41** (11), 1132-40 (2015).

40. Jeffery, I. B. *et al.* Differences in Fecal Microbiomes and Metabolomes of People With vs Without Irritable Bowel Syndrome and Bile Acid Malabsorption. *Gastroenterology*. **158** (4), 1016-28.e8 (2020).

41. Shiha, M. G., Ashgar, Z., Fraser, E. M., Kurien, M.& Aziz, I. High prevalence of primary bile acid diarrhoea in patients with functional diarrhoea and irritable bowel syndrome-diarrhoea, based on Rome III and Rome IV criteria. *EClinicalMedicine*. **25**, (2020).

42. Camilleri, M. *et al.* Effect of increased bile acid synthesis or fecal excretion in irritable bowel syndrome-diarrhea. *The American journal of gastroenterology*. **109** (10), 1621-30 (2014).

43. Donato, L. J., Lueke, A., Kenyon, S. M., Meeusen, J. W.& Camilleri, M. Description of analytical method and clinical utility of measuring serum 7-alpha-hydroxy-4-cholesten-3-one (7aC4) by mass spectrometry. *Clinical Biochemistry*. **52**, 106-11 (2018).

44. Vijayvargiya, P. *et al.* Analysis of Fecal Primary Bile Acids Detects Increased Stool Weight and Colonic Transit in Patients With Chronic Functional Diarrhea. *Clinical Gastroenterology and Hepatology*. **17** (5), 922 (2018).

45. Vijayvargiya, P. *et al.* Effects of Colesevelam on Bowel Symptoms, Biomarkers, and Colonic Mucosal Gene Expression in Patients With Bile Acid Diarrhea in a Randomized Trial. *Clinical Gastroenterology and Hepatology*. (2020).

46. Dior, M. *et al.* Interplay between bile acid metabolism and microbiota in irritable bowel syndrome. *Neurogastroenterology and Motility*. **28** (9), 1330-40 (2016).

47. Vernia, P., Ricciardi, M. R., Frandina, C., Bilotta, T.& Frieri, G. Lactose malabsorption and irritable bowel syndrome. Effect of a long-term lactose-free diet. *Italian Journal of Gastroenterology*. **27** (3), 117-21 (1995).

48. Böhmer, C. J.& Tuynman, H. A. The clinical relevance of lactose malabsorption in irritable bowel syndrome. *European journal of gastroenterology & hepatology*. **8** (10), 1013‐6 (1996).

49. Hamm, L. R. *et al.* Additional investigations fail to alter the diagnosis of irritable bowel syndrome in subjects fulfilling the Rome Criteria. *American Journal of Gastroenterology*. **94** (5), 1279-82 (1999).

50. Goldstein, R., Braverman, D.& Stankiewicz, H. Carbohydrate malabsorption and the effect of dietary restriction on symptoms of irritable bowel syndrome and functional bowel complaints. *Isr Med Assoc J*. **2** (8), 583-7 (2000).

51. Parker, T. J. *et al.* Irritable bowel syndrome: is the search for lactose intolerance justified? *Eur J Gastroenterol Hepatol*. **13** (3), 219-25 (2001).

52. Vernia, P., Di Camillo, M.& Marinaro, V. Lactose malabsorption, irritable bowel syndrome and self-reported milk intolerance. *Digestive and Liver Disease*. **33** (3), 234-9 (2001).

53. Pimentel, M., Kong, Y.& Park, S. Breath testing to evaluate lactose intolerance in irritable bowel syndrome correlates with lactulose testing and may not reflect true lactose malabsorption. *Am J Gastroenterol*. **98** (12), 2700-4 (2003).

54. Farup, P. G., Monsbakken, K. W.& Vandvik, P. O. Lactose malabsorption in a population with irritable bowel syndrome: prevalence and symptoms. A case-control study. *Scand J Gastroenterol*. **39** (7), 645-9 (2004).

55. Vernia, P., Marinaro, V., Argnani, F., Di Camillo, M.& Caprilli, R. Self-reported milk intolerance in irritable bowel syndrome: What should we believe? *Clinical Nutrition*. **23** (5), 996-1000 (2004).

56. Nucera, G. *et al.* Abnormal breath tests to lactose, fructose and sorbitol in irritable bowel syndrome may be explained by small intestinal bacterial overgrowth. *Aliment Pharmacol Ther*. **21** (11), 1391-5 (2005).

57. Bernardes-Silva, C. F., Laudanna, A. A., Pereira, A. C., de Fatima Alves da Mota, G.& Krieger, J. E. Lactase persistence/non-persistence variants, C/T_13910 and G/A_22018, as a diagnostic tool for lactose intolerance in IBS patients. *Clinica Chimica Acta*. **386** (1), 7-11 (2007).

58. Ghoshal, U. C., Kumar, S., Chourasia, D.& Misra, A. Lactose hydrogen breath test versus lactose tolerance test in the tropics: does positive lactose tolerance test reflect more severe lactose malabsorption? *Tropical gastroenterology : official journal of the Digestive Diseases Foundation*. **30** (2), 86-90 (2009).

59. Yakoob, J. *et al.* Small intestinal bacterial overgrowth and lactose intolerance contribute to irritable bowel syndrome symptomatology in Pakistan. *Saudi Journal of Gastroenterology*. **17** (6), 371-5 (2011).

60. De Roest, R. H. *et al.* The low FODMAP diet improves gastrointestinal symptoms in patients with irritable bowel syndrome: A prospective study. *International Journal of Clinical Practice*. **67** (9), 895-903 (2013).

61. Zhu, Y. *et al.* Bloating and distention in irritable bowel syndrome: The role of gas production and visceral sensation after lactose ingestion in a population with lactase deficiency. *American Journal of Gastroenterology*. **108** (9), 1516-25 (2013).

62. Dainese, R. *et al.* Perception of lactose intolerance in irritable bowel syndrome patients. *European Journal of Gastroenterology & Hepatology*. **26** (10), 1167-75 (2014).

63. Goebel-Stengel, M. *et al.* Unclear abdominal discomfort: Pivotal role of carbohydrate malabsorption. *Journal of Neurogastroenterology and Motility*. **20** (2), 228-35 (2014).

64. Yang, J. *et al.* Lactose intolerance in irritable bowel syndrome patients with diarrhoea: The roles of anxiety, activation of the innate mucosal immune system and visceral sensitivity. *Alimentary Pharmacology and Therapeutics*. **39** (3), 302-11 (2014).

65. Wang, Y. *et al.* Small intestinal bacterial overgrowth as an uncommon cause of false positive lactose hydrogen breath test among patients with diarrhea-predominant irritable bowel syndrome in Asia. *Journal of Gastroenterology and Hepatology (Australia)*. **30** (6), 995-1000 (2015).

66. Dabak, R. *et al.* The association between irritable bowel syndrome and lactose intolerance. *United European Gastroenterology Journal*. **5** (5), (2017).

67. Lishou, X. *et al.* Prevalence of lactose intolerance in patients with diarrhea-predominant irritable bowel syndrome: data from a tertiary center in southern China. *Journal of Health, Population & Nutrition*. **36**, 1-5 (2017).

68. Kumar, S., Ranjan, P., Mittal, B., Singh, R.& Ghoshal, U. C. Lactase persistence/non-persistence genetic variants in irritable bowel syndrome in an endemic area for lactose malabsorption. *J Gastroenterol Hepatol*. **27** (12), 1825-30 (2012).

69. Yang, J. *et al.* Prevalence and presentation of lactose intolerance and effects on dairy product intake in healthy subjects and patients with irritable bowel syndrome. *Clinical gastroenterology and hepatology*. **11** (3), 262‐8.e1 (2013).

70. Choi, Y. K., Kraft, N., Jackson, M., Rao, S. S. C.& Zimmerman, B. Fructose intolerance in IBS and utility of fructose-restricted diet. *Journal of Clinical Gastroenterology*. **42** (3), 233-8 (2008).

71. Skoog, S. M., Bharucha, A. E.& Zinsmeister, A. R. Comparison of breath testing with fructose and high fructose corn syrups in health and IBS. *Neurogastroenterology and Motility*. **20** (5), 505-11 (2008).

72. Melchior, C., Ducrotte, P., Gourcerol, G., Dechelotte, P.& Leroi, A. M. Symptomatic fructose malabsorption in irritable bowel syndrome: A prospective study. *United European Gastroenterology Journal*. **2** (2), 131-7 (2014).

73. Melchior, C. *et al.* Is abnormal 25 g fructose breath test a predictor of symptomatic response to a low fructose diet in irritable bowel syndrome? *Clinical Nutrition*. (2019).

74. Nelis, G. F., Vermeeren, M. A.& Jansen, W. Role of fructose-sorbitol malabsorption in the irritable bowel syndrome. *Gastroenterology*. **99** (4), 1016-20 (1990).

75. Yao, C. K. *et al.* Dietary sorbitol and mannitol: food content and distinct absorption patterns between healthy individuals and patients with irritable bowel syndrome. *Journal of human nutrition and dietetics*. **27 Suppl 2**, 263‐75 (2014).

76. Chadwick, V. S. *et al.* Activation of the mucosal immune system in irritable bowel syndrome. *Gastroenterology*. **122** (7), 1778-83 (2002).

77. Tuncer, C., Cindoruk, M., Dursun, A.& Karakan, T. Prevalence of microscopic colitis in patients with symptoms suggesting irritable bowel syndrome. *Acta Gastro-Enterologica Belgica*. **66** (2), 133-6 (2003).

78. Tavakkoli, H., Emami, M. H., Esmaeili, F. S., Mahzouni, P.& Haghdani, S. Is microscopic colitis a missed diagnosis in diarrhea-predominant Irritable Bowel Syndrome? *Journal of Research in Medical Sciences*. **13** (4), 202-6 (2008).

79. Chey, W. D. *et al.* The yield of colonoscopy in patients with non-constipated irritable bowel syndrome: results from a prospective, controlled US trial. *Am J Gastroenterol*. **105** (4), 859-65 (2010).

80. El-Salhy, M., Halwe, J., Lomholt-Beck, B.& Gundersen, D. The prevalence of inflammatory bowel diseases, microscopic colitis, and colorectal cancer in patients with irritable bowel syndrome. *Gastroenterology Insights*. **3** (1), 7-10 (2011).

81. Ozdil, K. *et al.* The frequency of microscopic and focal active colitis in patients with irritable bowel syndrome. *BMC Gastroenterology*. **11** (1), 96- (2011).

82. Rahman, M. A. *et al.* Symptomatic overlap in patients with diarrhea predominant irritable bowel syndrome and microscopic colitis in a sub group of Bangladeshi population. *Bangladesh Med Res Counc Bull*. **38** (1), 33-8 (2012).

83. Hilmi, I., Hartono, J. L., Mahadeva, S., Goh, K. L.& Pailoor, J. Low prevalence of 'classical' microscopic colitis but evidence of microscopic inflammation in Asian Irritable Bowel Syndrome patients with diarrhoea. *BMC Gastroenterology*. **13** (1), (2013).

84. Patel, P. *et al.* Prevalence of organic disease at colonoscopy in patients with symptoms compatible with irritable bowel syndrome: cross-sectional survey. *Scand J Gastroenterol*. **50** (7), 816-23 (2015).

85. Simsek, Z. *et al.* Two gastrointestinal conditions with similar symptoms and endoscopic appearance: Irritable bowel syndrome and microscopic colitis. *Turkish Journal of Medical Sciences*. **45** (2), 393-7 (2015).

86. Ucmak, F., Goral, V., Firat, U.& Mete, N. Rate of microscopic colitis and cytokine levels in patients with irritable bowel syndrome. **31** (1), 103-8 (2015).

87. Hilpusch, F. *et al.* Microscopic colitis: a missed diagnosis among patients with moderate to severe irritable bowel syndrome. *Scand J Gastroenterol*. **52** (2), 173-7 (2017).

88. Johnsen, P. H. *et al.* Faecal microbiota transplantation versus placebo for moderate-to-severe irritable bowel syndrome: a double-blind, randomised, placebo-controlled, parallel-group, single-centre trial. *The lancet Gastroenterology & hepatology*. **3** (1), 17‐24 (2018).

89. Paudel, M. S. *et al.* Prevalence of organic colonic lesions by colonoscopy in patients fulfilling ROME IV criteria of irritable bowel syndrome. *Journal of the Nepal Medical Association*. **56** (209), 487-92 (2018).

90. El-Salhy, M., Gilja, O. H.& Hatlebakk, J. G. Overlapping of irritable bowel syndrome with erosive esophagitis and the performance of Rome criteria in diagnosing IBS in a clinical setting. *Molecular Medicine Reports*. **20** (1), 787-94 (2019).

91. Asghar, Z. *et al.* Diagnostic Yield of Colonoscopy in Patients with Symptoms Compatible with Rome IV Functional Bowel Disorders. *Clinical gastroenterology and hepatology : the official clinical practice journal of the American Gastroenterological Association*. (2020).

92. Leeds, J. S. *et al.* Some patients with irritable bowel syndrome may have exocrine pancreatic insufficiency. *Clin Gastroenterol Hepatol*. **8** (5), 433-8 (2010).

93. Talley, N. J. *et al.* Undiagnosed pancreatic exocrine insufficiency and chronic pancreatitis in functional GI disorder patients with diarrhea or abdominal pain. *Journal of Gastroenterology and Hepatology (Australia)*. **32** (11), 1813-7 (2017).

94. Pimentel, M., Chow, E. J.& Lin, H. C. Eradication of small intestinal bacterial overgrowth reduces symptoms of irritable bowel syndrome. *Am J Gastroenterol*. **95** (12), 3503-6 (2000).

95. Pimentel, M., Chow, E. J.& Lin, H. C. Normalization of lactulose breath testing correlates with symptom improvement in irritable bowel syndrome. a double-blind, randomized, placebo-controlled study. *Am J Gastroenterol*. **98** (2), 412-9 (2003).

96. Pimentel, M. *et al.* A link between irritable bowel syndrome and fibromyalgia may be related to findings on lactulose breath testing. *Ann Rheum Dis*. **63** (4), 450-2 (2004).

97. Lupascu, A. *et al.* Hydrogen glucose breath test to detect small intestinal bacterial overgrowth: a prevalence case-control study in irritable bowel syndrome. *Aliment Pharmacol Ther*. **22** (11-12), 1157-60 (2005).

98. Sharara, A. I. *et al.* A randomized double-blind placebo-controlled trial of rifaximin in patients with abdominal bloating and flatulence. *Am J Gastroenterol*. **101** (2), 326-33 (2006).

99. Esposito, I. *et al.* Breath test for differential diagnosis between small intestinal bacterial overgrowth and irritable bowel disease: an observation on non-absorbable antibiotics. *World J Gastroenterol*. **13** (45), 6016-21 (2007).

100. Majewski, M.& McCallum, R. W. Results of small intestinal bacterial overgrowth testing in irritable bowel syndrome patients: clinical profiles and effects of antibiotic trial. *Adv Med Sci*. **52**, 139-42 (2007).

101. Posserud, I., Stotzer, P. O., Bjornsson, E. S., Abrahamsson, H.& Simren, M. Small intestinal bacterial overgrowth in patients with irritable bowel syndrome. *Gut*. **56** (6), 802-8 (2007).

102. Carrara, M. *et al.* Small intestine bacterial overgrowth in patients with irritable bowel syndrome. *Eur Rev Med Pharmacol Sci*. **12** (3), 197-202 (2008).

103. Grover, M. *et al.* Small intestinal bacterial overgrowth in irritable bowel syndrome: association with colon motility, bowel symptoms, and psychological distress. *Neurogastroenterol Motil*. **20** (9), 998-1008 (2008).

104. Law, D.& Pimentel, M. Proton pump inhibitor therapy does not affect hydrogen production on lactulose breath test in subjects with IBS. *Dig Dis Sci*. **55** (8), 2302-8 (2010).

105. Lombardo, L., Foti, M., Ruggia, O.& Chiecchio, A. Increased incidence of small intestinal bacterial overgrowth during proton pump inhibitor therapy. *Clin Gastroenterol Hepatol*. **8** (6), 504-8 (2010).

106. Reddymasu, S. C., Sostarich, S.& McCallum, R. W. Small intestinal bacterial overgrowth in irritable bowel syndrome: are there any predictors? *BMC Gastroenterology*. **10**, 23- (2010).

107. Kim, E. J. *et al.* The characteristics of the positivity to the lactulose breath test in patients with abdominal bloating. *European Journal of Gastroenterology & Hepatology*. **23** (12), 1144-9 (2011).

108. Sachdeva, S., Rawat, A. K., Reddy, R. S.& Puri, A. S. Small intestinal bacterial overgrowth (SIBO) in irritable bowel syndrome: frequency and predictors. *J Gastroenterol Hepatol*. **26 Suppl 3**, 135-8 (2011).

109. Youn, Y. H. *et al.* Relationships among the lactulose breath test, intestinal gas volume, and gastrointestinal symptoms in patients with irritable bowel syndrome. *Digestive Diseases and Sciences*. **56** (7), 2059-66 (2011).

110. Meyrat, P., Safroneeva, E.& Schoepfer, A. M. Rifaximin treatment for the irritable bowel syndrome with a positive lactulose hydrogen breath test improves symptoms for at least 3 months. *Aliment Pharmacol Ther*. **36** (11-12), 1084-93 (2012).

111. Rana, S. V., Sharma, S., Kaur, J., Sinha, S. K.& Singh, K. Comparison of lactulose and glucose breath test for diagnosis of small intestinal bacterial overgrowth in patients with irritable bowel syndrome. *Digestion*. **85** (3), 243-7 (2012).

112. Lee, K. N. *et al.* Association between symptoms of irritable bowel syndrome and methane and hydrogen on lactulose breath test. *J Korean Med Sci*. **28** (6), 901-7 (2013).

113. Dupont, A. W. *et al.* Motility abnormalities in irritable bowel syndrome. *Digestion*. **89** (2), 119-23 (2014).

114. Ghoshal, U. C., Srivastava, D., Ghoshal, U.& Misra, A. Breath tests in the diagnosis of small intestinal bacterial overgrowth in patients with irritable bowel syndrome in comparison with quantitative upper gut aspirate culture. *European Journal of Gastroenterology & Hepatology*. **26** (7), 753-60 (2014).

115. Srivastava, D., Ghoshal, U., Mittal, R. D.& Ghoshal, U. C. Associations between IL-1RA polymorphisms and small intestinal bacterial overgrowth among patients with irritable bowel syndrome from India. *Neurogastroenterol Motil*. **26** (10), 1408-16 (2014).

116. Abbasi, M. H. B., Zahedi, M. J., Moghadam, S. D.& Shafieipour, S. Small bowel bacterial overgrowth in patients with irritable bowel syndrome: The first study in Iran. *Middle East Journal of Digestive Diseases*. **7** (1), 36-40 (2015).

117. Chu, H. *et al.* Small Intestinal Bacterial Overgrowth in Patients with Irritable Bowel Syndrome: Clinical Characteristics, Psychological Factors, and Peripheral Cytokines. *Gastroenterology Research and Practice*. **2016**, (2016).

118. Nagasako, C. K. *et al.* Prolonged orocecal transit time is associated with small intestinal bacterial overgrowth in irritable bowel syndrome in a tertiary referral hospital in Brazil. *Acta Gastroenterologica Latinoamericana*. **46** (4), 314-21 (2016).

119. Ning, Y. *et al.* Clinical value of radionuclide small intestine transit time measurement combined with lactulose hydrogen breath test for the diagnosis of bacterial overgrowth in irritable bowel syndrome. *Hell J Nucl Med*. **19** (2), 124-9 (2016).

120. Jung, S. E., Joo, N. S., Han, K. S.& Kim, K. N. Obesity Is Inversely Related to Hydrogen-Producing Small Intestinal Bacterial Overgrowth in Non-Constipation Irritable Bowel Syndrome. *J Korean Med Sci*. **32** (6), 948-53 (2017).

121. Huang, Y., Jia, L.& Liu, Y. The relationships between hydrogen gas and methane gas and the symptoms of irritable bowel syndrome as determined by a breath test. *International Journal of Clinical and Experimental Medicine*. **12** (6), 7356-64 (2019).

122. Lee, S.-H. *et al.* Effect of eradicating hydrogen-forming small intestinal bacterial overgrowth with rifaximin on body weight change. *Medicine*. **98** (51), 1-8 (2019).

123. Leventogiannis, K. *et al.* Effect of a Preparation of Four Probiotics on Symptoms of Patients with Irritable Bowel Syndrome: Association with Intestinal Bacterial Overgrowth. *Probiotics and Antimicrobial Proteins*. **11** (2), 627-34 (2019).

124. Rezaie, A., Heimanson, Z., McCallum, R.& Pimentel, M. Lactulose Breath Testing as a Predictor of Response to Rifaximin in Patients With Irritable Bowel Syndrome With Diarrhea. *Am J Gastroenterol*. **114** (12), 1886-93 (2019).

125. Wu, K. Q. *et al.* Small intestinal bacterial overgrowth is associated with Diarrhea-predominant irritable bowel syndrome by increasing mainly Prevotella abundance. *Scand J Gastroenterol*. **54** (12), 1419-25 (2019).

126. Zhuang, X., Tian, Z., Luo, M.& Xiong, L. Short-course Rifaximin therapy efficacy and lactulose hydrogen breath test in Chinese patients with diarrhea-predominant irritable bowel syndrome. *BMC Gastroenterol*. **20** (1), 187 (2020).

127. Rana, S. V., Sinha, S. K., Sikander, A., Bhasin, D. K.& Singh, K. Study of small intestinal bacterial overgrowth in North Indian patients with irritable bowel syndrome: a case control study. *Trop Gastroenterol*. **29** (1), 23-5 (2008).
